# Safety, immunogenicity, and efficacy of the candidate tuberculosis vaccine MVA85A in healthy adults infected with HIV-1: a randomised, placebo-controlled, phase 2 trial

**DOI:** 10.1016/S2213-2600(15)00037-5

**Published:** 2015-03

**Authors:** Birahim Pierre Ndiaye, Friedrich Thienemann, Martin Ota, Bernard S Landry, Makhtar Camara, Siry Dièye, Tandakha Ndiaye Dieye, Hanif Esmail, Rene Goliath, Kris Huygen, Vanessa January, Ibrahima Ndiaye, Tolu Oni, Michael Raine, Marta Romano, Iman Satti, Sharon Sutton, Aminata Thiam, Katalin A Wilkinson, Souleymane Mboup, Robert J Wilkinson, Helen McShane, for the MVA85A 030 trial investigators

**Affiliations:** a Laboratoire de Bactériologie–Virologie, Centre Hospitalier Universitaire Le Dantec, Dakar, Senegal; b Clinical Infectious Diseases Research Initiative, Institute of Infectious Disease and Molecular Medicine, University of Cape Town, Cape Town, South Africa; c Division of Public Health Medicine, School of Public Health and Family Medicine, University of Cape Town, Cape Town, South Africa; d Department of Medicine, University of Cape Town, Cape Town, South Africa; e Medical Research Council Unit, Fajara, The Gambia; f Aeras, Rockville, MD, USA; g Immunology Service, Scientific Institute of Public Health (WIV-ISP), Brussels, Belgium; h Centre de Traitement Ambulatoire, Centre Hospitalier Universitaire de Fann, Dakar, Senegal; i Jenner Institute, University of Oxford, Oxford, UK; j Department of Medicine, Imperial College London, London, UK; k MRC National Institute for Medical Research, London, UK

## Abstract

**Background:**

HIV-1 infection is associated with increased risk of tuberculosis and a safe and effective vaccine would assist control measures. We assessed the safety, immunogenicity, and efficacy of a candidate tuberculosis vaccine, modified vaccinia virus Ankara expressing antigen 85A (MVA85A), in adults infected with HIV-1.

**Methods:**

We did a randomised, double-blind, placebo-controlled, phase 2 trial of MVA85A in adults infected with HIV-1, at two clinical sites, in Cape Town, South Africa and Dakar, Senegal. Eligible participants were aged 18–50 years, had no evidence of active tuberculosis, and had baseline CD4 counts greater than 350 cells per μL if they had never received antiretroviral therapy or greater than 300 cells per μL (and with undetectable viral load before randomisation) if they were receiving antiretroviral therapy; participants with latent tuberculosis infection were eligible if they had completed at least 5 months of isoniazid preventive therapy, unless they had completed treatment for tuberculosis disease within 3 years before randomisation. Participants were randomly assigned (1:1) in blocks of four by randomly generated sequence to receive two intradermal injections of either MVA85A or placebo. Randomisation was stratified by antiretroviral therapy status and study site. Participants, nurses, investigators, and laboratory staff were masked to group allocation. The second (booster) injection of MVA85A or placebo was given 6–12 months after the first vaccination. The primary study outcome was safety in all vaccinated participants (the safety analysis population). Safety was assessed throughout the trial as defined in the protocol. Secondary outcomes were immunogenicity and vaccine efficacy against *Mycobacterium tuberculosis* infection and disease, assessed in the per-protocol population. Immunogenicity was assessed in a subset of participants at day 7 and day 28 after the first and second vaccination, and *M tuberculosis* infection and disease were assessed at the end of the study. The trial is registered with ClinicalTrials.gov, number NCT01151189.

**Findings:**

Between Aug 4, 2011, and April 24, 2013, 650 participants were enrolled and randomly assigned; 649 were included in the safety analysis (324 in the MVA85A group and 325 in the placebo group) and 645 in the per-protocol analysis (320 and 325). 513 (71%) participants had CD4 counts greater than 300 cells per μL and were receiving antiretroviral therapy; 136 (21%) had CD4 counts above 350 cells per μL and had never received antiretroviral therapy. 277 (43%) had received isoniazid prophylaxis before enrolment. Solicited adverse events were more frequent in participants who received MVA85A (288 [89%]) than in those given placebo (235 [72%]). 34 serious adverse events were reported, 17 (5%) in each group. MVA85A induced a significant increase in antigen 85A-specific T-cell response, which peaked 7 days after both vaccinations and was primarily monofunctional. The number of participants with negative QuantiFERON-TB Gold In-Tube findings at baseline who converted to positive by the end of the study was 38 (20%) of 186 in the MVA85A group and 40 (23%) of 173 in the placebo group, for a vaccine efficacy of 11·7% (95% CI −41·3 to 44·9). In the per-protocol population, six (2%) cases of tuberculosis disease occurred in the MVA85A group and nine (3%) occurred in the placebo group, for a vaccine efficacy of 32·8% (95% CI −111·5 to 80·3).

**Interpretation:**

MVA85A was well tolerated and immunogenic in adults infected with HIV-1. However, we detected no efficacy against *M tuberculosis* infection or disease, although the study was underpowered to detect an effect against disease. Potential reasons for the absence of detectable efficacy in this trial include insufficient induction of a vaccine-induced immune response or the wrong type of vaccine-induced immune response, or both.

**Funding:**

European & Developing Countries Clinical Trials Partnership (IP.2007.32080.002), Aeras, Bill & Melinda Gates Foundation, Wellcome Trust, and Oxford-Emergent Tuberculosis Consortium.

**Research in context**

**Evidence before this study**

One previous study assessed the efficacy of several doses of the saprophyte *Mycobacterium vaccae* against tuberculosis disease in adults infected with HIV-1, and showed a decreased risk of protocol-defined pulmonary tuberculosis. A previous study with the MVA85A, the candidate vaccine under assessment here, has showed that boosting with MVA85A did not enhance protective efficacy in BCG-vaccinated infants. Adults infected with HIV-1 are an important target population for a new tuberculosis vaccine, and in earlier studies, vaccine-induced immunogenicity in adults infected with HIV-1 was higher than in infants.

**Added value of this study**

This is the first time that a candidate tuberculosis vaccine has been assessed for efficacy against *Mycobacterium tuberculosis* infection in people infected with HIV-1. The results show that vaccinating adults infected with HIV-1 with MVA85A is safe, but does not confer protection against infection with *M tuberculosis*.

**Implications of all the available evidence**

The safety of MVA85A in this large study population of adults with HIV infection is an important finding for tuberculosis vaccine development. The vector is safe to give to people without HIV testing; these safety data provide some generic reassurance that new candidate tuberculosis vaccines are safe in this higher risk population. Additionally, this study has shown that high-quality, multicentre tuberculosis vaccine trials in vulnerable populations are possible. The absence of efficacy despite immunogenicity in this and previous clinical trials of MVA85A suggests that the current parameters for selection of tuberculosis vaccine candidates are inadequate. Standardised preclinical animal models that better represent human infection and disease, and a greater understanding of immune mechanisms of protection in human tuberculosis are both urgently needed. Alternative approaches to vaccine development, including the delivery of candidate vaccines direct to the respiratory mucosa, merit assessment. Other lessons learnt from this trial include the characterisation of the epidemiology of *M tuberculosis* infection and disease associated with HIV-1 infection in a setting of antiretroviral therapy and isoniazid chemoprophylaxis.

## Introduction

Tuberculosis is a substantial global cause of mortality and morbidity, with 9 million new cases of active tuberculosis and 1·5 million deaths occurring in 2013.^1^ One third of the world's population is infected with *Mycobacterium tuberculosis*.^1^ HIV-1 co-infection is one of the most important risk factors for both infection with *M tuberculosis* and active tuberculosis disease,^2^ with an estimated 1·1 million of all new tuberculosis cases in 2013 occurring in people co-infected with HIV-1.^1^ The WHO African region accounts for 80% of HIV-1-associated tuberculosis.^1^ Additionally, the growing incidence of drug-resistant tuberculosis is associated with poor treatment outcome and increased mortality.^3^ The global Stop TB Partnership aims to eliminate tuberculosis as a public health problem by 2050. An agreed major component to advance this aim would be an effective vaccine.^4^ BCG is the only licensed tuberculosis vaccine—it provides protection against severe childhood tuberculosis,^5^, ^6^ but the protection conferred against pulmonary tuberculosis in adults and adolescents is highly variable.^7^, ^8^

At least 16 candidate tuberculosis vaccines have advanced to clinical testing.^9^ The modified vaccinia virus Ankara expressing the major *M tuberculosis* antigen 85A (MVA85A) is a clinically advanced candidate vaccine.^10^, ^11^, ^12^ MVA85A is well tolerated and immunogenic in adults infected and not infected with HIV-1, and in infants not exposed to HIV-1.^10^, ^11^, ^12^, ^13^, ^14^ MVA85A adds to BCG-induced protection against mycobacterial challenge in some preclinical animal models.^15^, ^16^, ^17^, ^18^, ^19^ However, boosting BCG with MVA85A in South African infants not infected with HIV-1 did not confer additional protection against tuberculosis disease or *M tuberculosis* infection.^10^

Administration of several doses of the saprophyte *Mycobacterium vaccae* to adults infected with HIV-1 was associated with a decreased risk of protocol-defined pulmonary tuberculosis,^20^ suggesting that vaccination might be effective in people infected with HIV-1. Here we report the results of a multisite, randomised, placebo-controlled, phase 2 trial to assess the safety, immunogenicity, and efficacy of MVA85A in healthy adults infected with HIV-1.

## Methods

### Study design and participants

We did a proof-of-concept, randomised, double-blind, placebo-controlled, phase 2 trial of MVA85A at two clinical sites, in Cape Town, South Africa and Dakar, Senegal. In Cape Town, participants were recruited in the community and from primary care clinics in Khayelitsha by use of radio and newspaper advertisements, flyers, pamphlets, and information campaigns at the clinics. Khayelitsha is a densely populated, low-income, peri-urban township. In 2010, antenatal HIV-1 prevalence was 33% and the tuberculosis case notification rate was at least 1500 per 100 000 population per year.^21^ In Dakar, participants were recruited from public service HIV clinics at the Centre de Traitement Ambulatoire and the Centre de Recherche Clinique et de Formation, Centre Hospitalier Universitaire de Fann. Senegal had an estimated HIV-1 prevalence in adults of less than 1% in 2012, and a reported tuberculosis incidence rate of 0·14% in 2013.^1^ The annual rate of *M tuberculosis* infection has not previously been estimated at either site. Eligibility criteria included participants aged 18–50 years with no evidence of active tuberculosis, and baseline CD4 counts greater than 350 cells per μL if they were not receiving antiretroviral therapy, or greater than 300 cells per μL (and with undetectable viral load before randomisation) if they were receiving antiretroviral therapy. Participants with latent tuberculosis infection were eligible for enrolment if they had completed at least 5 months of isoniazid preventive therapy, unless they had completed treatment for tuberculosis disease within 3 years before randomisation. The complete inclusion criteria are listed in the study protocol (appendix).

The trial adhered to International Conference on Harmonisation Good Clinical Practice guidelines, and was approved by the University of Cape Town's Faculty of Health Sciences Human Research Ethics Committee and the Medicines Control Council of South Africa; the Senegalese National Ethics Committee for Research in Health; and the Oxford University Tropical Research Ethics Committee. All participants provided written informed consent before any study procedure.

### Randomisation and masking

Participants were randomly assigned (1:1) in blocks of four by a randomly generated sequence of participant identification numbers via an interactive voice response system to receive two intradermal injections of either 1 × 10^8^ pfu MVA85A or placebo (*Candida* skin test antigen [Candin], Allermed Laboratories, San Diego, CA, USA). Randomisation was stratified by antiretroviral therapy status and study site. A statistician uninvolved with study analyses prepared the interactive voice response system randomisation schedule. Doses of vaccines were prepared and labelled in masked syringes by a pharmacist unmasked to group allocation. Participants, nurses (who were involved in assessment and follow-up), investigators, and laboratory staff were masked to group allocation. The second (booster) injection of MVA85A or placebo was given 6–12 months after the first vaccination and participants were actively followed up every 3 months until the last participant enrolled had completed 6 months of follow-up after the booster vaccination.

### Procedures

We collected data for the incidence of solicited and unsolicited adverse events, including both local injection-site reactions and systemic reactions. Participants reported solicited adverse events on diary cards for 7 days after each vaccination and in response to direct questioning by trained study staff on days 7 and 28 after each injection. Phlebotomy for routine haematological and biochemical analysis was done at screening, before booster vaccination, and on days 7 and 28 after each vaccination. Peripheral CD4 cell count and HIV-1 viral load were also measured at these timepoints and every 3 months until 6 months after booster vaccination. Serious adverse events were monitored by active surveillance throughout and until the end of the trial. The site investigators and local medical monitors determined the severity and seriousness of adverse events and the relation of these to the vaccine. An independent data monitoring committee assessed masked group safety data after 200 participants had been enrolled and unmasked after 600 participants had been enrolled.

In a prespecified subset of 70 participants (35 from each group), immunology samples were obtained before each vaccination and on days 7 and 28 after each vaccination. All immunology tests were done masked to group allocation. We assessed vaccine immunogenicity with three assays. First, ex vivo interferon γ enzyme-linked immunospot (ELISPOT) analysis was done on fresh peripheral blood mononuclear cells.^22^ Cells were stimulated overnight with a single pool of 66 peptides of the antigen 85A (Ag85A), ESAT-6, and CFP-10. Second, Ag85A-specific intracellular cytokine staining assay was done on whole blood.^23^ Stimulated fixed whole blood samples were stained for CD3-positive, CD4-positive, CD8-positive, CD14-positive, and CD19-positive cells, interferon γ, tumour necrosis factor α, interleukin 17, and interleukin 2. Third, Ag85A-specific antibody response was measured on plasma. Ag85A-specific immunoglobulin G (IgG) antibodies were measured by ELISA on eight serial two-fold dilutions of plasma (1:25–1:3200), by use of affinity purified recombinant, histidine-tagged Ag85A^24^ (microwell plates coated with 50 ng per well of recombinant Ag85A in borate buffer, overnight at 4°C). Alkaline phosphatase-labelled goat anti-human IgG (Sigma, St Louis, MO, USA) was used as secondary antibody at a dilution of 1:1000 and optical density was read at 405 nm after development with phosphatase substrate (Sigma). Results were expressed in arbitrary units per mL (AU/mL), as compared with values of an internal tuberculosis serum standard of 2500 AU/mL.

Participants were screened to exclude active tuberculosis by symptom screen and chest radiography at both sites before enrolment. In Cape Town, participants also underwent sputum collection for tuberculosis smear microscopy, GeneXpert MTB/RIF (Cepheid, Sunnyvale, CA, USA), and mycobacterial liquid culture (MGIT; Becton Dickinson, Sparks, MD, USA) because of previously documented high frequencies of asymptomatic disease at this site.^25^ Latent *M tuberculosis* infection was defined as either a positive QuantiFERON-TB Gold In-Tube (QFT) test or a tuberculin purified protein derivative skin test (tuberculin skin test) reaction greater than 5 mm.

Participants were monitored throughout the trial for possible tuberculosis. Tuberculosis investigations were done in participants who had been in contact with a known case of active tuberculosis, in those who presented with at least one of cough for more than 1 week, fever for more than 1 week, drenching night sweats, unintentional weight loss of more than 3 kg, pleuritic chest pains, haemoptysis, or shortness of breath; and in those who converted to a positive QFT or tuberculin skin test (≤5 mm to >5 mm). Investigations included clinical examination, chest radiography, and collection of at least two sputum samples on which tuberculosis smear microscopy, GeneXpert MTB/RIF, and mycobacterial liquid culture were done. Chest radiographs were reviewed by two physicians, with a third reading to achieve consensus in the event of disagreement. QFT and tuberculin skin tests were repeated at the final study visit.

### Outcomes

Tuberculosis disease endpoint 1 was defined as culture or GeneXpert MTB/RIF positivity; endpoint 2 included endpoint 1 and a composite clinical endpoint (which included a single acid-fast bacilli smear from a sterile body site; two smears from pulmonary and gastric sampling, and compatible clinical symptoms and radiological signs); and endpoint 3 was participant commencement on anti-tubercular chemotherapy (see the study protocol for more information; appendix). The *M tuberculosis* infection endpoint was defined as conversion from negative QFT at baseline to positive QFT at the final visit.

The primary study outcome was the safety of MVA85A in all participants who received at least one dose of study vaccine or placebo (the safety analysis population) as determined by the numbers and percentages of adverse events (including solicited, unsolicited, and serious adverse events).

The secondary outcome was the efficacy of MVA85A for the prevention of active tuberculosis in the per-protocol population (all randomly allocated participants who received at least one dose of study vaccine or placebo and had no major protocol deviations and no tuberculosis case definition endpoints within 28 days after study day 0 [first vaccination]), which was determined by the incidence of active tuberculosis meeting the definition of endpoint 1, calculated as the number of new cases of active tuberculosis with a date of diagnosis from 28 days after the first vaccination until the end of the study follow-up (May 19, 2014). An intention-to-treat analysis was also done for disease efficacy. In the per-protocol population, we also examined the efficacy of MVA85A by antiretroviral therapy status at the time of randomisation and by baseline isoniazid preventive therapy status.

Other secondary outcomes were to assess CD4-positive lymphocyte counts and HIV-1 viral load before and after administration of MVA85A compared with placebo; to assess the immunogenicity of MVA85A compared with placebo as measured by the ex-vivo interferon γ ELISPOT assay; to assess the immunogenicity of MVA85A compared with placebo as measured by flow cytometric intracellular cytokine staining of CD4-positive and CD8-positive T cells after stimulation with a peptide pool of mycobacterial antigens; to identify potential immunological correlates of protection from tuberculosis in participants vaccinated with MVA85A and to assess the QFT conversion rate at final study assessment in MVA85A recipients compared with controls without a diagnosis of tuberculosis during the trial.

### Statistical analysis

All sample size calculations assumed a loss to follow-up and death rate of 2%. The initial planned sample size for this trial was 1400 adult participants, to be followed up for 2 years after the last participant was enrolled. This sample size provided 80% power to detect a vaccine efficacy of 60% against tuberculosis disease. However, after review of the phase 2 infant efficacy data,^10^ the trial design was revised with safety as the primary objective and a smaller sample size and shorter follow-up of a minimum of 6 months. The revised sample size for this study was selected as adequate for a review of the safety profile. With 325 participants assigned to receive MVA85A, the revised sample would have a 90% probability of detecting at least one adverse event occurring at a rate of 0·71%. Because of the expected effect of antiretroviral therapy on tuberculosis disease, an estimated tuberculosis disease incidence ranging between 1·5% and 2% per year was used to estimate the power of the revised sample size for efficacy. Calculations were based on a one-sided log-rank test at a significance level of 0·10 and assumed completion of enrolment in 21 months, a follow-up period of about 15 months for the last patient enrolled, and a maximum of 36 months for the first patient enrolled. If the true efficacy was about 70%, 325 patients per treatment group (650 patients total) provided 81% power to show positive efficacy given an incidence rate of 2·0% in the control group per year, or 71% power given an incidence rate of 1·5% in the control group per year. At a true efficacy of about 60%, 325 patients per treatment group provided 67% power to show positive efficacy given an incidence rate of 2·0% per year, or 57% power given an incidence rate of 1·5% per year. Vaccine efficacy to prevent infection was a secondary endpoint: the recorded QFT conversion rate in the study provided 80% power to detect a vaccine efficacy of 50%.

Statistical analyses were done using SAS version 9.2. All analyses were prespecified in the statistical analysis plan before locking of the database. For the safety analysis, we compared the proportion of participants with at least one adverse event in the MVA85A group versus those in the placebo using Fisher's exact test. We also calculated two-sided 95% CIs for proportions of adverse events within treatment groups and the differences between groups.

The main statistical method used in the analysis of tuberculosis disease endpoints 1–3 was vaccine efficacy, estimated as 1 minus the estimated hazard ratio, based on a Cox regression analysis of time (days) to initial tuberculosis diagnosis, based on the per-protocol population. As supportive confirmatory analysis, we used the conditional binomial (Clopper-Pearson) method to estimate vaccine efficacy and its corresponding two-sided 95% CIs and p values. Time to initial diagnosis for each endpoint was compared by use of a two-sided log-rank test, stratified by study site and antiretroviral therapy status at randomisation. Analyses were summarised by antiretroviral therapy and treatment group for participants in the per-protocol population. Vaccine efficacy against *M tuberculosis* infection and the corresponding 95% CI, and p value were calculated with the conditional binomial method (Clopper-Pearson), identical to the tuberculosis case definition endpoint analysis.

Other secondary endpoints were analysed in various ways. Median CD4 cell counts and associated two-sided 95% CIs were summarised by antiretroviral therapy status at randomisation, study site, treatment group, and timepoint. HIV-1 viral load (copies per mL) was summarised with medians (and associated 95% CIs) by antiretroviral therapy status at randomisation, study site, and treatment group, at each available timepoint. Both the CD4 cell counts and HIV-1 viral load values were log-transformed before any analysis was done. We used Wilcoxon paired analysis to compare within group before and after vaccination responses.

Quintiles (Blomfontein, South Africa) did the statistical analysis, and Aeras paid for this service. The trial was registered with ClinicalTrials.gov, number NCT01151189.

### Role of the funding source

Aeras was the trial sponsor and contributed to study design and data analysis. The other funders had no role in study design, data collection, data analysis, data interpretation, or writing of the report. BPN, FT, BSL, RJW, and HM had full access to all the data in the study. HM had final responsibility for the decision to submit for publication.

## Results

**Figure 1:**
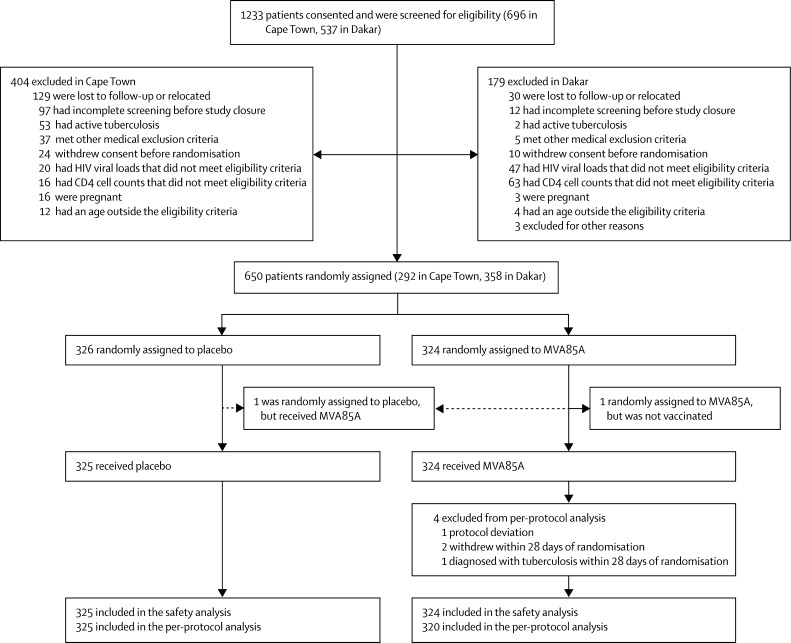
Trial profile

Between Aug 4, 2011, and April 24, 2013, 1233 adults infected with HIV-1 were screened and 650 were randomly assigned; 649 were included in the safety analysis and 645 in the per-protocol analysis (figure 1). 513 (71%) participants had CD4 counts greater than 300 cells per μL and were receiving antiretroviral therapy; 136 (21%) had CD4 counts above 350 cells per μL and had never received antiretroviral therapy. The results of the intention-to-treat analysis were not different and are not reported. 311 (96%) participants in the placebo group and 298 (92%) in the MVA85A group received the booster vaccination. One participant was randomly assigned to placebo but received MVA85A; this participant was included in the safety population for MVA85A but not in the per-protocol efficacy population. One participant was randomly assigned to MVA85A but withdrew consent before vaccination and was not vaccinated. This participant was excluded from both the safety and per-protocol populations. Baseline demographic characteristics were similar in the two study groups and across the two study sites (table 1; appendix). 625 participants completed the study; 14 participants were lost to follow-up (nine placebo, five MVA85A), five withdrew consent (two placebo, three MVA85A), and six died (four placebo, two MVA85A).

In the per-protocol population, median follow-up was 655 days for the 320 recipients of MVA85A and 654 days for the 325 placebo participants. Other than the four participants shown in figure 1, all participants were included in the analysis.

At least one adverse event was reported in 312 (96%) of placebo recipients and 321 (99%) of MVA85A recipients (table 2). Solicited adverse events were more common in participants who received MVA85A than placebo (table 2). Most of these events were local injection-site reactions; other solicited adverse events included mild influenza-like symptoms and regional lymphadenopathy. We noted no significant difference between study groups in the frequency of serious adverse events. 34 serious adverse events occurred during the study, 17 in the placebo group and 17 in the MVA85A group (table 2; appendix). All but one of these events were judged to be unrelated to vaccination; a case of probable tuberculous meningitis that occurred 6 days after vaccination was judged to be possibly related to vaccination. The data monitoring committee reviewed this case, did not request unmasking, and recommended continuing with the study. The participant was treated for tuberculous meningitis and made a full recovery. At study completion, this participant was identified as having received MVA85A. 13 serious adverse events in the infections and infestations category occurred during the study (the only category with more than five serious adverse events in either group), eight in the placebo group and five in the MVA85A group; this difference was not significant (Fisher's exact test, p=0·58).

The frequency of severe adverse events did not differ significantly between study groups (table 2). We noted no significant changes in CD4 cell count or HIV-1 viral load throughout the course of the trial in either study group (data not shown). Routine haematological and biochemical test results did not differ between study groups (data not shown).

**Figure 2:**
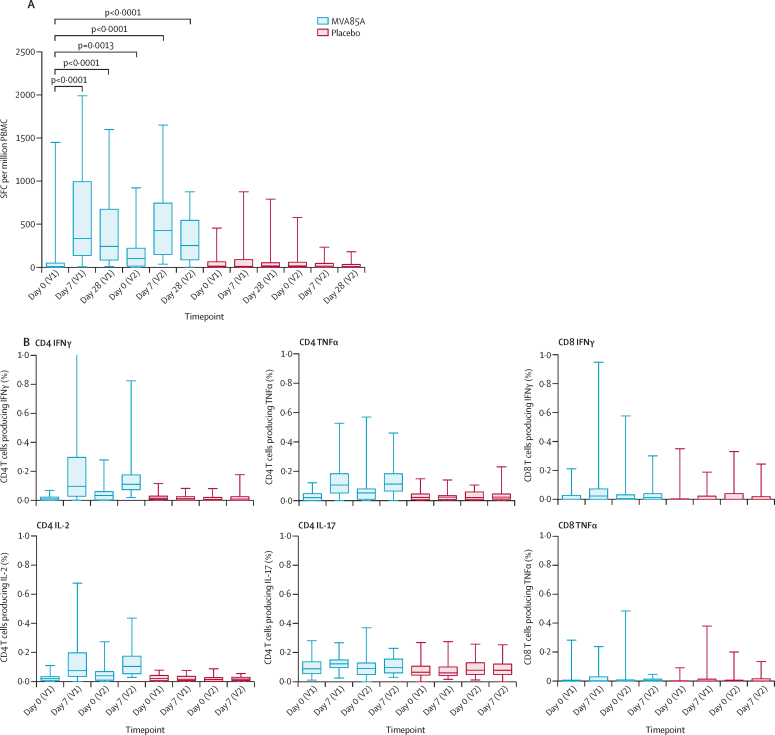
Vaccine immunogenicity (both study sites combined) (A) Antigen 85A (Ag85A) interferon γ enzyme-linked immunospot analysis responses. Data are presented as spot-forming cells (SFC) per million peripheral blood mononuclear cells (PBMCs). p values were calculated with Wilcoxon matched-pair signed-rank tests. Box and whisker plots show median, IQR, and minimum and maximum values. (B) Whole blood intracellular cytokine staining assay of total cytokines. Data are presented as frequency of CD4 and CD8 T cells producing cytokines. Box and whisker plots show median, IQR, and minimum and maximum values. IFNγ=interferon γ. TNFα=tumour necrosis factor α. IL=interleukin. V1=vaccination 1. V2=vaccination 2.

ELISPOT responses to Ag85A were significantly higher in participants from Dakar than in those from Cape Town at baseline (p=0·0016), but at no other timepoint. This difference was not seen with the less sensitive whole blood intracellular cytokine staining assay. MVA85A induced an Ag85A-specific T-cell response that peaked 7 days after the first and booster vaccinations (median spots per million: day 0 [first vaccination], 9·0 [IQR 2·3–51·0]; day 7 [first vaccination], 337·0 [139·3–993·8]; day 0 [booster vaccination], 103·5 [14·8–223·8]; day 7 [booster vaccination], 426·0 [150·0–745·0]; figure 2). Responses at each timepoint after vaccination did not differ by study site or by antiretroviral therapy status. Medians in the placebo group did not exceed 20 spots per million at any timepoint.

**Figure 3:**
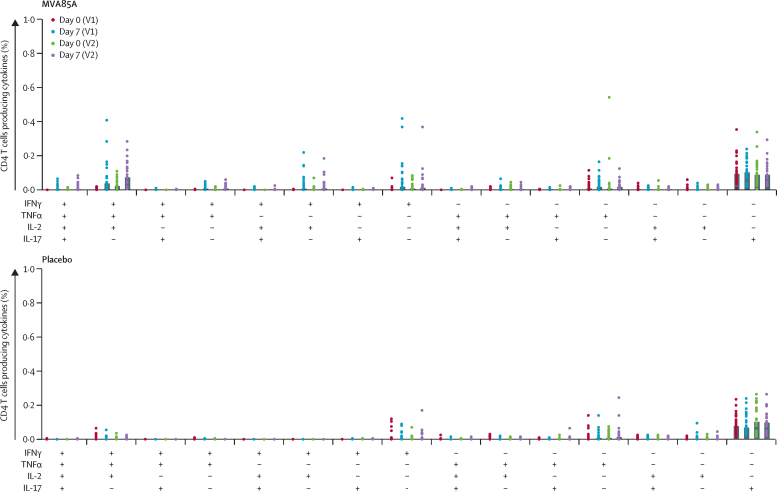
Polyfunctional CD4 T cells Plots show frequency of CD4 T cells producing combinations of the studied cytokines. Bars are median values and dots represent individual volunteers. IFNγ=interferon γ. TNFα=tumour necrosis factor α. IL=interleukin. V1=vaccination 1. V2=vaccination 2.

Whole blood intracellular cytokine staining showed the most commonly measured cytokine from CD4 T cells was interferon γ, in agreement with the ELISPOT data. Tumour necrosis factor α and low concentrations of interleukins 2 and 17 were also detected (table 3, figure 2). Overall, numbers of antigen-specific CD8 T cells were very low and were only positive for interferon γ and tumour necrosis factor α. Multiparameter flow-cytometric analysis showed that mainly monofunctional Ag85A-specific CD4 T cells were present before and after vaccination (figure 3). Ag85A-specific antibody responses were less than twice the baseline value after vaccination in all but three participants.

**Figure 4:**
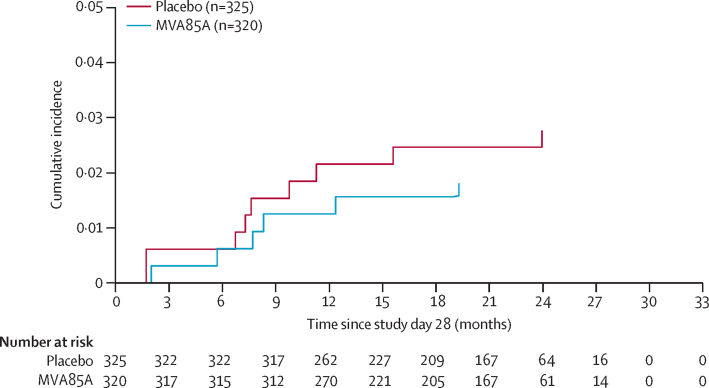
Cumulative incidence of diagnosis of tuberculosis endpoint 1 by treatment group Endpoint 1 was defined as a positive finding from culture or GeneXpert MTB/RIF assay.

In the per-protocol population, the overall number of tuberculosis cases and incidence during study follow-up of tuberculosis cases (endpoint 1) was six (2%) in the MVA85A group and nine (3%) in the placebo group, for a vaccine efficacy of 32·8% (95% CI −111·5 to 80·3; table 4). Figure 4 shows the Kaplan-Meier time-to-disease analysis for endpoint 1. Stratification by antiretroviral therapy status showed no significant difference between treatment groups. Eight of the 15 endpoint 1 cases were QFT positive at enrolment. No additional participants met endpoint 2 who did not already meet endpoint 1. Vaccine efficacy for endpoint 3 was 10·5% (−161·3 to 70.0). Disease incidence did not differ by site. Median time to diagnosis of endpoint 1 was 249 days in the MVA85A group and 236 days in the placebo group. 159 (50%) of 320 MVA85A recipients and 148 (46%) of 325 placebo recipients were investigated for tuberculosis during the study. The study was insufficiently powered to assess the efficacy of MVA85A for the prevention of tuberculosis disease in the subset of participants receiving antiretroviral therapy or isoniazid prophylaxis. The absence of efficacy also made it impossible to identify potential immunological correlates of protection from tuberculosis in participants vaccinated with MVA85A.

The number of QFT-negative participants who converted to QFT positive by the end of the study was 38 (20%) in the MVA85A group and 40 (23%) in the placebo group, for a vaccine efficacy of 11·7% (95% CI −41·3 to 44·9). QFT conversion did not differ by antiretroviral therapy status (data not shown), but it did differ by site. In Cape Town, 41 (31%) of 132 participants converted, whereas in Dakar, 37 (16%) of 227 converted (χ^2^ 10·89, p=0·001). Frequency of QFT reversion (participants who were positive at baseline and negative at end of study) was similar in the two treatment groups (17 [14%] of 124 for MVA85A and 27 [19%] of 139 for placebo; p=0·22), and did not differ by antiretroviral therapy status (data not shown). Tuberculin skin test conversion was not a prespecified endpoint and is not reported here, but will be the subject of further analysis.

## Discussion

This phase 2 trial in 650 adult participants infected with HIV-1 showed that MVA85A was well tolerated and immunogenic, with safety and immunogenicity profiles similar to those reported elsewhere for other populations in which this candidate vaccine has been assessed.^10^, ^11^, ^12^, ^13^, ^14^ However, we did not identify any significant efficacy against tuberculosis disease or *M tuberculosis* infection.

Both first and booster vaccination with MVA85A induced a significant increase in Ag85A-specific T cells. Responses did not differ by antiretroviral therapy status. A probable explanation for this finding is the high baseline median CD4 count (571 cells per mm^3^; table 1, appendix) in participants who had not received antiretroviral therapy. Unlike the previously reported infant efficacy trial of MVA85A,^10^ baseline ELISPOT responses were detected in this trial and were significantly higher in participants from Dakar than in those from Cape Town. This result might be due to greater exposure to environmental mycobacteria; and the finding is unlikely to be due to a technical issue because it was only recorded at this timepoint, and there was a robust quality control programme in place for these assays. Furthermore, the median response 7 days after vaccination in this trial exceeded that seen in the infant trial (337 *vs* 136 spots per million).^10^ Additionally, the functional phenotype of the dominant T-cell population in this trial was monofunctional by contrast with the infant trial, in which the dominant phenotype was polyfunctional.^10^ In both trials, the recorded response was insufficient to be associated with protection. It is not clear whether a quantitatively greater or a qualitatively different immune response is needed for protection. Alternative approaches, including the delivery of candidate vaccines direct to the respiratory mucosa, might be more potent routes of immunisation. For example, we have previously reported that delivery of MVA85A by aerosol to HIV-negative, BCG-vaccinated adults in the UK is well tolerated and induces potent mucosal and systemic immunity.^26^ Further assessment is needed before this route can be examined in countries with a high burden of tuberculosis. This approach, together with other strategies to improve the immunogenicity of MVA85A, are currently under investigation.

The recorded annual incidence of tuberculosis (endpoint 1) was substantial (1·43% across treatment groups) and did not differ between sites. However, this incidence was lower than previously reported in Cape Town.^27^ The numbers of participants receiving antiretroviral therapy was greater than originally envisaged, because of the increased availability of this therapy during the study period and the change in national and international guidelines on the provision of antiretroviral therapy. These factors, combined with the redesign of this study upon availability of the infant trial results,^10^ led to a reduction in statistical power to detect a difference in tuberculosis disease incidence between treatment groups, leading to wide CIs for our estimates of vaccine efficacy.

In this trial, the incidence of infection determined by QFT conversion was much higher than the incidence of tuberculosis disease, so CIs around the estimates of efficacy against infection are narrower. The overall recorded annual QFT conversion rate of about 12% meant that we had about 80% power to detect a vaccine efficacy of 50% against *M tuberculosis* infection. In view of the cost and complexity of human efficacy studies, there is now increased focus on infection as an endpoint rather than disease in proof-of-concept studies before progression to prevention-of-disease efficacy trials.^9^ However, this approach presupposes that the immune mechanisms needed to prevent infection and disease are similar. Our poor understanding of the biology underlying dynamic QFT conversion and reversion further complicates this shift in emphasis. The rate of QFT reversion was almost as high as the rate of conversion: whether this finding represents a true biological effect or technical variability in the assay cannot be determined from these data.

In this study, we have shown that high-quality, multicentre tuberculosis vaccine trials are possible in Africa, and have succeeded in the characterisation of the epidemiology of tuberculosis associated with HIV-1 in two African cities. Nevertheless, the disappointing finding with respect to vaccine efficacy for MVA85A suggests the need for standardised preclinical animal models that better represent human disease and an improved understanding of immune mechanisms of protection in human tuberculosis. Such advances would greatly enhance the ability to efficiently translate clinical research capacity into the development and deployment of an effective vaccine.

## Figures and Tables

**Table 1:** Demographic and baseline characteristics (safety analysis population)

		**Placebo (n=325)**	**MVA85A (n=324)**
Median age, years (range)		39·0 (22–41)	38·0 (21–49)
Women		255 (78%)	265 (82%)
Ethnic origin			
	Black	304 (94%)	302 (93%)
	Mixed	21 (6%)	22 (7%)
QFT test result			
	Positive	150 (46%)	135 (42%)
	Negative	173 (53%)	188 (58%)
	Indeterminate	2 (1%)	1 (<1%)
TST result			
	>5 mm	128 (39%)	124 (38%)
	≤5 mm	191 (59%)	190 (59%)
	Missing data	6 (2%)	10 (3%)
Latent tuberculosis infection		178 (55%)	164 (51%)
5–6 months IPT before enrolment		144 (44%)	133 (41%)
Receiving antiretroviral therapy		256 (79%)	257 (79%)
CD4 count (cells per mm^3^)			
	Participants not receiving antiretroviral therapy	564 (169·8)	571 (187·5)
	Participants receiving antiretroviral therapy	599 (199·6)	598 (220·7)
HIV viral load (copies per mL)			
	Participants not receiving antiretroviral therapy	41 371 (92 456·9)	62 168 (166 912·1)
	Participants receiving antiretroviral therapy	29 (27·1)	34 (63·7)

Data are n (%) or mean (SD), unless otherwise stated. QFT=QuantiFERON-TB Gold In-Tube. TST=tuberculin skin test. IPT=isoniazid preventive therapy.

**Table 2:** Overview of adverse events (safety analysis population)

	**Overall**			**Participants not receiving antiretroviral therapy**			**Participants receiving antiretroviral therapy**		
	Placebo (n=325)	MVA85A (n=324)	Difference (MVA85A minus placebo) (95% CI)	Placebo (n=69)	MVA85A (n=67)	Difference (MVA85A minus placebo) (95% CI)	Placebo (n=256)	MVA85A (n=257)	Difference (MVA85A minus placebo) (95% CI)
Any adverse event	312 (96·0%; 93·3–97·7)	321 (99·1%; 97·3–99·7)	3·1 (0·7 to 5·4)	67 (97·1%; 90·0–99·2)	66 (98·5%; 92·0–99·7)	1·4 (−3·5 to 6·3)	245 (95·7%; 92·5–97·6)	255 (99·2%; 97·2–99·8)	3·5 (0·8 to 6·2)
Solicited adverse event	235 (72·3%; 67·2–76·9)	288 (88·9%; 85·0–91·9)	16·6 (10·6 to 22·5)	50 (72·5%; 61·0–81·6)	63 (94·0%; 85·6–97·7)	21·6 (9·6 to 33·5)	185 (72·3%; 66·5–77·4)	225 (87·5%; 83·0–91·0)	15·3 (8·5 to 22·1)
Serious adverse event	17 (5·2%; 3·9–8·2)	17 (5·2%; 3·3–8·2)	0·02 (−3·4 to 3·4)	2 (2·9%; 0·8–10·0)	9 (13·4%; 7·2–23·6)	10·5 (1·5 to 19·6)	15 (5·9%; 3·6–9·4)	8 (3·1%; 1·6–6·0)	−2·7 (−6·3 to 0·8)
Related adverse event	307 (94·5%; 91·4–96·5)	318 (98·1%; 96·0–99·2)	3·7 (0·8 to 6·6)	66 (95·7%; 88·0–98·5)	66 (98·5%; 92·0–99·7)	2·9 (−2·8 to 8·5)	241 (94·1%; 90·6–96·4)	252 (98·1%; 95·5–99·1)	3·9 (0·6 to 7·2)
Severe adverse event	84 (25·8%; 21·4–30·9)	100 (30·9%; 26·1-36·1)	5·0 (−1·9 to 11·9)	15 (21·7%; 13·6–32·8)	22 (32·8%; 22·8–44·8)	11·1 (−3·8 to 26)	69 (27·0%; 21·7–32·9)	78 (30·4%; 25·1–36·2)	3·4 (−4·4 to 11·2)

Data are n (%; 95% CI), unless otherwise stated. Serious adverse events were coded with Medical Dictionary for Regulatory Activities version 14.0. Patients with multiple events in each category are counted only once in each category.

**Table 3:** Total intracellular cytokine response, presented as frequency of CD4 T cells and CD8 T cells producing specific cytokines

	**MVA85A (n=28)**				**MVA85A timepoint comparisons (p values)**				**Placebo (n=29)**			
	Day 0 (vaccination 1)	Day 7 (vaccination 1)	Day 0 (vaccination 2)	Day 7 (vaccination 2)	Day 0 (vaccination 1) *vs* day 7 (vaccination 1)	Day 0 (vaccination 1) *vs* day 0 (vaccination 2)	Day 0 (vaccination 1) *vs* day 7 (vaccination 2)	Day 0 (vaccination 2) *vs* day 7 (vaccination 2)	Day 0 (vaccination 1)	Day 7 (vaccination 1)	Day 0 (vaccination 2)	Day 7 (vaccination 2)
CD4 IFNγ	0·01 (0–0·07)	0·1 (0–1·12)	0·03 (0–0·28)	0·11 (0·02–0·82)	<0·0001	0·0015	<0·0001	<0·0001	0·02 (0–0·12)	0·01 (0–0·08)	0 (0–0·08)	0·01 (0–0·18)
CD4 TNFα	0·02 (0–0·12)	0·11 (0–0·53)	0·05 (0–0·57)	0·11 (0–0·46)	<0·0001	0·0403	<0·0001	<0·0001	0·02 (0–0·15)	0·02 (0-0·14)	0·02 (0–0·11)	0·02 (0–0·23)
CD4 IL-2	0·021 (0–0·11)	0·07 (0–0·68)	0·04 (0–0·27)	0·1 (0·03–0·44)	<0·0001	0·0421	<0·0001	<0·0001	0·02 (0–0·08)	0·017 (0–0·08)	0·02 (0–0·09)	0·018 (0–0·06)
CD4 IL-17	0·09 (0·01–0·28)	0·12 (0·03–0·27)	0·09 (0–0·37)	0·1 (0·03–0·23)	0·0946	0·5425	0·4047	0·2843	0·07 (0–0·27)	0·06 (0·02–0·27)	0·08 (0·01–0·26)	0·078 (0–0·25)
CD8 IFNγ	0 (0–0·21)	0·02 (0–0·94)	0 (0–0·58)	0·01 (0–0·3)	0·0101	0·5499	0·2264	0·2897	0 (0–0·35)	0 (0–0·19)	0 (0–0·33)	0 (0–0·24)
CD8 TNFα	0 (0–0·28)	0 (0–0·24)	0 (0–0·48)	0 (0–0·05)	0·4513	0·7615	0·7337	0·3953	0 (0–0·09)	0 (0–0·38)	0 (0–0·2)	0 (0–0·13)

Data are median (minimum to maximum) of total cytokines at each of the study timepoints, unless otherwise stated. Population is the immunology substudy (the first 70 participants), of which complete data were available for 57 participants. Statistical comparison of total cytokine responses in MVA85A study group used Wilcoxon matched-pairs signed-rank test. IL=interleukin. IFNγ=interferon γ. TNFα=tumour necrosis factor α.

**Table 4:** Primary and secondary efficacy results (per-protocol population)

	**Overall**			**Participants not receiving antiretroviral therapy**			**Participants receiving antiretroviral therapy**		
	Placebo	MVA85A	Vaccine efficacy (95% CI)	Placebo	MVA85A	Vaccine efficacy (95% CI)	Placebo	MVA85A	Vaccine efficacy (95% CI)
Disease endpoint 1 (primary efficacy endpoint)	9/325 (2·8%)	6/320 (1·9%)	32·8%(−111·5 to 80·3)	1/69 (1·4%)	2/65 (3·1%)	−114·1%(−12 528·3 to 88·9)	8/256 (3·1%)	4/255 (1·6%)	50·3%(−85·4 to 89·1)
Disease endpoint 3	9/325 (2·8%)	8/320 (2·5%)	10·5%(−161·3 to 70·0)	1/69 (1·4%)	3/65 (4·6%)	−224·7%(−16 947·7 to 73·9)	8/256 (3·1%)	5/255 (2·0%)	38·2%(−114·1 to 84·1)
QFT positive conversion	40/173 (23·1%)	38/186 (20·4%)	11·7%(−41·3 to 44·9)	11/36 (30·6%)	6/38 (15·8%)	44·2%(−64·8 to 83·0)	29/137 (21·2%)	32/148 (21·6%)	−0·1%(−71·5 to 41·4)

Data are n/N (%), unless otherwise stated. Disease endpoint 1 was defined as culture or GeneXpert MTB/RIF positivity; disease endpoint 2 included endpoint 1 and a composite clinical endpoint; and disease endpoint 3 was commencement on anti-tubercular chemotherapy. No additional participants met endpoint 2 who did not already meet endpoint 1. QFT=QuantiFERON-TB Gold In-Tube.
