# Supplementary material for: Safety, immunogenicity, and efficacy of the candidate tuberculosis vaccine MVA85A in healthy adults infected with HIV-1: a randomised, placebo-controlled, phase 2 trial
Source: Lancet Respir Med. 2015 Mar;3(3):190–200. doi: 10.1016/S2213-2600(15)00037-5 (PMC4648060; doi:10.1016/S2213-2600(15)00037-5)
Supplement: Supplementary appendix [file mmc1.pdf]

THE LANCET Respiratory Medicine

Supplementary appendix

This appendix formed part of the original submission and has been peer reviewed. We post it as supplied by the authors.

Supplement to: Ndiaye BP, Thienemann F, Ota M, et al. Safety, immunogenicity, and efficacy of the candidate tuberculosis vaccine MVA85A in healthy adults infected with HIV-1: a randomised, placebo-controlled, phase 2 trial. *Lancet Respir Med* 2015; published online Feb 26. http://dx.doi.org/10.1016/S2213-2600(15)00037-5.

SUPPLEMENTARY APPENDIX

Supplement to: Safety and efficacy of the candidate tuberculosis vaccine MVA85A in healthy HIV-infected adults: A randomized, placebo-controlled phase 2b trial

TABLE OF CONTENTS

Contributors

MVA85A 030 trial investigators page 1

Acknowledgements page 2

Supplementary results

Table S1: Demographics and baseline characteristics of safety population page 4

Table S2: Incidence of serious adverse events page 7

CONTRIBUTORS

MVA85A 030 trial investigators

Aeras

Margaret Anne Snowden, J Bruce McClain, Barbara Shepherd

Centre de Traitement Ambulatoire, Centre Hospitalier Universitaire de Fann, Senegal

Ndèye Fatou Ngom, Ibrahima Sow, Mamadou Gueye,

Centre de Recherche Clinique et de Formation, Centre Hospitalier Universitaire de Fann, Senegal

Papa Salif SOW

Clinical Infectious Diseases Research Initiative, University of Cape Town, South Africa

Yolande Harley, Thelma Leopeng, Phila Mawu, Relebohile Tsekela, Andiswa Beatrice Vasi, Nobantu Jack, Ida Oliphant, Patricia Rini, Fadheela Patel, Kerryn Matthews, Melina Carr, Nonzwakazi Bangani, Zeinonisaa Latief, Patrick Hamadi, Raylene Titus, Antoneta Mashinyira, Nomvula Makade, Amanda Jackson, Lorraine Swanepoel, Nashreen Omar-Davies, Derval Reidy, Abongile Makuluma, Marianne Skreden, Nolitha Qondisa Qina

Laboratoire de Bacteriolgie-Virologie, Centre Hospitalier Universitaire Le Dantec, Senegal

Modou Seck, Allé Baba Dieng, Awa Ba, Abdou Padane, Khady Joséphine Badiane, Ndèye Salimata Santos, Ndèye Coumba Touré, Halimatou Diop, Ousmane Diouf, Mouhamedou Ndiaye, Aminata Dia, Tidiane Siby, Aliou Niang, Mamadou Diarrha Bèye, Papa Ibrahima Ndiaye, Sada Diallo, Ousseynou Ndiaye, Aliou Sène, Ameth Boly Niang, Awa Ndiaye, Mame Yacine Fall, Marema Fall

The Jenner Institute, University of Oxford

Sam Vermaak, Alison Lawrie, Kristin Griffiths

Acknowledgements

Site B Clinic Khayelitsha

All clinical and managerial staff at the site, Ubuntu Clinic, Day Hospital, Maternal Obstetric Unit and Casualty, particularly Dr Shaheed Mathee, Bekekile Kwaza, Brian Basini, Nonceba Gobe, Betty Kondo, Thembisa Ngcuka, Portia Ngondeka, Robyn Louw and Lungiswa Mshudulu

Laboratoire de Bacteriolgie-Virologie, Centre Hospitalier Universitaire Le Dantec, Senegal

All clinical and managerial staff, particularly Astou Fall Cisse and Daouda Gueye

Centre de Traitement Ambulatoire, Centre Hospitalier Universitaire de Fann, Senegal

All clinical and managerial staff, particularly Astou Diagne and Djibril Baal

Centre de Recherche Clinique et de Formation, Centre Hospitalier Universitaire de Fann, Senegal

All clinical and managerial staff

The Jenner Institute, University of Oxford

Lisa Stockdale

Emergent Biosolutions Inc

Stephen Lockhart

Oxford Emergent Tuberculosis Consortium

Jacqueline Shea

Aeras

Thomas Evans, Ann Ginsberg, Jasur Ishmukhamedov

Data Monitoring Committee

Andreas Diacon (chair), Prakash Jeena, Neil Cameron, James McIntyre, Alison Elliott, Benjamin (Gil) Price, James Balsley

SUPPLEMENTARY RESULTS

Table S1: Demographics and baseline characteristics of safety analysis population

		Overall (N=649)		Cape Town (N=292)		Dakar (N=357)	
		**Placebo (N=325)**	**MVA85A (N=324)**	**Placebo (N=146)**	**MVA85A (N=146)**	**Placebo (N=179)**	**MVA85A (N=178)**
Age; n (range)		39.0 (22-41)	38.0 (21-49)	36.0 (22-49)	36.0 (22-49)	40.0 (23-49)	39.0 (21-49)
Female; n (%)		255 (78.5)	265 (81.8)	127 (87.0)	127 (87.0)	128 (71.5)	138 (77.5)
Race; n (%)	Black	304 (93.5)	302 (93.2)	146 (100.0)	146 (100.0)	158 (88.3)	156 (87.6)
	Mixed racial ancestry	21 (6.5)	22 (6.8)	0 (0.0)	0 (0.0)	21 (11.7)	22 (12.4)
QFT test result; n (%)	Positive	150 (46.2)	135 (41.7)	79 (54.1)	78 (53.4)	71 (39.7)	57 (32.0)
	Negative	173 (53.2)	188 (58.0)	66 (45.2)	68 (46.6)	107 (59.8)	120 (67.4)

	Indeterminate	2 (0.6)	1 (0.3)	1 (0.7)	0 (0.0)	1 (0.6)	1 (0.6)
TST result; n (%)	>5mm	128 (39.4)	124 (38.3)	54 (37.0)	60 (41.1)	74 (41.3)	64 (36.0)
	≤5mm	191 (58.8)	190 (58.6)	86 (58.9)	76 (52.1)	105 (58.7)	114 (64.0)
	Missing	6 (1.8)	10 (3.1)	6 (4.1)	10 (6.8)	0 (0.0)	0 (0.0)
Latently infected*; n (%)		178 (54.8)	164 (50.6)	87 (59.6)	89 (61.0)	91 (50.8)	75 (42.1)
5-6 months IPT prior to enrolment; n (%)		144 (44.3)	133 (41.0)	68 (46.6)	69 (47.3)	76 (42.5)	64 (36.0)
Receiving ART; n (%)		256 (78.8)	257 (79.3)	109 (74.7)	110 (75.3)	147 (82.1)	147 (82.6)
CD4 (cells/mm^3); mean (SD)	ART-	564 (169.8)	571 (187.5)	572 (176.5)	578 (202.7)	554 (164.0)	562 (171.1)
	ART+	599 (199.6)	598 (220.7)	637 (192.3)	632 (251.2)	571 (200.8)	572 (191.7)
HIV viral load (copies/ml); mean (SD)	ART-	41371 (92456.9)	62168 (166912.1)	20944 (40176.7)	77807 (215689.5)	65753 (126590.7)	43923 (79377.5)

ART+	29 (27.1)	34 (63.7)	25 (0.0)	25 (3.3)	32 (35.9)	40 (84.2)

QFT: QuantiFERON® Gold In-Tube; IPT: isoniazid preventive therapy; ART: antiretroviral therapy

* TST (PPD skin test) >5mm or QFT positive

Table S2: Incidence of serious adverse events (safety analysis population)

	Placebo			MVA85A		
**Primary System Organ Class**<br>**Preferred Term**	**ART-**<br>**(N=69)**<br>**n (%)**	**ART+**<br>**(N=256)**<br>**n (%)**	**Total**<br>**(N=325)**<br>**n (%)**	**ART-**<br>**(N=67)**<br>**n (%)**	**ART+**<br>**(N=257)**<br>**n (%)**	**Total**<br>**(N=324)**<br>**n (%)**
**Patients with at least one SAE**	2 (2.9)	15 (5.9)	17 (5.2)	9 (13.4)	8 (3.1)	17 (5.2)
**Infections and infestations**	1 (1.4)	7 (2.7)	8 (2.5)	1 (1.5)	4 (1.6)	5 (1.5)
Bronchopneumonia	0 (0.0)	0 (0.0)	0 (0.0)	0 (0.0)	1 (0.4)	1 (0.3)
Erysipelas	0 (0.0)	1 (0.4)	1 (0.3)	0 (0.0)	0 (0.0)	0 (0.0)
Eye infection syphilitic	0 (0.0)	0 (0.0)	0 (0.0)	0 (0.0)	1 (0.4)	1 (0.3)
Localised infection	0 (0.0)	1 (0.4)	1 (0.3)	0 (0.0)	0 (0.0)	0 (0.0)
Malaria	1 (1.4)	1 (0.4)	2 (0.6)	0 (0.0)	0 (0.0)	0 (0.0)
Meningitis tuberculous	0 (0.0)	0 (0.0)	0 (0.0)	1 (1.5)	0 (0.0)	1 (0.3)
Meningitis viral	0 (0.0)	1 (0.4)	1 (0.3)	0 (0.0)	0 (0.0)	0 (0.0)
Pneumonia	0 (0.0)	2 (0.8)	2 (0.6)	0 (0.0)	0 (0.0)	0 (0.0)
Pulmonary tuberculosis	0 (0.0)	1 (0.4)	1 (0.3)	0 (0.0)	1 (0.4)	1 (0.3)
Septic shock	0 (0.0)	0 (0.0)	0 (0.0)	0 (0.0)	1 (0.4)	1 (0.3)
**Psychiatric disorders**	0 (0.0)	1 (0.4)	1 (0.3)	1 (1.5)	0 (0.0)	1 (0.3)
Anxiety disorder	0 (0.0)	1 (0.4)	1 (0.3)	0 (0.0)	0 (0.0)	0 (0.0)

Suicide attempt	0 (0.0)	0 (0.0)	0 (0.0)	1 (1.5)	0 (0.0)	1 (0.3)
**Nervous system disorders**	0 (0.0)	2 (0.8)	2 (0.6)	2 (3.0)	1 (0.4)	3 (0.9)
Convulsion	0 (0.0)	0 (0.0)	0 (0.0)	1 (1.5)	0 (0.0)	1 (0.3)
Hemiplegia	0 (0.0)	1 (0.4)	1 (0.3)	0 (0.0)	0 (0.0)	0 (0.0)
Meningorrhagia	0 (0.0)	0 (0.0)	0 (0.0)	0 (0.0)	1 (0.4)	1 (0.3)
Transient ischaemic attack	0 (0.0)	1 (0.4)	1 (0.3)	0 (0.0)	0 (0.0)	0 (0.0)
Seventh nerve paralysis	0 (0.0)	0 (0.0)	0 (0.0)	1 (1.5)	0 (0.0)	1 (0.3)
**Cardiac disorders**	0 (0.0)	0 (0.0)	0 (0.0)	1 (1.5)	0 (0.0)	1 (0.3)
Atrioventricular block complete	0 (0.0)	0 (0.0)	0 (0.0)	1 (1.5)	0 (0.0)	1 (0.3)
**Vascular disorders**	0 (0.0)	1 (0.4)	1 (0.3)	0 (0.0)	0 (0.0)	0 (0.0)
Deep vein thrombosis	0 (0.0)	1 (0.4)	1 (0.3)	0 (0.0)	0 (0.0)	0 (0.0)
**Respiratory, thoracic and mediastinal disorders**	0 (0.0)	2 (0.8)	2 (0.6)	0 (0.0)	1 (0.4)	1 (0.3)
Epistaxis	0 (0.0)	1 (0.4)	1 (0.3)	0 (0.0)	0 (0.0)	0 (0.0)
Pleural effusion	0 (0.0)	1 (0.4)	1 (0.3)	0 (0.0)	0 (0.0)	0 (0.0)
Pulmonary oedema	0 (0.0)	0 (0.0)	0 (0.0)	0 (0.0)	1 (0.4)	1 (0.3)
**Gastrointestinal disorders**	0 (0.0)	1 (0.4)	1 (0.3)	0 (0.0)	1 (0.4)	1 (0.3)
Gastritis	0 (0.0)	0 (0.0)	0 (0.0)	0 (0.0)	1 (0.4)	1 (0.3)
Haematemesis	0 (0.0)	1 (0.4)	1 (0.3)	0 (0.0)	0 (0.0)	0 (0.0)

**Pregnancy, puerperium and perinatal conditions**	0 (0.0)	1 (0.4)	1 (0.3)	3 (4.5)	1 (0.4)	4 (1.2)
Abortion spontaneous	0 (0.0)	1 (0.4)	1 (0.3)	3 (4.5)	0 (0.0)	3 (0.9)
Intra-uterine death	0 (0.0)	0 (0.0)	0 (0.0)	0 (0.0)	1 (0.4)	1 (0.3)
**Congenital, familial and genetic disorders**	0 (0.0)	0 (0.0)	0 (0.0)	1 (1.5)	0 (0.0)	1 (0.3)
Congenital anomaly	0 (0.0)	0 (0.0)	0 (0.0)	1 (1.5)	0 (0.0)	1 (0.3)
**General disorders and administration site conditions**	1 (1.4)	1 (0.4)	2 (0.6)	0 (0.0)	0 (0.0)	0 (0.0)
Death	0 (0.0)	1 (0.4)	1 (0.3)	0 (0.0)	0 (0.0)	0 (0.0)
Death neonatal	1 (1.4)	0 (0.0)	1 (0.3)	0 (0.0)	0 (0.0)	0 (0.0)

SAEs coded using MedDRA Version 14.0. Patients with multiple events in each category are counted only once in each category

ART: Antiretroviral therapy; SAE: serious adverse event; MedDRA: Medical Dictionary for Regulatory Activities

A Phase II, Proof of Concept, Randomized, Double-blind, Placebo-controlled Study to Evaluate the Protective Efficacy Against TB Disease, Safety, and Immunogenicity of MVA85A/AERAS-485 in Healthy, HIV-infected Adults

Investigational Product: **MVA85A/AERAS-485**

Aeras Protocol Number: **C-030-485 (Oxford TB021)**

Study Monitor:

Triclinium Clinical Trial Project Management Ltd
135 West Street (4th floor)
Sandown 2196
South Africa
Phone: +27 (11) 883-0205/06/07
Fax: +27 (11) 784-2818

Sponsor:

Aeras
1405 Research Boulevard, Suite 300
Rockville, Maryland 20850 USA
Phone: 001-301-547-2900
Fax: 001-301-547-2901

Sponsor's Authorized Representative:

Ann Ginsberg, MD, PhD
Chief Medical Officer

Principal Investigator Agreement:

I, the undersigned, have reviewed this protocol and agree to conduct this protocol in accordance with Good Clinical Practices (ICH-GCP), the ethical principles set forth in the Declaration of Helsinki, and with local regulatory requirements.

Signature Date

Printed Name

TABLE OF CONTENTS

STUDY ABSTRACT 7

1 INTRODUCTION 11
1.1 Background 11
1.2 Description of MVA85A/AERAS-485 11
1.3 Nonclinical Experience with MVA85A/AERAS-485 12
1.4 Clinical Experience with MVA85A/AERAS-485 12
1.5 Rationale for Study 14

2 STUDY OBJECTIVES AND DESIGN 14
2.1 Objectives 14
2.2 Design 15

3 STUDY PROCEDURES 16
3.1 Schedule of Subject Evaluations 16
3.2 Subject Selection 18
3.2.1 Recruitment and Informed Consent 18
3.2.2 Screening 19
3.2.3 Inclusion Criteria 19
3.2.4 Exclusion Criteria 20
3.2.5 Screening Clinical Assessments and Laboratory Tests 21
3.3 Study Randomization 21
3.4 Blinding 21
3.4.1 Unblinding for Clinical Emergencies 22
3.5 Study Vaccine Administration 22
3.6 Study Evaluations 23
3.6.1 Pre-vaccination and Post-Vaccination Monitoring of Subjects 23
3.6.2 Clinical Assessments and Laboratory Tests 23
3.6.2.1 Abnormal Clinical Assessments and Laboratory Tests 24
3.6.3 Evaluation of Tuberculosis/Mortality 24
3.6.4 Immunology Laboratory Evaluations 26
3.6.4.1 Real Time Evaluation of Vaccine Immunogenicity 26
3.6.4.2 Immune Correlate Samples 27
3.6.5 Concomitant Medications 27
3.6.6 Subject Follow-up and Contact 28
3.6.7 Loss to Follow-up 29

4 STUDY VACCINES 29
4.1 Supplies 29
4.2 Accountability 29
4.3 Receipt and Storage 30
4.4 Vaccine/Placebo Preparation 30
4.5 Disposal of Unused Supplies 31

5 SAFETY ... 31
5.1 Responsibilities for Ensuring the Safety of Trial Subjects ... 31
5.1.1 Principal Investigator ... 31
5.1.1.1 Pausing Rules for the Principal Investigator ... 31
5.1.2 Study Sponsor ... 32
5.1.3 Local Medical Monitor ... 32
5.1.3.1 Pausing Rules for the Local Medical Monitor ... 32
5.1.4 Global Medical Monitor ... 32
5.1.5 Data Monitoring Committee ... 33
5.1.6 Institutional Review Boards and Ethics Committees ... 33
5.1.7 National Regulatory Authority ... 33
5.2 Safety Surveillance During the Study ... 34
5.3 Definition of Adverse Event ... 34
5.4 Assessing Severity ... 35
5.5 Assessing Causal Relationship (Relatedness) ... 36
5.6 Definition of Adverse Reaction ... 37
5.7 Solicited Adverse Events and Injection Site Reactions ... 37
5.8 Subject Diary and Daily Temperature Monitoring ... 38
5.9 Assessing "Seriousness" and Serious Adverse Events ... 38
5.10 Assessing Expectedness ... 39
5.11 Definition of Suspected Unexpected Serious Adverse Reaction (SUSAR) ... 39
5.12 Reporting of Serious Adverse Events ... 39
5.13 Other Events Requiring Immediate Reporting ... 40
5.14 Adverse Event Treatment, Follow-up, and Outcome ... 41
5.15 Follow-up of Subjects Who Become Pregnant ... 41

6 STOPPING RULES ... 42

7 STATISTICAL CONSIDERATIONS ... 43
7.1 Subject Populations ... 43
7.2 Demographics and Protocol Compliance ... 43
7.3 Efficacy Analyses ... 44
7.3.1 Case Definition of Tuberculosis ... 44
7.3.2 Efficacy of MVA85A/AERAS-485 ... 45
7.3.3 QuantiFERON Conversion Rate ... 46
7.4 Safety Analyses ... 46
7.4.1 Adverse Events ... 46
7.4.2 Clinical Laboratory and Vital Sign Parameters ... 47
7.4.3 CD4+ Lymphocyte Count ... 47
7.4.4 HIV-1 Viral Load ... 47
7.5 Immunogenicity and Correlates of Protection ... 47
7.5.1 Immune Response Determined by ELISPOT Assay ... 47
7.5.2 Immune Response Determined by Intracellular Cytokine Staining Assay ... 48
7.5.3 Correlates of Protection ... 48

7.5.4 Other Immunology Analyses 48
7.5.5 Exploratory Immunology Analyses 48
7.5.6 Tuberculin PPD Skin Test 48
7.6 Sample Size Considerations 49
7.7 Interim Immunogenicity Review 49
7.8 Safety Reviews 49
7.9 Final Study Report 50
7.10 Computer Methods 50

8 DATA COLLECTION, MONITORING, AND RECORD RETENTION 50

9 HUMAN SUBJECTS 50
9.1 Ethics and Regulatory Considerations 50
9.2 Institutional Review Board or Independent Ethics Committee 51
9.3 Informed Consent 51

10 STUDY COMPLETION 52

11 PUBLICATIONS 53

12 CHANGES IN THE PROTOCOL 53
12.1 Changes from Version 1.0 to Version 2.0 53
12.2 Changes from Version 2.0 to Version 3.0 56
12.3 Changes from Version 3.0 to Version 4.0 59
12.4 Changes from Version 4.0 to Version 5.0 61
12.5 Changes from Version 5.0 to Version 6.0 62

13 REFERENCES 66

APPENDIX A Detailed Description of Study Visits 67

APPENDIX B SAE Reporting Scheme 71

APPENDIX C Toxicity Table 72

APPENDIX D Phlebotomy Volumes 75

APPENDIX E Subject Diary (Template) 76

LIST OF ABBREVIATIONS

AE	Adverse event(s)
ALP	Alkaline Phosphatase
ALT	Alanine aminotransferase
ART	Anti-retroviral therapy
AST	Aspartate aminotransferase
AUC	Area under the curve
BCG	Bacillus Calmette-Guérin
CBC	Complete blood count
CPT	Cell Preparation Tube
CRF	Case Report Form(s)
CTA	Clinical Trials Application
dL	Deciliter
DMC	Data Monitoring Committee
eCRF	electronic Case Report Form
ER	Emergency room
FDA	United States Food and Drug Administration
FP	Fowl pox
g	Gram
GCP	Good clinical practices
GGT	Gamma-glutamyl-transferase
GMO	Genetically modified organism
GMP	Good manufacturing practices
HIV	Human immunodeficiency virus
ICH	International Conference on Harmonization of Technical Requirements for Registration of Pharmaceuticals for Human Use
ICS	Intracellular cytokine staining
ID	Intradermal
IEC	Independent Ethics Committee
IFN-γ	Interferon gamma
IM	Intramuscular
IND	Investigational New Drug Application
IRB	Institutional Review Board
ITT	Intent-to-treat (population)
IVRS	Interactive voice response system
kDa	Kilodalton
L	Liter
MedDRA	Medical Dictionary for Regulatory Activities
µmol	Micromole(s)
mL	Milliliter(s)
mm	Millimeter(s)
mmol	Millimole(s)
MOTT	Mycobacterium other than tuberculosis
Mtb	*Mycobacterium tuberculosis*
MVA	Modified vaccinia virus Ankara

PBMC	Peripheral blood mononuclear cell(s)
pfu	plaque-forming unit
PMTCT	Prevention of mother-to-child transmission
PP	Per-protocol (population)
PPD	Purified protein derivative
QFN	QuantiFERON®
QFT-G	QuantiFERON®-TB Gold In-Tube test, Cellestis Ltd.
SAE	Serious adverse event(s)
SolAE	Solicited adverse event(s)
SOP	Standard operating procedure
SST	Serum separator tube
SUSAR	Suspected unexpected serious adverse reaction(s)
TB	Tuberculosis
TNF-α	Tumor necrosis factor alpha
ULN	Upper limit of normal
WBC	White blood cell

STUDY ABSTRACT

TITLE:

A Phase II, Proof-of-concept, Randomized, Double-blind, Placebo-controlled Study to Evaluate the Protective Efficacy Against TB Disease, Safety, and Immunogenicity of MVA85A/AERAS-485 in Healthy, HIV-infected Adults

RATIONALE:

The available live tuberculosis vaccine, BCG, provides incomplete protection against pulmonary tuberculosis. Due to natural inhibition of a second dose, a BCG revaccination does not provide much additional protection. MVA85A/AERAS-485 presents a tuberculosis antigen in the setting of a live but non-replicating virus vaccine to increase T-cell immunity and thus protection against tuberculosis. MVA85A/AERAS-485 has been administered in clinical trials to over 2000 subjects, including 80 adult subjects who were HIV-positive, without any vaccine-related serious adverse events (including no clinically significant effect on either CD4 count or HIV viral load) and shows evidence of immunogenicity. Following licensure, MVA85A/AERAS-485 will be made available in areas of high HIV prevalence. It is therefore important to study the efficacy and safety of the vaccine in HIV-positive individuals. This study will evaluate the safety of MVA85A/AERAS-485 in HIV-positive adults with baseline CD4 counts above 350 cells/mm^3 if not receiving ART or above 300 cells/mm^3 if receiving ART. In addition, the protective efficacy against TB disease and the immunogenicity of MVA85A/AERAS-485 will be evaluated.

OBJECTIVES:

Primary Objective

The primary objective of this study is to evaluate the safety of MVA85A/AERAS-485 compared to placebo.

Secondary Objectives

The secondary objectives of this study are:

1. To evaluate the efficacy of MVA85A/AERAS-485 in the prevention of TB disease compared to control subjects who receive placebo in HIV-infected, African adult subjects without active TB disease.
2. To evaluate CD4+ lymphocyte counts and HIV-1 viral load before and after administration of MVA85A/AERAS-485 compared to placebo.
3. To evaluate the efficacy of MVA85A/AERAS-485 in the prevention of TB disease in subjects receiving ART at baseline compared to subjects receiving ART at baseline but who receive placebo.
4. To evaluate the efficacy of MVA85A/AERAS-485 in the prevention of TB disease in subjects who received isoniazid preventive therapy compared to control subjects who also received isoniazid preventive therapy but who receive placebo.
5. To evaluate the immunogenicity of MVA85A/AERAS-485 compared to placebo as described by the *ex vivo* IFN-γ ELISPOT assay.

6. To evaluate the immunogenicity of MVA85A/AERAS-485 compared to placebo as described by flow cytometric intracellular cytokine staining of CD4+ and CD8+ T cells after stimulation with a peptide pool of mycobacterial antigens.
7. To identify potential immunological correlates of protection from tuberculosis in subjects vaccinated with MVA85A/AERAS-485.
8. To evaluate the QuantiFERON (QFN) conversion rate at final study assessment in MVA85A/AERAS-485 recipients compared to control subjects without a diagnosis of tuberculosis during the trial.

DESIGN:

This Phase II multi-country trial will be conducted as a randomized, double-blind, placebo-controlled, proof-of-concept trial in 650 HIV-positive adults with no evidence of active TB disease. Subjects will be stratified at the time of randomization by whether or not they are currently receiving antiretroviral therapy (ART) and then randomized 1:1 to receive either MVA85A/AERAS-485 at 1 x 10^8pfu or placebo (Candin®), as shown in Table 0-1. Randomization of each group will be capped so that at least 50% of the subjects randomized will be receiving ART at randomization. Subjects will receive an intradermal injection of MVA85A/AERAS-485 or placebo on Study Day 0, followed 6-9 months later by a booster injection of MVA85A/AERAS-485 or placebo. The minimum follow-up period for each subject will be 6 months after their last vaccination, during which subjects will be followed for safety, clinical signs and symptoms of TB, and immunogenicity. Subjects will continue to be followed every 3 months until the last subject enrolled has been followed for 6 months after their last vaccination.

Table 0-1 Treatment Groups

Treatment Group	Number of Subjects	Dosing Regimen
MVA85A/AERAS-485 1 x 10^8pfu	325	Study Day 0 (prime), SD168-252 (boost)
Placebo	325	Study Day 0 (prime), SD168-252 (boost)

ANALYSIS OF PRIMARY OBJECTIVE

The primary variable for evaluation of the safety of MVA85A/AERAS-485 will be the number and percentage of solicited and unsolicited adverse events (including serious adverse events [SAEs]) recorded over the post-vaccination follow-up period for all subjects in the safety population. The number (percentage) of subjects with adverse events will be summarized by MedDRA system organ class (SOC) and preferred term (PT). The proportion of subjects in the control and MVA85A/AERAS-485 treatment groups with at least one adverse event will be compared using Fisher's Exact test; two-sided exact 95% confidence intervals will also be presented for proportions of individual adverse event preferred terms, within treatment group. Summaries by ART status will also be presented. Separate summaries will also present the number (percentage) of subjects with adverse events by severity and by relationship to study vaccine; each subject will be counted once per preferred term at the greatest severity or most related state recorded for that term.

ANALYSIS OF SECONDARY OBJECTIVES

Efficacy of MVA85A/AERAS-485

For the protocol-specified secondary objective of TB disease, subjects will be evaluated for the development of TB disease throughout the study utilizing TB Case Definition Endpoint #1 as the primary disease endpoint assessment. Less stringent TB case definitions, Endpoint #2 and Endpoint #3, will also be evaluated with no adjustment for multiplicity performed; it is acknowledged that the overall Type I error probability is not controlled in the evaluation of these endpoints.

Efficacy of MVA85A/AERAS 485 in the prevention of TB disease will be examined for all subjects in the per protocol (PP) population. The incidence of TB disease meeting Endpoint #1, 2, and 3 will be calculated as the number of new cases of TB disease with a date of diagnosis from 28 days post-Study Day 0 vaccination through the end of study follow-up, as defined for each subject. Efficacy of MVA85A/AERAS 485 in the prevention of TB disease will be examined for all subjects in the PP population receiving ART at baseline compared to subjects receiving ART at baseline but who receive placebo (secondary objective #3), as described above. In addition, the efficacy of MVA85A/AERAS 485 in the prevention of TB disease will be examined for all subjects in the PP population receiving MVA85A/AERAS-485 and isoniazid preventative therapy at any time during the study following Study Day 0 vaccination compared to subjects receive placebo (secondary objective #4).

CD4+ Lymphocyte Count

Median or geometric mean CD4+ lymphocyte counts and associated two-sided 95% confidence interval will be summarized by ART status at baseline, study site, treatment group, and time point. Summaries will include change in CD4+ lymphocyte count from pre-vaccination to each post-vaccination time point. Difference in CD4+ count between treatment groups over the course of the study will be evaluated using an area under the curve (AUC) and associated two-sided 95% CIs.

HIV-1 Viral Load

HIV-1 viral load (copies/mL) will be summarized using medians and associated 95% confidence interval by ART status at baseline, study site, and treatment group, at each available time point. Summaries will include change from pre-vaccination to post-vaccination time points.

T Cell Immune Response and Correlates of Protection

The immune response of MVA85A/AERAS-485 based on the *ex vivo* IFN-γ ELISPOT and intracellular cytokine staining (ICS) assay will be described for a subset of subjects tested. These assays will be used to determine vaccine immunogenicity on the same 5% of subjects (70 in total, with a minimum of 30 from each site). Samples will be taken from these 70 subjects at the following time points after each vaccination: 0 (baseline day of vaccination), 7 days, and 28 days post vaccination. Response will be presented at each post-immunization time point, by ART status at baseline, and by treatment group.

QuantiFERON Conversion Rate

QuantiFERON®-TB Gold In-Tube test results through the end of the study will be summarized by number (percentage) of positive responses by ART status at baseline and treatment group. Cross-tabulation of the number of subjects with a negative baseline QuantiFERON with subsequent conversion to a positive result post-vaccination will be summarized by ART status and treatment group.

1 INTRODUCTION

1.1 Background

The only currently available tuberculosis vaccine, Bacillus Calmette-Guérin (BCG), is an attenuated live vaccine derived from *Mycobacterium bovis* and is estimated to reduce the risk of tuberculosis in children by about 80% (1). While effective chemotherapy is available, the uncertainties in diagnosis within the affected populations and the expense and toxicity of chemotherapy permit continued morbidity and death. An improved vaccination would have a positive effect on the health of many people.

Heterologous 'prime–boost immunization' strategies involve administration of two different vaccines that deliver the same antigen weeks or months apart. This approach has been demonstrated to elicit higher cellular immunity than a single BCG vaccination in several animal disease models (2, 3). Given the protective efficacy of BCG in childhood, BCG would ideally be the priming immunization in such a prime–boost strategy.

The University of Oxford has developed a vaccination strategy with BCG as the priming vaccination and MVA85A/AERAS-485 as the boost. MVA85A/AERAS-485 is a recombinant MVA expressing the *Mycobacterium tuberculosis* antigen Ag85A. MVA is modified vaccinia virus Ankara, a highly attenuated strain of vaccinia virus which does not replicate in mammalian cells. Ag85A is an immunodominant secreted antigen that is highly conserved throughout all mycobacterial species. Importantly for BCG priming, Ag85A is present in all strains of BCG and in all strains of environmental mycobacteria sequenced to date (4).

1.2 Description of MVA85A/AERAS-485

The MVA85A/AERAS-485 vaccine consists of the attenuated vaccinia virus MVA vector with a 1176 base-pair insert, which is almost the complete tuberculosis gene for Ag85A, with the tissue plasminogen activator (TPA) signal sequence preceding the N terminus and a monoclonal antibody tag (pk) at the C terminus. Expression of the tuberculosis DNA sequence is regulated by the vaccinia P7.5 early/late promoter.

Ag85A is a secreted 32-kDa protein mycobacterial antigen that is found in abundance in culture fluids of *Mycobacterium tuberculosis* (*Mtb*). It is a member of mycolyl transferase complex and is highly immunogenic. It contains several CD4 T cell epitopes and at least one CD8 T cell epitope and protects against *Mtb* challenge in both mice and guinea pigs (5, 6). It is known that BCG-vaccinated mice lose immunity against challenge infection as they age. Brooks and colleagues showed that when these animals were vaccinated with the Ag85A in midlife, they regained protection in the lungs comparable to those of young mice with minimal pathological damage. Immunization of inbred mice with DNA encoding the Ag85A induced statistically significant protective immunity against challenge with *Mtb*. Protection was assessed by measuring the amount of live *Mtb* in the lungs (7). The magnitude of protection with the DNA vaccine was 1–1.5 logs more than placebo-vaccinated animals and similar to BCG vaccine. DNA-Ag85A vaccine induced less protection than BCG in a highly-susceptible guinea pig model (8). However, the prime–boost immunization in mice involving MVA expressing Ag85A

resulted in both CD4+ and class I-restricted CD8+ T cell responses and protected these animals against TB. The protection was equivalent to that of traditional BCG vaccination (6). Furthermore, MVA85A/AERAS-485 in humans significantly boosted BCG-primed anti-TB immunity after it was given to 31 adult volunteers and produced levels of antigen-specific IFN-γ-secreting T cells 5–30 times higher than in people given a BCG vaccination (9). Ag85A is an important protective antigen.

1.3 Nonclinical Experience with MVA85A/AERAS-485

Protective efficacy studies using a BCG prime-MVA85A/AERAS-485 boost strategy, followed by *Mtb* challenge, have been conducted in 4 animal species (mice, guinea pigs, non-human primates and cattle). These experiments suggested improved protection of the combination compared to BCG alone.

The toxicity of MVA85A/AERAS-485 was tested in mice following 2 ID injections of 1 x 10^7 pfu MVA85A/AERAS-485 over 15 days. Control animals received PBS. The mice were sacrificed 14 days after the second dose. MVA85A/AERAS-485-injected mice showed no differences from PBS-injected control animals in body weight, organ weight, histopathology, or haematological and biochemical parameters. A low level of local irritation at the site of administration was observed with MVA85A/AERAS-485.

1.4 Clinical Experience with MVA85A/AERAS-485

A total of 20 clinical studies (15 Phase I, 5 Phase II) of MVA85A/AERAS-485 have been completed or are ongoing. A total of 309 healthy adults and adolescents, and 1674 children and infants, have received MVA85A/AERAS-485 in the completed studies, and over 300 subjects (including infants and HIV-infected adults) have been vaccinated in ongoing studies. The doses of MVA85A/AERAS-485 in these studies range from 1 x 10^7 to 1 x 10^8 pfu.

MVA85A/AERAS-485 induced high levels of antigen-specific IFN-γ secreting T cells in BCG-naïve adults, and even higher levels in adults previously vaccinated with BCG either 1 month previously or 1-20 years previously. Immunogenicity of MVA85A/AERAS-485 in BCG-vaccinated adolescents and adults latently infected with *Mtb* was similar to that seen for BCG-vaccinated healthy adults.

Over 2000 individuals have received MVA85A in clinical trials to date, including BCG-naïve adults; BCG-vaccinated adults, adolescents, children, and infants; HIV positive adults; and adults latently infected with *Mtb*. Final safety data are available for 1870 of these subjects. No serious adverse events judged to be related to MVA85A have been reported in these subjects. The safety profile of MVA85A developed so far consists of local adverse events in most subjects, predominantly pain, erythema, swelling, and injection site warmth. Of these the vast majority were mild or moderate, although occasionally severe local reactions occurred. Less frequent systemic reactions resembled flu such as fever, headache, rash, malaise, fatigue, anorexia, cough, arthralgia, and myalgia. These systemic events were also usually mild or moderate. In a Phase II study in which over 1300 infants received MVA85A, systemic events related to study vaccination reported at a higher incidence (>1 percentage point difference) in the MVA85A

group compared to control included irritability, insomnia, hypersomnia, pyrexia, hypophagia, somnolence, diarrhoea, and vomiting. The majority of these systemic events were mild or moderate, with severe cases reported in a few subjects (2 subjects for vomiting; 1 subject for pyrexia). Some adverse changes in haematology and chemistry parameters have been reported in adults and infants, which were mild to moderate in severity except for severe increases in infants for ALT (4 subjects), ALP (1 subject), and WBC (3 subjects); all severe changes returned to baseline levels on follow-up. In subjects infected with HIV, no clinically significant effect of MVA85A vaccination on CD4 counts or viral load was observed.

In a recently completed Phase 2b study, 1 x 10^8 pfu MVA85A/AERAS-485 administered intradermally to BCG-vaccinated infants was shown to be safe and well-tolerated (10). The study did not, however, demonstrate any additional protection against TB disease or *M. tuberculosis* infection in MVA85A/AERAS-485-vaccinated infants compared to infants who received BCG alone. The immune responses to MVA85A/AERAS-485 as measured by *ex-vivo* ELISpot were modest (median 136 SFC per million PBMC) compared to responses seen in HIV-infected adults.

HIV-infected adults with CD4+ lymphocytes > 300-350/mm^3 at study enrolment have received MVA85A/AERAS-485 and have been followed for at least 28 days post-vaccination, including some subjects who received a booster MVA85A/AERAS-485 vaccination 6-12 months after the first vaccination. The HIV-infected subjects in these studies included both adults receiving antiretroviral therapies (ART) and those not receiving ART. Local and systemic adverse events were similar to those previously observed in HIV-uninfected adults, including the frequency and severity of adverse events. Subjects on these studies were also followed for changes in CD4+ lymphocyte counts and HIV viral load. Injection with MVA85A/AERAS-485 did not appear to be associated with clinically significant declines in CD4+ lymphocytes or increase in HIV viral load. An antigen-specific immune response to MVA85A/AERAS-485 vaccinations was demonstrated in these studies, and booster vaccinations with MVA85A/AERAS-485 in an ongoing study showed an enhanced immune response (see TB019 below).

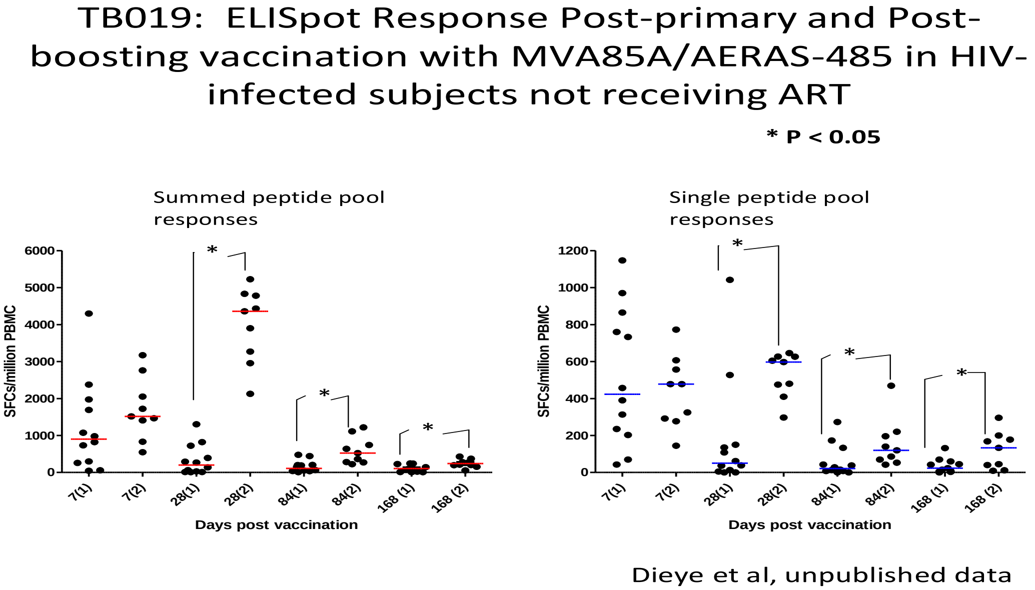

A comparison of the responses obtained 7, 28, 84 and 188 days after the first (1) and second (2) doses of MVA85A in HIV infected subjects (ART-).

1.5 Rationale for Study

The available live tuberculosis vaccine, BCG, provides incomplete protection against pulmonary tuberculosis. Due to natural inhibition of a second dose, a BCG revaccination does not provide much additional protection. MVA85A/AERAS-485 presents a tuberculosis antigen in the setting of a live but non-replicating virus vaccine to increase T-cell immunity and thus protection against tuberculosis. MVA85A/AERAS-485 has been administered in clinical trials to over 2000 subjects, including 80 subjects who were HIV-positive, without any vaccine-related serious adverse events (including no clinically significant effect on either CD4 count or HIV viral load) and shows evidence of immunogenicity. Following licensure, MVA85A/AERAS-485 will be made available in areas of high HIV prevalence. It is therefore important to study the efficacy and safety of the vaccine in HIV-positive individuals. This study will evaluate the safety of MVA85A/AERAS-485 in HIV-positive adults with baseline CD4 counts above 350 cells/mm^3 if not receiving ART or above 300 cells/mm^3 if receiving ART. In addition, the protective efficacy against TB disease and the immunogenicity of MVA85A/AERAS-485 will be evaluated.

2 STUDY OBJECTIVES AND DESIGN

2.1 Objectives

Primary Objective

The primary objective of this study is to evaluate the safety of MVA85A/AERAS-485 compared to placebo.

Secondary Objectives

The secondary objectives of this study are:

1. To evaluate the efficacy of MVA85A/AERAS-485 in the prevention of TB disease compared to control subjects who receive placebo in HIV-infected, African adult subjects without active TB disease.
2. To evaluate CD4+ lymphocyte counts and HIV-1 viral load before and after administration of MVA85A/AERAS-485 compared to placebo.
3. To evaluate the efficacy of MVA85A/AERAS-485 in the prevention of TB disease in subjects receiving ART at baseline compared to subjects receiving ART at baseline but who receive placebo.
4. To evaluate the efficacy of MVA85A/AERAS-485 in the prevention of TB disease in subjects who received isoniazid preventive therapy compared to control subjects who also received isoniazid preventive therapy but who receive placebo.
5. To evaluate the immunogenicity of MVA85A/AERAS-485 compared to placebo as described by the *ex vivo* IFN-γ ELISPOT assay.
6. To evaluate the immunogenicity of MVA85A/AERAS-485 compared to placebo as described by flow cytometric intracellular cytokine staining of CD4+ and CD8+ T cells after stimulation with a peptide pool of mycobacterial antigens.
7. To identify potential immunological correlates of protection from tuberculosis in subjects vaccinated with MVA85A/AERAS-485.
8. To evaluate the QuantiFERON conversion rate at final study assessment in MVA85A/AERAS-485 recipients compared to control subjects without a diagnosis of tuberculosis during the trial.

2.2 Design

This Phase II multi-country trial will be conducted as a randomized, double-blind, placebo-controlled proof-of-concept trial in 650 HIV-positive adults with no evidence of active TB disease. Subjects will be stratified at the time of randomization by whether or not they are currently receiving ART and then randomized in a ratio of 1:1 to receive either MVA85A/AERAS-485 at 1 x 10^8pfu or placebo (Candin), as shown in Table 2-1. Randomization of each group will be capped so that at least 50% of the subjects randomized will be receiving ART at randomization. Subjects will receive an intradermal injection of MVA85A/AERAS-485 or placebo on Study Day 0, followed 6-9 months later by a booster injection of MVA85A/AERAS-485 or placebo. The minimum follow-up period for each subject will be 6 months after their last vaccination, during which subjects will be followed for safety, clinical signs and symptoms of TB, and immunogenicity. All subjects will continue to be followed every 3 months until the last subject enrolled has been followed for 6 months after their last vaccination.

Table 2-1 Treatment Groups

Treatment Group	Number of Subjects	Dosing Regimen
MVA85A/AERAS-485 1 x 10^8pfu	325	Study Day 0 (prime), SD168-252 (boost)
Placebo	325	Study Day 0 (prime), SD168-252 (boost)

3 STUDY PROCEDURES

3.1 Schedule of Subject Evaluations

A Summary Schedule of Evaluations depicting all visit-specific procedures is provided in Table 3-1 and Table 3-2. See Appendix A for a more detailed description of the evaluations.

Table 3-1 Summary Schedule of Subject Evaluations: Randomization/initial vaccination (SD0) through Booster Vaccination (V4)

Evaluation	Screen[a]	Study Visits				
		**Randomization/ initial vaccination (SD0)**	**SV1 (7d post-vaccination)**	**SV2 (28d post vaccination)**	**SV3 (84d post vaccination)**	**SV4 (168-252d post vaccination)**
Written informed consent	x					
Verify eligibility criteria		x				x
Medical history	x					
Physical examination	x					
Chest x-ray	x					
Urine βHCG (<u>all</u> females physically capable of pregnancy)	x	x[c]				x[c]
CD4+ lymphocyte count	x[d]	x[c]	x	x	x	x[c]
HIV-1 viral load	x	x[c]	x	x		x[c]
Hepatitis B; HIV-1 ELISA w/confirmatory test	x					
Serum chemistry[b]	x		x	x		x[c]
CBC, differential, platelets	x		x	x		x[c]
Urine dipstick	x					
QuantiFERON®-TB Gold In Tube[e]	x					
Tuberculin PPD skin test[e]	x					
Vital signs (temp, BP, pulse)	x	x	x	x	x	x
**Study vaccine administration**		**x**				x
Immunology (PBMC, plasma)		x[c]	x	x		x[c]
Clinical observations for signs and symptoms of TB		x	x	x	x	x
Solicited adverse events (incl. con. meds.)		x	x	x		x
Adverse events (incl. con. meds.)		x	x	x		x
Serious adverse events (incl. con. meds.)		x	x	x	x	x
Site of injection examination		x	x	x		x
Diary Distribution/Review		x	x			x
Maximum blood (mL) collected per visit	37	46-50	58-62	58	5	58-62
Maximum cumulative blood (mL) collected	37	83-87	141-149	199-207	204-212	262-274

a. Screening evaluations to be conducted within 45 days prior to randomization. For subjects who have documented positive results for HIV-1 ELISA with confirmatory test, QuantiFERON-TB Gold, and tuberculin PPD tests will not need to have these test repeated
b. GGT, ALT, AST, total bilirubin, ALP, creatinine
c. All samples on Study Day 0 and SV4 (study days 168-252) are obtained before vaccination; samples for CD4+ lymphocyte count and HIV-1 viral load do not need to be repeated if samples for screening were obtained within 14 days before Study Day 0. Pregnancy test must be from urine obtained within 2 days prior to each visit.
d. 2 CD4+ lymphocyte counts must be performed at least 4 weeks apart, one performed within 6 months prior to randomization and one within 45days prior to randomization
e. Blood for QuantiFERON should be obtained before PPD skin test is applied.

Table 3-2 Summary Schedule of Subject Evaluations: Post Booster Vaccination through the End of Study Follow-up

Evaluation	Study Visits				
	SV5 (7d post boost)	SV6 (28d post boost)	SV7 (84d post boost)	SV8-End of study (q3 months)[a]	End of Study[a]
Medical history					x
Physical examination					x
CD4+ lymphocyte count	x	x	x	at 6 months post-boost	
HIV-1 viral load	x	x		at 6 months post-boost	
Serum chemistry[b]	x	x			
CBC, differential, platelets	x	x			
QuantiFERON®-TB Gold In Tube[c]					x
Tuberculin PPD skin test[c]					x
Vital signs (temp, BP, pulse)	x	x	x	x	x
Immunology (PBMC, plasma)	x	x			
Clinical observations for signs and symptoms of TB	x	x	x	x	x
Solicited adverse events (incl. con. meds.)	x	x			
Adverse events (incl. con. meds.)	x	x			
Serious adverse events (incl. con. meds.)	x	x	x	x	x
Site of injection examination	x	x			
Diary Review	x				
Maximum blood (mL) collected per visit	58-62	58	5	10	3
Maximum cumulative blood (mL) collected through 24 months of follow-up	320-336	378-394	383-399	393-399	396-412

a. Subjects will continue to be followed every 3 months until the last subject enrolled has been followed for 6 months after their last vaccination.
b. GGT, ALT, AST, total bilirubin, ALP, creatinine
c. Blood for QuantiFERON should be obtained before PPD skin test is applied.

3.2 Subject Selection

3.2.1 Recruitment and Informed Consent

Various methods of recruitment may be used such as advertising, referrals, or solicitation of subjects previously known to the clinical site. Interested subjects will be invited to participate in the informed consent process. Informed consent will be obtained by the use of a written consent form approved by the Institutional Review Board (IRB) or Independent Ethics Committee (IEC) and signed and dated by the subject at the time of consent. The clinical investigator, or designee, will conduct the consent discussion on an individual basis with each subject and will allow adequate time for all questions to be addressed. Written informed consent will be obtained prior to conducting any study-related procedures. A copy of the signed consent form shall be given to the subject prior to Study Day 0.

3.2.2 Screening

Subjects who have signed a consent form will be screened to assess eligibility for the study. A screening log will be maintained by the site that records all subjects who signed an informed consent and entered the screening process.

For screened individuals who are ineligible for the clinical trial, any abnormal results and findings will be discussed with the subject with referral for follow-up care with their healthcare provider as necessary.

Subjects who fail initial screening due to a temporary, reversible cause (e.g., febrile illness) may resume screening once the condition has resolved.

3.2.3 Inclusion Criteria

Eligibility for entry into the study will be based on the inclusion and exclusion criteria described below. The investigator must document confirmation of eligibility prior to randomization on Study Day 0.

Subjects must meet all of the following criteria at the time of randomization:

1. Has completed the written informed consent process prior to undergoing any screening evaluations.
2. Either males or females aged ≥18 and ≤50 years on Study Day 0
3. In general good health, confirmed by medical history and physical examination
4. Has ability to complete follow-up period as required by the protocol
5. Has laboratory evidence of human immunodeficiency virus (HIV) infection, defined as a positive HIV-1 ELISA test plus a positive confirmatory test (e.g., a second HIV-1 ELISA, PCR, or rapid ELISA) diagnosed prior to randomization
6. Is willing to allow the investigators to discuss the subject's medical history with the subject’s HIV physician
7. If not receiving ART at the time of randomization, must have 2 CD4+ lymphocyte count test results >350 cells/mm^3, performed at least 4 weeks apart, one performed within 6 months prior to randomization and one within 45 days prior to randomization
8. If receiving antiretroviral therapies (ART) at the time of randomization, must have 2 CD4+ lymphocyte count test results >300 cells/mm^3, performed at least 4 weeks apart, one performed within 6 months prior to randomization and one within 45 days prior to randomization
    a. Subjects on ART must have been receiving ART for at least 6 months prior to randomization and must have an undetectable HIV viral load within 45 days prior to randomization
    b. Women who received ART as part of the PMTCT program must have completed therapy at least 2 months prior to randomization
9. Has either
    a. a negative QuantiFERON-TB Gold In-Tube test result and tuberculin PPD skin test ≤5 mm induration within 45 days prior to randomization or

b. a positive QuantiFERON-TB Gold In-Tube test result and/or tuberculin PPD skin test >5 mm and has completed at least 5 months of isoniazid preventive therapy within 3 years prior to randomization or
c. a positive QuantiFERON-TB Gold In-Tube test result and/or tuberculin PPD skin test >5 mm and has completed treatment for TB disease within 3 years prior to randomization

10. Females: Ability to avoid pregnancy during the trial. Women physically capable of pregnancy (not sterilized and still menstruating or within 1 year of the last menses if menopausal) in sexual relationships with men must avoid pregnancy by using an acceptable method of avoiding pregnancy from 28 days prior to administration of the study vaccine through 6 months after the last study vaccination. Acceptable methods of avoiding pregnancy include a sterile sexual partner, sexual abstinence (not engaging in sexual intercourse), and any contraceptive method deemed clinically suitable by the trial clinician taking into account ART status.
11. Has completed the written informed consent process for simultaneous enrollment in Aeras Vaccine Development Registry protocol

3.2.4 Exclusion Criteria

Subjects must have <u>none</u> of the following at the time of randomization:

1. Acute illness
2. Fever (temperature > 37.5° C)
3. Significant symptomatic infection (including laboratory evidence of HIV-2)
4. Any evidence of active TB disease, as determined by any clinical, radiological, or microbiology measurements.
5. Any AIDS defining illness by WHO criteria
6. Use of any investigational or non-registered drug, vaccine or medical device other than the study vaccine within 182 days preceding dosing of study vaccine, or planned use during the study period
7. Previous receipt of a recombinant MVA or FP vector at any time.
8. Is enrolled in any other clinical product trial
9. Administration of methotrexate, azathioprine, cyclophosphamide, oral corticosteroids (for corticosteroids, this will mean prednisolone, or equivalent, ≥0.5 mg/kg/day; inhaled and topical steroids are allowed) and other immunosuppressive therapies, or blood products or blood derivatives within the six months prior to randomization
10. History of allergic disease or reactions likely to be exacerbated by any component of the vaccine, e.g. egg products
11. Presence of any history of cancer [except basal cell carcinoma of the skin and cervical carcinoma in situ], or renal failure
12. Evidence of severe depression, schizophrenia or mania
13. Pregnant females and females who are breast-feeding
14. Any history of anaphylaxis in reaction to vaccination
15. Principal investigator assessment of lack of willingness to participate and comply with all requirements of the protocol, or identification of any factor felt to significantly increase the participant's risk of suffering an adverse outcome

3.2.5 Screening Clinical Assessments and Laboratory Tests

Unless noted otherwise, the window period within which all screening evaluations must be completed by the investigator to confirm subjects' eligibility is 45 days prior to randomization (Study Day 0). The results of screening evaluations must be reviewed by the investigator to confirm eligibility on Study Day 0.

Subjects will provide a detailed medical history and undergo a physical examination. The assessment will include the determination of any surgeries or medically significant procedures planned to occur during the entire study period. Demographic characteristics (date of birth, gender, and race/ethnicity) will also be collected.

Screening laboratory tests will be performed during the screening process. Results from these laboratory tests will serve as study-entry baseline values. All screening laboratory specimens will be processed according to laboratory SOPs available from the clinical laboratory(ies) designated for the study. Information about the laboratory(ies), including any instructions for performing and interpreting specific tests, will be maintained in the investigator's study binder. Any new abnormal findings resulting from the screening procedures will be discussed with the subject and referral will be made for follow-up care if necessary.

3.3 Study Randomization

Following completion of screening, eligible subjects will be randomized in a 1:1 ratio to MVA85A/AERAS-485 or placebo (Candin) based on a randomly-generated sequence of subject identification numbers (randomization schedule).

Subjects will be stratified based on whether or not they are receiving ART at the time of randomization. Randomization of each group will be capped so that at least 50% of the subjects randomized will be receiving ART at randomization. Randomization will also be stratified by study center. Randomization will be performed using an interactive voice response system (IVRS); the randomization schedule for the IVRS will be prepared by a statistician who will not be involved with the analysis of the study in order to maintain blinding of the study team. Subjects will be considered randomized when a subject identification number is assigned on Study Day 0.

In addition to their treatment assignment, the first 5% of the subjects enrolled (a total of 70 subjects, across ART+/ART- strata, with a minimum of 30 subjects at each clinical site) will have blood collected for ICS assay and for real-time ELISPOT assay.

3.4 Blinding

The study vaccine manager and the study monitor will be the only persons unblinded at the site during the study and must not reveal individual subject treatment assignments to any other member of the study team. The study vaccine manager must be a designated study team member who is not an employee of Aeras and who will have no other clinical or regulatory

responsibilities associated with the conduct of the study during the entire study period. Unblinded study personnel must not participate in the evaluation of adverse events. A Delegation of Authority Log will be maintained by the site and will identify the individual(s) authorized to function as the study vaccine manager, i.e., individuals with access to study blinding information. All pharmacy source documents that can link a subject identification number with a treatment assignment must remain secure in the pharmacy with access limited to only the study vaccine manager and the study monitor until notification from the sponsor that the study has been unblinded.

The MVA85A/AERAS-485 and the placebo will be packaged and labeled to appear indistinguishable from each other at the time of injection. Identical syringes and needles will be used for preparation and administration of injections of vaccine/placebo, and labels accompanying the syringes of prepared vaccine/placebo doses will not indicate which is in the syringe.

3.4.1 Unblinding for Clinical Emergencies

If there is an urgent clinical requirement to know a subject’s treatment assignment, the investigator (in consultation with the medical monitor, if possible) will make a written request to the vaccine manager for urgent unblinding of a subject’s treatment. The request must include the subject identification number, the date, a brief justification of the clinical requirement to the vaccine manager in the research pharmacy, and the investigator’s signature. The request will be kept in the study file.

Upon receipt of proper written request, the vaccine manager or designee will disclose the treatment group to the investigator. Aeras must be notified immediately of any clinically required break of the study blind on an Immediately Reportable Event Form.

3.5 Study Vaccine Administration

Subjects receive their dose of study vaccine/placebo as soon as possible after randomization (Study Day 0) and a booster vaccination 6-9 months after initial vaccination unless they are no longer eligible for vaccination. Prior to booster vaccination, the clinical investigator will assess each subject’s current health status and medical history to determine if there are any medical contraindications to receiving booster vaccinations. All females who are physically capable of pregnancy must have a negative urine βHCG within 2 days prior to the booster vaccination. Subjects will receive the same study vaccine/placebo for the booster vaccination as they received for their initial vaccination based on the study randomization schedule. *The Sponsor should be contacted whenever the investigator believes that the vaccinations for a subject will not be given in the protocol-specified timeframes.*

Before administering any injections, the study team member in the clinic who will be administering the vaccine/placebo must inspect the syringe and volume, checking that the syringe is identified with the correct subject identification number and initials and checking the date and time the dose was prepared. If circumstances result in a delay of administration beyond the allotted time, an explanation must be entered into the source documents and the expired

syringe must be returned to the study vaccine manager, who will prepare a replacement syringe. The study vaccine manager will dispose of the expired syringe only upon authorization from the study monitor. If administration of study vaccine/placebo does not occur on the day of randomization, the principal investigator must notify the sponsor immediately.

The vaccine/placebo will be administered intradermally into the skin. The vaccine administrator will wear gloves and eye protection. Subjects will stay in the unit for 60 minutes after vaccination for observation. As with any vaccine, allergic reactions to dose administration are possible. Therefore, appropriate drugs and medical equipment to treat acute anaphylactic reactions must be immediately available and a medically qualified study team member trained to recognize and treat anaphylaxis must be present in the clinic during the entire vaccination procedure and post-vaccination monitoring period.

In order to minimize dissemination of the recombinant vectored vaccine virus into the environment the inoculation site will be covered with a dressing after immunization. This should absorb any virus that may leak out through the needle track. The dressing will be removed from the injection site at the end of the 60 minute observation period and will be disposed as GMO waste by autoclaving, in accordance with the relevant SOP and current local practice.

Administration of the study vaccine/placebo (including date, time, and which arm was injected) must be documented in the subject's study records by the study team member who administered the vaccine/placebo.

3.6 Study Evaluations

3.6.1 Pre-vaccination and Post-Vaccination Monitoring of Subjects

Subjects will have vital signs (temperature, blood pressure, pulse) taken prior to each study vaccination. Subjects will remain in the clinic under close observation for at least 60 minutes after receiving study vaccine. Vital signs will be repeated before subjects leave the clinic. Allergic reactions to vaccination are possible, therefore, appropriate drugs and medical equipment to treat acute anaphylactic reactions must be immediately available and a medically qualified study team member trained to recognize and treat anaphylaxis must be present in the clinic during the entire vaccination procedure and post-vaccination monitoring period.

3.6.2 Clinical Assessments and Laboratory Tests

An examination of the injection site will be performed 60 minutes after each study vaccine/placebo administration and at 7 and 28 days after each vaccination. Subjects will have CD4+ lymphocyte counts done prior to each vaccination, at 7, 28, and 84 days after each vaccination and then at 6 months post-boost. Vital signs will be recorded prior to each vaccination, at 7, 28, 84 days after each vaccination, and at all subsequent study visits. Subjects will also have tests done for HIV-1 viral load results done prior to each vaccination, at 7 and 28 days after each vaccination, and then at 6 months post-boost.

Blood for hematology and serum chemistry will be collected at screening and prior to booster vaccination and 7 and 28 days after each vaccination. All laboratory samples will be processed according to SOPs provided by the local clinical laboratory.

All subjects randomized to the study will have a Tuberculin PPD skin test and blood for QFT-G collected during screening, and then again at the End of Study visit. Subjects will also be observed for signs and symptoms of TB at every study visit.

3.6.2.1 Abnormal Clinical Assessments and Laboratory Tests

Results from clinical laboratory tests obtained on the study must be reviewed by the investigator (or a designee who is a medically qualified study team member) within 72 hours of receiving the results to determine if abnormalities exist. If the laboratory value is abnormal and has increased in toxicity grade (see Appendix C for toxicity grading scales) from pre-vaccination values, it must be reported as an adverse event and repeated promptly to demonstrate resolution. Additional laboratory tests may be performed if the investigator deems them to be necessary to fully evaluate an adverse event. In the event that the investigator elects to order non-protocol-specified laboratory tests, the investigator must record the rationale for the tests and a determination of clinical significance of the result in the source documents. The investigator must keep the medical monitor informed of adverse events of clinical significance.

Abnormal results and findings will be discussed with the subject, or the subject will be referred for follow-up with their healthcare provider if necessary.

3.6.3 Evaluation of Tuberculosis/Mortality

Follow-up visits will be conducted either at the homes of the subjects or at a mutually convenient venue such as a nearby clinic. At each visit the clinical research worker or clinician will question the subject and conduct a basic examination with the purpose of confirming that the subject is not suffering from TB and has not been exposed to TB in the interval since the last visit. Those who have signs or symptoms of TB or who are found to have been in contact with a known smear positive case of TB will be referred to the clinic for comprehensive investigations for TB disease, including sputum smear and culture and a chest x-ray.

Criteria for Pulmonary TB workup

All subjects with 1 or more of the following symptoms and signs will be investigated for possible pulmonary TB:
· Cough > 1 week
· Fever > 1 week
· Drenching night sweats
· Unintentional weight loss of > 3.0 kg in the past 4 weeks or since last visit
· Pleuritic chest pains
· Hemoptysis
· Shortness of breath

Pulmonary TB Workup

1st visit for any of above symptoms:

- PA and lateral Chest X-Ray (or schedule for 2nd visit)
- Instructions on deep coughing
- 1st sputum specimen collected (spot)
- 2nd sputum collected by cough induction
- Provide specimen cup for morning sputum specimen (if sputum induction is unsuccessful or not done, and subject scheduled for a 2nd visit)

2nd visit (within 5 days of 1st visit; to be completed only if sputum induction at 1st visit is unsuccessful or not done, or for chest x-rays)

- Retrieve morning sputum specimen (2nd specimen)
- 3rd specimen collected (spot)
    - Sputum induction (if cough is inadequate)

In addition, possible extrapulmonary disease will be investigated where possible and where facilities are available by invasive means as indicated (e.g., lumbar puncture, fine-needle aspiration cytology, pleural biopsy, pericardial tap, ultrasound guided biopsy, plain radiography, and CT scanning).

If it is discovered that the subject has died, a full mortality investigation will be conducted, including a minimum of a verbal autopsy interview with the next of kin. A surveillance system will be established to identify subjects who die and to have post-mortem examinations performed to identify cause of death where feasible. If it is discovered that the subject has moved away from the area, attempts will be made to contact them by other means to confirm their vital status and whether they have signs/symptoms of TB, exposure to TB or have been treated for TB.

In addition to the above, the following general surveillance will be conducted in the trial areas:

1. The TB registers of all clinics in the areas will be checked regularly for all new cases of TB and details matched against the study database looking for new cases of TB and household contacts.
2. Records at the Department of Home Affairs Offices, mortuaries, funeral parlors and undertakers' firms will be checked regularly for all new deaths and details matched against the study database.
3. Records at the regional and provincial public health laboratories will be checked regularly for all new TB smear and culture results and details matched against the study database looking for new cases and contacts.
4. Records at the relevant district, secondary, tertiary and specialist TB hospitals will be checked regularly for all new diagnoses of TB and diseases which might be TB (e.g. bronchopneumonia) and details matched against the study database looking for new cases and contacts.

Study subjects identified as a TB case via general surveillance may be contacted by study staff to arrange for TB workup at the study center.

3.6.4 Immunology Laboratory Evaluations

There are two different parts to the immunogenicity evaluations that will be conducted as part of this clinical trial, which are described below:

1. Real-time evaluation of vaccine immunogenicity in a sample of subjects from each clinical trial site
2. Sample storage until the end of the trial to allow the detection of potential immunological correlates of protection.

3.6.4.1 Real Time Evaluation of Vaccine Immunogenicity

There are three assays which will be used to determine vaccine immunogenicity on the first 5% of the subjects enrolled (a total of 70 subjects, across ART+/ART- strata as defined in Section 3.3, with a minimum of 30 subjects at each clinical site). Samples will be obtained from these 70 subjects prior to each vaccination, and at 7 and 28 days after each vaccination. All evaluations will be performed by technicians blinded to treatment group.

1. Ex-vivo IFN-γ ELISPOT assays will be conducted at all clinical trial sites on fresh PBMC.
2. Whole blood from all 70 subjects collected only prior to each vaccination and 7 days after each vaccination will be frozen for use in the WB ICS assay, which will be performed centrally.
3. Kris Huygen's laboratory at the Pasteur Institute, Brussels, will analyze the Ag85A specific antibody response on frozen plasma or serum samples collected from vaccinees before and after MVA85A/AERAS-485 administration. Recombinant histidine-tagged Ag85A from *M. tuberculosis* (identical to Ag85A of *M. bovis* BCG) will be prepared using affinity chromatography on Ni-columns. In parallel, native Ag85A will be purified from *M. bovis* BCG culture filtrate by sequential chromatography on Phenyl-Sepharose, DEAE sephacel and molecular sieving. Sera will be analyzed for presence of Ag85A specific IgG antibodies by ELISA. A selected number of serum samples will also be analyzed for the presence of specific antibodies against Ag85A from *M. avium* as a read-out for pre-existing immunity against environmental mycobacteria. Western blot analysis against culture filtrate of BCG will also be performed on this group of serum samples.

Any residual PBMC, WB ICS samples and plasma from these 70 subjects will be stored for cryopreservation as described in 3.6.4.2 below wherever possible.

Table 3 Summary of Real Time Immunology Evaluations

Sample type	Assay	Purpose of Assay	Study Days
Immunology (whole blood/PBMC)	Intracellular cytokine staining (ICS)	Determine cellular immune response to study vaccine	*Prior to each vaccination, and at 7 and 28 days after each vaccination. (PBMC and ELISPOT only at 28 days post-vaccination.)*
	Cytokine ELISPOT		
Immunology (serum/plasma)	Ag85A antibody response	Exploratory measurement of immune response to study vaccine	*Prior to each vaccination, and at 7 and 28 days after each vaccination.*

3.6.4.2 Immune Correlate Samples

Two different samples will be collected on the specified study days (Tables 3-1 and 3-2) and stored for all subjects randomized (except for the 70 participants mentioned above):

1. PBMC sample
2. Serum/plasma sample

When the trial is completed and unblinded, TB cases and matched controls will be identified and selected immunological analyses will be conducted. Such analyses, to be conducted at Oxford University, Aeras, and the clinical trial sites, may include polychromatic flow cytometry to determine the functionality and phenotype of the antigen specific T cells, CFSE proliferative assays, microarray and RT-PCR analysis. Other exploratory immunological assays may also be conducted to fully characterize the vaccine induced immune response.

A summary of immunologic assays to be performed on blood specimens is shown in Table 3-2. Staff at the clinical research site will refer to the most current version of the Specimen Management Manual (provided under separate cover) for further instructions and additional information on specimen collection and processing.

3.6.5 Concomitant Medications

The collection of information on concomitant medications used by subjects following vaccination will coincide with the collection period of adverse events. The collection period for concomitant medications associated with the treatment of adverse events will be 28 days following each vaccination. The collection period for concomitant medications associated with the treatment of serious adverse events (SAE) and anti-retrovirals for the treatment of HIV will be Study Day 0 through the end of the study.

Concomitant medication includes prescription and non-prescription drugs or other treatments, and any vaccines other than the study vaccines. The name of the medication, treatment start and stop dates (or ‘ongoing’), route of administration, and indication must be recorded on the

Concomitant Medications electronic case report form (eCRF). The indication recorded on the Concomitant Medications eCRF must correspond to a medical term/diagnosis recorded on the adverse event (AE) eCRF, or to a pre-existing condition noted in the subject's medical history, or be noted as prophylaxis, e.g., dietary supplement.

3.6.6 Subject Follow-up and Contact

All subjects who are assigned a subject identification number and receive study vaccine will be followed according to the protocol unless consent is withdrawn.

Subjects will be instructed to contact a study team member to report new diagnoses or new or worsening adverse events and to come to the study clinic if medical attention is needed, provided the urgency of the situation permits. For emergencies and other unscheduled visits to a medical facility other than the study clinic, medical records will, to the extent possible, be obtained by the investigator.

During each clinic visit, subjects will be reminded to notify a study team member of the following:

- The occurrence of AEs and SAEs during the respective reporting periods
- Receipt of any concomitant medications during the applicable reporting period
- Plans to move or if contact information changes
- If subject has decided to withdraw from the study
- Change in general health status
- Any other change in status that may affect the subject's participation (e.g., plan to participate in another investigational study)

All deviations from protocol procedures, evaluations, and/or visits must be categorized and documented as they occur. Each deviation must be documented on a Protocol Deviation Form. A cumulative record of all protocol deviations must be maintained on a Protocol Deviation Log. When possible, missed visits and procedures must be rescheduled and performed at the nearest possible time point to the original schedule.

Protocol deviations are to be categorized as follows:

1. Informed consent was not properly obtained.
2. A subject did not meet inclusion/exclusion criteria but was entered into the study.
3. A subject developed withdrawal criteria during the study but was not withdrawn.
4. There was a deviation in randomization, vaccine preparation, or dosing.
5. A protocol-specified visit or procedure was performed out of the allowable window.
6. A protocol-specified visit or procedure was missed (not performed).
7. Other (to be specified in the appropriate documentation)

The signature of the Principal Investigator is required on the Protocol Deviation Form for categories 2 through 5. Certain protocol deviations may also meet the criteria for an Immediately Reportable Event as described in Section 5.12.

3.6.7 Loss to Follow-up

If the site's study team members are unable to establish contact with a subject who misses a scheduled study visit, the clinical site must make every possible effort to re-establish contact with the subject and document such efforts. If contact is re-established, then the subject will resume participation in the study.

If contact with the subject cannot be re-established by the subject's expected End-of-Study visit date, then a determination of "lost to follow-up" can be made.

4 STUDY VACCINES

Staff at the clinical research site will refer to the most current version of the Study Vaccine Management Manual (provided under separate cover) for further instructions on vaccine/placebo storage and preparation. Additional information is also provided in the Investigator's Brochure.

4.1 Supplies

Aeras will provide the study vaccine manager with adequate quantities of MVA85A/AERAS-485 and Candin (placebo) for the study prior to the start of vaccination.

MVA85A/AERAS-485 is manufactured in accordance with GMP by Impfstoffwerk Dessau-Tornau (IDT) Biologika GmBH, Am Pharmapark, D-06861 Dessau-Rosslau, Germany. A description of the contents of the vials follows.

MVA85A/AERAS-485: formulated in Tris buffer (10 mM Tris, 140 mM NaCl, pH 7.7) at a target concentration of between 4 x 10^8 and 1 x 10^9 plaque-forming units (pfu)/mL. The dose of study vaccine to be administered will be 1 x10^8 pfu.

Candin® is manufactured by Allermed Laboratories, Inc., San Diego, CA, USA and is licensed for the evaluation of delayed-type hypersensitivity reactions in adults.

Candin is produced from a culture filtrate of two strains of *Candida albicans* grown on a defined medium of inorganic salts, biotin, and sucrose. Lyophilized source material is extracted with a solution of 0.25% NaCl, 0.125% $NaHCO_3$, and 50% w/v glycerol. The concentrated extract is diluted with a solution of 0.5% NaCl, 0.25% $NaHCO_3$, 0.3% human albumin USP, 8 ppm polysorbate 80 and 0.4% phenol.

Candin is supplied as a clear, colorless solution. Candin is usually administered intradermally at a dose of 0.1 mL, but for this study it will be administered at a dose volume to match the volume of the MVA85A/AERAS-485 dose.

4.2 Accountability

The study vaccine manager is required to maintain accurate study vaccine accountability records. Instructions and required forms to be completed and kept for accountability will be provided to

the study vaccine manager. Upon completion of the study, all study vaccine management records will be copied and the copies returned to Aeras or its designee. The originals must be maintained at the clinical site with the rest of the study records.

4.3 Receipt and Storage

Upon receipt of study vaccine supplies, the study vaccine manager must immediately inspect all vials for damage. Study vaccine/placebo will be shipped with a continuous temperature-monitoring device. The temperature of the shipment must be documented by the study vaccine manager. Any deviations or problems identified must be documented and promptly discussed with Aeras and the study monitor to determine the usability of the supplies or if replacements must be sent.

Study vaccine/placebo must be stored in a secured location with no access for unauthorized personnel. Study vaccine/placebo should be placed in a container clearly marked “For Clinical Trial Use Only”.

MVA85A/AERAS-485 will be stored frozen at -40° C or colder in a frost-free or frostless freezer. Vaccine potency is not affected by long-term storage at temperatures up to -20°C, but viral titers may be affected and, therefore, the vaccine should not be kept at -20°C for more than 72 hours. Storage temperature must be monitored continuously and a log of the monitored temperature maintained. The freezer must be equipped with a continuous temperature monitor (wheel device) or should be alarmed in case the temperature exceeds specified ranges. Complete storage instructions will be provided in the Study Vaccine Management Manual.

Candin must be stored refrigerated at 2˚C to 8˚C (36˚F to 46˚F) and must not be frozen. Storage temperature should be monitored daily, and a log of the monitored temperatures must be maintained.

4.4 Vaccine/Placebo Preparation

Preparation of the study vaccine/placebo for injections should not begin before a subject is confirmed eligible on Study Day 0 or Study Day 168-196 for the booster vaccination. Subjects must also be in the clinic on these days and must agree to receive the vaccine/placebo on the same day.

The study vaccine manager will follow the detailed instructions provided in the Study Vaccine Management Manual to prepare the doses of study vaccine/placebo using aseptic technique. One vaccine vial per subject to be immunized will be used. The study vaccine will be allowed to thaw to room temperature before being withdrawn into a masked syringe and administered within 2 hours. The study vaccine manager will draw the appropriate volume of study vaccine/placebo into the masked syringe, will label the syringe with the subject’s identification number and initials, the date and time of preparation, and the volume in the syringe.

To prepare an injection of Candin, sanitize the vial cap with 70% alcohol on a sterile cotton pad. Using a 1 mL Tuberculin syringe with a short 25- to 26-gauge needle, withdraw the appropriate

volume of Candin. Each vial of Candin may be used to prepare injections for more than one subject.

Precautions should be taken to avoid contact of the study vaccine/placebo with broken skin, and gloves and eye protection (safety goggles) should be worn.

Preparation of the vaccine/placebo including date and time must be documented in the study vaccine management records by the study vaccine manager who prepared the doses.

4.5 Disposal of Unused Supplies

All unused study vaccine and supplies will be returned to Aeras at the end of the study or disposed of upon authorization from Aeras or according to the facility's SOPs. Any disposing of study vaccine conducted at the clinical site will be documented in the study file.

5 SAFETY

5.1 Responsibilities for Ensuring the Safety of Trial Subjects

The national regulatory authority, the vaccine sponsor (Aeras), the institution through which the research is performed and all members of the principal investigator's clinical team share responsibility for ensuring that participants in this trial are exposed to the least possible risk of adverse events that may result from participation in this protocol.

5.1.1 Principal Investigator

The principal investigator has a personal responsibility to closely monitor trial subjects and an inherent authority to take whatever measures necessary to ensure their safety. The principal investigator has the authority to terminate, suspend or require changes to a clinical trial for safety concerns and may delay an individual's study vaccine administration or pause study vaccine administration in the whole trial if the investigator has some suspicion that the study vaccine might place a subject at significant risk. The principal investigator determines severity and causality with respect to the study vaccine for each adverse event. For blinded studies the principal investigator is blinded, in which case the study vaccine may consist of a placebo, an active control, or the investigational product.

5.1.1.1 Pausing Rules for the Principal Investigator

If the principal investigator determines that a SUSAR OR a serious adverse event OR an adverse event pattern of concern that is judged to be POSSIBLY, PROBABLY or DEFINITELY related to study vaccine has occurred, the principal investigator will pause administration of study vaccine in the trial. If the principal investigator pauses study vaccine administration he or she will record this in a memorandum to the study file and notify the sponsor.

If the principal investigator pauses study vaccine administration in a trial under the rules in this section and additional clinical information becomes available that reduces the principal

investigator's assessment of causality, severity or toxicity grade and no longer requires pausing then the principal investigator, with the agreement of the local medical monitor, may resume study vaccine administration with a memorandum to the study file and notification of the sponsor.

5.1.2 Study Sponsor

The sponsor (Aeras) also has an institutional responsibility to ensure subject safety. This responsibility is vested in two medical monitors (one local medical monitor and one global medical monitor) and a data monitoring committee (DMC).

5.1.3 Local Medical Monitor

The local medical monitor is the sponsor's representative and is a credentialed physician or surgeon in their country of residence (or geographical region) with the necessary expertise to act in such capacity. The local medical monitor reviews the safety of the product for protocols in a specific region and determines the expectedness. The local medical monitor may make a sponsor's assessment of severity and causality for adverse events that may upgrade the degree of severity and causality determined by the principal investigator. The local medical monitor, like the principal investigator, is blinded for a blinded study.

5.1.3.1 Pausing Rules for the Local Medical Monitor

If the local medical monitor determines that a SUSAR OR a serious adverse event OR an adverse event pattern of concern that is judged to be POSSIBLY, PROBABLY or DEFINITELY related to study vaccine has occurred, the local medical monitor will pause administration of study vaccine in the trial. If the local medical monitor independently pauses the administration of study vaccine in the study, he or she will record this in a memorandum to the study file and notify the principal investigator and sponsor who will then convene the DMC. In all cases administration of study vaccine may resume only if permitted by the DMC. Any changes to the protocol required by the DMC as a condition of study vaccine resumption must be approved by, or submitted to, the Institutional Review Board/Ethics Committee and the national regulatory authority.

5.1.4 Global Medical Monitor

The purpose of the Global Medical Monitor (GMM) is to authorize SUSAR (Suspected Unexpected Serious Adverse Reaction) reporting to regulatory agencies without unblinding the sponsor, protocol team or the clinical research sites. Aeras retains a GMM through the contract research organization PPD, Inc., located in Cambridge, UK. The GMM reviews and unblinds all SUSAR reports and, if he or she deems it necessary, may review and unblind selected SAE reports. The GMM may contact the clinical research sites directly if additional information is needed for assessment of the SUSAR. For all SUSARS the GMM will make available to the sponsor and the clinical research sites a completed blinded CIOMS II so that the sites and sponsor may remain blinded.

5.1.5 Data Monitoring Committee

If study vaccine administration is paused by the principal investigator, the local medical monitor, or the global medical monitor, a Data Monitoring Committee (DMC) will be convened. The composition of the DMC will be described in the DMC charter but will include at least two voting physicians with experience in evaluation of investigational product safety issues and a voting statistician. The voting members cannot be directly involved with the conduct of the study. Voting members cannot be employees of Aeras. Additional subject area experts may be present to provide expertise if requested by the DMC. The DMC may review an individual SAE or it may choose to review adverse events, serious adverse events, solicited adverse events, and laboratory and vital signs data. The DMC may unblind any amount of safety information needed to conduct their assessment. All procedures associated with this review, including objectives, data handling, and elements to be included for review will be documented in the DMC minutes.

After the 200th subject enrolled has completed one month of follow-up post-primary vaccination, a DMC will evaluate blinded and unblinded-by-group safety data (including CD4 counts) to determine if a pattern of adverse events related to MVA85A/AERAS-485 or other safety or risk concerns exist. The DMC will conduct a second unblinded-by-group safety and risk review after the 600th subject enrolled has completed one month of follow-up post-primary vaccination. If a safety risk exists, the DMC will determine if changes to the study protocol need to be made for subjects' safety. Only the DMC and the independent statistician responsible for preparing these analyses would be unblinded during these reviews.

Based on its review and the protocol stopping rules (Section 6) the DMC will make recommendations in the DMC minutes to Aeras regarding further conduct of the study and further administration of study vaccine. The conclusions of the DMC will be communicated to the investigators and the Institutional Review Boards/Ethics Committees and the national regulatory authority for their concurrence. The sponsor agrees to abide by the decision of its DMC with concurrence of the national regulatory authority, the Institutional Review Board or Ethics Committee and the principal investigator.

5.1.6 Institutional Review Boards and Ethics Committees

The Institutional Review Board or Ethics Committee has institutional responsibility for the safety of research subjects. The Institutional Review Board or Ethics Committee has the authority to terminate, suspend or require changes to a clinical trial.

5.1.7 National Regulatory Authority

Since the national regulatory authority (such as the FDA for the U.S., or the MCC for South Africa) receives all expedited reports it also has the authority to terminate, suspend or require changes to a clinical trial.

5.2 Safety Surveillance During the Study

Subjects will be monitored and safety data collected by way of clinical interviews and examinations, evaluations conducted by study team members, and through reports of laboratory evaluations. Time points and the specific data collected for each of these evaluations are described in protocol appendices.

Through the 28-day period following each vaccination all **adverse events** observed by the investigator (or designee), or reported by the subject spontaneously in response to a direct question, will be medically evaluated and documented in terms of a medical diagnosis on the AE screen.

Through the entire study period all **serious adverse events** observed by the investigator (or designee) or reported by the subject spontaneously or in response to a question will be medically evaluated and documented on the AE screen.

5.3 Definition of Adverse Event

An adverse event (AE) is defined as any unanticipated problem involving risks to study participants or others. An adverse event, therefore, can be any unfavorable or unintended sign, symptom, disease, syndrome, abnormal laboratory finding, or concurrent illness that emerges or worsens relative to the subject's pretreatment baseline, whether or not it is considered to be related to the investigational product.

All conditions that exist prior to administration of the study vaccine (pre-existing conditions) will be recorded in the subject's medical history to establish baseline. Day-to-day fluctuations in pre-existing conditions that do not represent a clinically significant change in the subject's status will not necessarily be reported as adverse events.

Any adverse change from the subject's baseline condition (determined from evaluations conducted at screening/SD0 prior to initial vaccination and at SV4 prior to booster vaccination) that occurs following the administration of the study vaccine will be considered an adverse event. This includes the occurrence of a new adverse event or the worsening of a baseline condition, whether or not considered related to the study vaccine. Intermittent conditions such as headaches may be present on Study Day 0 but may represent an adverse event if the intensity or duration of the event is worse than usual following receipt of study vaccine. Adverse events include but are not limited to: adverse changes from baseline that represent increases in toxicity grade according to the Toxicity Table (see protocol appendices), adverse changes in the general condition of the subject, signs and symptoms noted by the subject, concomitant disease with onset or increased severity after study vaccine administration, and changes in laboratory safety parameters occurring after study vaccine administration.

The reporting period for all adverse events is specified in Section 3. Adverse events will be reported using a recognized medical term or diagnosis that accurately reflects the event. Adverse event evaluations will be reviewed by the principal investigator or by a designated medically qualified practitioner. Adverse event information are to be completed by members of the study

team designated in writing by the principal investigator. The onset and resolution dates of the event and action taken in response to the event will be documented. All adverse events must be followed until resolution is demonstrated, the condition becomes stable or until the end of the study. The resolution date will be recorded as the last date on which the subject experienced the adverse event or, if unresolved by the end of the study, the resolution date will be recorded as "ongoing." Information recorded must be substantiated in the source documents. If an adverse event evolves into a condition that becomes "serious," it will be designated as serious and a SAE Report (SAER) form will be completed.

5.4 Assessing Severity

The safety concepts of "severity" and "seriousness" are distinct concepts (see Section 5.8). Severity refers to a degree of clinical manifestation. "Seriousness" refers to defined outcomes from an adverse event. A severe adverse event is not always serious and a serious adverse event is not always severe.

For all adverse events, the investigator (or designee, who is a healthcare professional; is someone the investigator deems qualified to review adverse event information, to provide a medical evaluation of the event, and to classify the event based upon medical judgment and the severity categories described below) is responsible for assessing the severity of the event and the causal relationship of the event to the study vaccine.

The **severity** of all adverse events, including clinical findings and abnormal laboratory values, will be classified as one of the following grades:

1. **Mild**
2. **Moderate**
3. **Severe**

A Toxicity Table is provided in the protocol appendices for the assessment of severity of specified adverse events. The Toxicity Table Adverse Event Grades do not correlate directly with the classical severity grades of mild, moderate and severe. FOR THE PURPOSES OF RECORDING EVENTS ON THE AE screen, Toxicity Table Grade 1 events will be considered mild in severity, Toxicity Table Grade 2 events will be considered moderate in severity, and both Toxicity Table Grade 3 and 4 events will be considered as severe. In the Toxicity Table certain local reactions such as erythema (redness) and swelling are graded according to size. Laboratory values are graded according to level of deviation from the normal range.

For adverse events not listed in the Toxicity Table determination of severity requires some level of interpretation as outlined below. The degree of incapacity caused by the adverse event and the level of medical intervention required for treatment may be helpful in assessing the overall severity of the adverse event.
For example:

- "Mild" events are generally regarded as noticeable but have no impact on normal activities; they may or may not require over-the-counter treatment managed by the subject.

- "Moderate" events generally have some impact on an individual's normal activities and may require general symptomatic medical intervention by a healthcare professional or by the subject.
- "Severe" adverse events may be incapacitating, leading to suspension of normal daily activities, and would generally require more immediate medical evaluation and intervention by a healthcare professional.

A change in severity of an adverse event will not be recorded as a new adverse event. Only the highest severity level that occurs during the entire period of the adverse event will be recorded with the onset and resolution dates encompassing the entire duration of the event.

5.5 Assessing Causal Relationship (Relatedness)

For all adverse events, the investigator and the sponsor (the local medical monitor) will determine a **causal relationship,** to the study vaccine without knowledge, for blinded studies, of whether MVA85A/AERAS-485, placebo, or active control was administered. A number of factors will be considered in making this assessment, including: 1) the temporal relationship of the event to the administration of the study vaccine 2) whether an alternative etiology has been identified and 3) biological plausibility. The investigator will use the following guidelines to assess the causal relationship of an adverse event to study vaccine:

- **Not Related** to study vaccine (i.e., there is no evidence of a causal relationship; another etiology is known to have caused the adverse event. The alternative etiology should be documented in the subject's study record).
- **Unlikely Related** to study vaccine (i.e., there is less than a reasonable possibility that the adverse event was caused by study vaccine).
- **Possible** relationship to study vaccine (i.e., there is a reasonable possibility that the adverse event was caused by study vaccine. There must be a plausible mechanism for the event to be related to study vaccine. The evidence is inadequate to accept or reject, or favors rejection of, a causal relationship; an association exists between the event and the study vaccine but there may also be an alternative etiology, such as characteristics of the subject's clinical status or underlying condition).
- **Probable** relationship to study vaccine (i.e., it is likely that the adverse event was caused by administration of the study vaccine. The evidence favors acceptance of a causal relationship; an association exists between the event and receipt of the study vaccine and there is a plausible mechanism for the event to be related to the study vaccine, and an alternative etiology is not apparent).
- **Definite** relationship to study vaccine (i.e., the study vaccine is known to be the cause of the adverse event. The evidence establishes a causal relationship; an association exists between the event and receipt of the study vaccine and there is a plausible mechanism for the event to be related to the study vaccine, and causes other than the study vaccine have been ruled out).

The principal investigator and the local medical monitor both determine causality. It is expected that communication and consultation may occur in the assessment of the causality of adverse events. The greatest degree of causal relationship (definite > probable > possible > unlikely

related > not related) determined by either the investigator or local medical monitor after their discussions will determine the ultimate classification of the adverse event. Definite, probable and possible are considered to be related. Not related and unlikely related are considered to be unrelated.

Every effort should be made by the investigator to determine the existence of any pre-existing conditions (e.g., mild nausea or headache on Study Day 0 with onset prior to study vaccination) that must be taken into consideration when assessing causal relationship of an adverse event. Pre-existing conditions should be recorded as baseline history and substantiated by appropriate source documentation. Intermittent conditions such as headaches or menstrual pain may not be present on Study Day 0 but may represent an adverse event if the intensity or duration of the event is worse than usual following study vaccine.

5.6 Definition of Adverse Reaction

An adverse reaction is an adverse event judged to be related to study vaccine (see Section 5.3 for adverse event definition).

Related adverse events (adverse reactions) are defined as those judged by the investigator or local medical monitor to be possibly, probably, or definitely related to study vaccine.

The reporting period for all adverse events and adverse reactions is the 28-day post-vaccination follow-up period. Adverse events and adverse reactions will be reported using a recognized medical term or diagnosis that accurately reflects the event.

5.7 Solicited Adverse Events and Injection Site Reactions

Solicited adverse events are events the subject is specifically asked about. These adverse events are commonly observed soon after receipt of vaccines. For this study, solicited adverse events include: injection reactions, diarrhea, vomiting and others. Solicited adverse events of local injection site reactions (i.e., pain at injection site, redness at injection site, or swelling at injection site) will be considered causally related to study vaccine (adverse reaction).

The reporting period during which *solicited* adverse events will be asked of the subject is the immediate 28 day period following each vaccination. The solicited adverse event reporting period begins with the day of vaccination.

Adverse events and solicited adverse events including assessment of local injection site reactions will be assessed by the investigator for severity, causal relationship to the study vaccine, possible etiologies, and whether the event meets criteria as a serious adverse event (and therefore requires immediate notification to the medical monitor).

Presence of ulceration and/or scarring at the site of injection and axillary lymphadenopathy of the injection arm(s) are considered to be adverse events that are causally related to the study vaccine and are of special interest. Site of injection ulceration (including presence of drainage)

and axillary lymphadenopathy will be actively evaluated during each clinic visit through the end of the study. These events will be recorded on the Adverse Event screen.

In the event that the clinical presentation meets the definition of a serious adverse event, an SAE Report must be completed and the event reported per protocol instructions.

5.8 Subject Diary and Daily Temperature Monitoring

Subjects who receive investigational product will be given, and be instructed in, the operation of a daily adverse event diary and a digital thermometer to be used beginning on the day of each vaccination through 7 days post-vaccination. The daily adverse event diary is a tool to help aid the PI and/or designee to engage in a conversation with the subject about any AEs that may have occurred in-between visits. Entries from the diary card will not be directly recorded onto the electronic case report forms. During scheduled post-vaccination visits, the daily diary will be collected and reviewed by the principal investigator and/or designee at which time any clinical details required for complete understanding of the information recorded will be obtained. Lost diaries or diaries not brought to the scheduled visit will be documented, and the subjects will be queried about the occurrence of clinical events during the 7 days post-vaccination.

Any entry recorded by the subject on the diary card that differs from the opinion of the investigator's evaluation of the event (e.g., the severity level of an event is changed after interviewing the subject) must be explained by notation in the source documentation. Body temperatures below 38º C will not be considered fevers.

5.9 Assessing "Seriousness" and Serious Adverse Events

Seriousness refers to the outcome of an adverse event. Seriousness is determined by both the principal investigator and the local medical monitor. If either principal investigator or local medical monitor determines an event to be serious, it will be classified as such. If any of the following outcomes are present then the adverse event is serious:

- It results in **death** (i.e., the AE caused or led to the fatality). Serious does not describe an event which hypothetically might have caused death if it were more severe.
- It was immediately **life-threatening** (i.e., the AE placed the subject at immediate risk of dying. It does not refer to an event which hypothetically may have led to death if it were more severe).
- It required inpatient **hospitalization** or prolonged hospitalization beyond the expected length of stay. Hospitalizations for scheduled treatments and elective medical/surgical procedures related to a pre-existing condition that did not increase in severity or frequency following receipt of study vaccine, are **not** serious by this criterion. Hospitalization is defined as a hospital admission or an emergency room visit for a period greater than 24 hours.
- It resulted in a persistent or significant **disability/incapacity** (i.e., substantial reduction of the subject's ability to carry out activities of daily living).

- It resulted in a **congenital anomaly or birth defect** (i.e., an adverse finding in a child or fetus of a subject exposed to the study vaccine prior to conception or during pregnancy).
- Other **medically important conditions** that may not result in death, threaten life or require hospitalization (i.e., the AE does not meet any of the above serious criteria) may be considered a serious adverse event when, based on appropriate medical judgment, they may jeopardize the subject and require medical or surgical intervention to prevent one of the serious outcomes listed in these criteria (e.g., allergic bronchospasm requiring intensive treatment in an emergency room or at home; blood dyscrasias or convulsions that do not result in hospitalization, or the development of drug dependency or drug abuse).

A **serious adverse event** is an adverse event meeting the outcome criteria for seriousness regardless of relationship to an administered medicinal product.

5.10 Assessing Expectedness

Expected adverse events are adverse events consistent with the applicable product information provided by the sponsor (the investigator's brochure for an investigational product). The sponsor, in the person of the local medical monitor, determines expectedness. If the assessment is that the adverse event is **expected** no further action is required. If the local medical monitor's assessment is that the adverse event is **unexpected,** then the event may represent a SUSAR (see Sections 5.10 and 5.11).

5.11 Definition of Suspected Unexpected Serious Adverse Reaction (SUSAR)

When an adverse event is judged to be related to an investigational product, such as MVA85A/AERAS-485, and also is judged to be serious and unexpected, it is a SUSAR (suspected unexpected serious adverse reaction) and is subject to expedited reporting.

5.12 Reporting of Serious Adverse Events

***Serious adverse events*, which include SUSARs, are reported to the sponsor and to the World Wide Safety Center (administered by PPD, Inc.) for the entire study period (see protocol appendices). SUSARs are reported even after the trial is over, if the sponsor, local medical monitor or principal investigator become aware of them.** The site will be provided with specific reporting procedures including any supplemental reporting forms to be used. Serious adverse events will be reported using a recognized medical term or diagnosis that accurately reflects the event.

Serious adverse events will be assessed by the investigator and the local medical monitor according to their roles (as described in Sections 5.1.2 and 5.1.4) for severity, causal relationship to the study vaccine, and expectedness. The onset and resolution dates of the event and the action taken in response to the event will be documented. If the event has not resolved by the final study visit, it will be documented as "ongoing", however, follow-up of the SAE must continue until resolved. Information recorded must be substantiated in the source documents.

An SAE Report completed, reviewed and signed by the principal investigator or his/her designee for that event must be emailed or faxed within one business day of the investigative site becoming aware of the event to the local medical monitor, to the World Wide Safety Center at PPD and to the CRO, Triclinium. The initial SAE Report (paper form) should be completed and emailed/faxed even if all information concerning the event is not yet known.

Fatal or life-threatening serious adverse events that the investigator suspects are related to the study vaccine should be telephoned to the local medical monitor immediately upon the investigator's awareness of the event. If the local medical monitor is required by the protocol or chooses to suspend enrollment s/he shall immediately create a written memorandum for record to the study file and telephonically notify the sponsor of this act.

Contact information for all safety personnel are contained in the Team Contact List which will be stored on site in the Site Regulatory Binder and maintained by the study sponsor.

Investigators must not wait to collect additional information to fully document the event before notifying the local and global medical monitors of a serious adverse event. The initial notification should include the following (at minimum):

- Protocol number and name and contact number of the investigator
- Subject ID number (and initials and date of birth, if available)
- Date subject received study vaccine
- Serious adverse event(s) and date of event onset
- Current status of subject

Aeras has authorized the PPD World Wide Safety Center to execute its responsibilities for safety report submission to the appropriate regulatory authorities within specific time periods of being notified of the event (within 7 or 15 calendar days depending the character of the SUSAR); therefore, it is important that the investigator submit additional information requested as soon as it becomes available.

Aeras will notify the Data Monitoring Committee of all SUSARs within three working days of becoming aware of an event and will provide all follow-up information in a timely manner.

5.13 Other Events Requiring Immediate Reporting

The investigator must report the following events by emailing or faxing the appropriate form to the local medical monitor within 24 hours of becoming aware of the event:

- Withdrawal of consent during the study due to an adverse event (Immediately Reportable Event Form)
- Emergency unblinding (Immediately Reportable Event Form)
- Protocol deviation affecting the safety of a subject or involving the vaccination process (Immediately Reportable Event Form)

- Adverse event thought to be an allergic reaction to the study vaccine (Immediately Reportable Event Form, unless event meets SAE criteria)
- Any event that, in the opinion of the investigator, precludes further administration of the study vaccine (Immediately Reportable Event Form, unless meets SAE criteria)
- Pregnancy (Pregnancy Notification Form)

5.14 Adverse Event Treatment, Follow-up, and Outcome

Treatment of any adverse events will be determined by the investigator using his/her best medical judgment and according to current clinical practice guidelines. All applied measures as well as follow-up will be recorded on the appropriate screen.

Adverse events will be considered resolved when the condition returns to normal or returns to the subject's baseline status as established on Study Day 0, or when the condition has stabilized with the expectation that it will remain chronic.

The investigator will continue follow-up on adverse events, including laboratory abnormalities and solicited adverse events, until the event has resolved, is otherwise satisfactorily explained, or the subject completes the study. If not reported earlier, the outcome of adverse events and solicited adverse events will be determined at the end of the specified post-vaccination adverse event reporting period.

Follow-up for serious adverse events must continue until resolution and the outcome reported to Aeras, even if this extends beyond the serious adverse event reporting period (i.e., after the final study visit). For analysis purposes, the outcome for serious adverse events will be determined on the final study visit.

Outcome of all adverse events will be classified as one of the following:

- Resolved
- Resolved with sequelae
- Ongoing
- Death

If at any time after completion of the serious adverse event reporting period (the final study visit) the investigator becomes aware of a serious adverse event that is suspected by the investigator to be related to the study vaccine, the event must be reported to Aeras.

5.15 Follow-up of Subjects Who Become Pregnant

If a subject becomes pregnant during the study, she should be encouraged to continue in the study for safety follow-up and for TB disease according to the protocol. Follow-up should continue for pregnancy outcome including premature terminations, and data are to be included in the safety reports.

The investigator must notify the local medical monitor, World Wide Safety Center and CRO, Triclinium, of the pregnancy immediately (even if already known to have resulted in

spontaneous or elective abortion) by emailing/faxing the Pregnancy Form to the medical monitor. At a minimum, the estimated date of conception, the estimated due date, and the date the subject received the study vaccine should be provided.

If a subject becomes pregnant, she will not have blood drawn as normally mandated by the protocol. The subject will undergo all other evaluations according to the Summary Schedule(s) of Evaluations.

The health status of the mother and child, the date of delivery, and the child's sex, birth weight and multiparity should be reported to the safety monitor after delivery. If delivery occurs before the final study visit, the subject should continue to be followed for SAEs through the final study visit unless withdrawal of consent has occurred. If delivery occurs after the final study visit, the investigator should attempt to maintain contact with the subject to obtain information after delivery.

Pregnancy will not be recorded as an adverse event. However, pregnancies will be recorded in the World Wide Safety Database. If the pregnancy results in a miscarriage, the event (spontaneous abortion) will be reported as an adverse event or a serious adverse event per the investigator's judgment (e.g., if it was a medically important or life-threatening event that meets the definition of a serious adverse event).

A congenital anomaly or birth defect (i.e., an adverse finding in a child or fetus of a subject exposed to the study vaccine before conception or during pregnancy) must be reported as a serious adverse event.

If it is determined after completion of the study that a subject became pregnant during the study, the subject should notify the investigator. The pregnancy must be reported to the local medical monitor and the status of the mother and child after delivery will be obtained and reported, when possible.

6 STOPPING RULES

These rules govern the stopping of study vaccine administration at any time during the study. Stopping rules will include:

- Death in any subject that the DMC determines is RELATED to MVA85A/AERAS-485
- An anaphylactic reaction to MVA85A/AERAS-485 in any subject
- A life-threatening adverse event in any subject that the DMC determines is RELATED MVA85A/AERAS-485
- A serious adverse event that the DMC determines is RELATED to MVA85A/AERAS-485
- A pattern of significant symptoms, physical findings or laboratory abnormalities (adverse events) that, although individually minor, collectively represent a safety concern in the opinion of the investigator or the medical monitor and that the DMC determines are RELATED to MVA85A/AERAS-485

The DMC may permit resumption of study vaccine administration if the study pause was for reasons less severe than those in the DMC stopping rules. The DMC may permit resumption of enrollment if it judges that changes to the study protocol will eliminate or greatly reduce the safety risks specified in the stopping rules. In the absence of study protocol changes the DMC must follow the DMC study stopping rules.

If a decision to resume study enrollment and study vaccine administration is made the DMC will record their judgment in a memorandum to the study file and notify the sponsor, who will then forward the DMC memorandum to the medical monitors and principal investigators. The clinical site will be allowed to resume activities upon receipt of written notification from the sponsor. The appropriate regulatory authority will be informed in writing if the study is stopped and will be informed in writing of the decision by the DMC to resume or discontinue study activities.

7 STATISTICAL CONSIDERATIONS

The planned statistical analyses for this study are outlined below. A detailed statistical analysis plan will be created and finalized prior to database lock and preparation of any blinded preliminary data reviews and for preparation of the final study report (see Section 7.10).

7.1 Subject Populations

The following population definitions will be utilized in specific analyses, as described below. The randomized subject population will consist of all subjects who were randomized into the study. The safety population will consist of all randomized subjects who received at least one dose of study vaccine. The per-protocol (PP) population for efficacy analyses will consist of all randomized subjects that received study vaccine and had no major protocol deviations, to be defined in the statistical analysis plan prior to database lock and study unblinding. The intent-to-treat (ITT) population for efficacy analyses will consist of all randomized subjects; given the proximity of randomization with vaccination, ITT as defined is anticipated to preserve the principles of ITT and to protect the randomization.

7.2 Demographics and Protocol Compliance

Demographic parameters (age, gender, and race/ethnicity) and other baseline characteristics will be summarized by ART strata as defined in Section 3.3 and by treatment group for the safety population and for the PP population. Additional summaries based on all randomized subjects, and by ART strata and by study site, will also be provided.

Listings of randomized subjects who did not receive study vaccine or placebo, subjects who were excluded from the ITT or PP populations will be provided. The number (percentage) of subjects with protocol deviations (to be defined in the statistical analysis plan) will be summarized by treatment group, ART strata, and for all subjects in the ITT population.

7.3 Efficacy Analyses

7.3.1 Case Definition of Tuberculosis

A case of tuberculosis will be defined as any of the following:

TB Case Definition Endpoint #1

Any of the following numerical categories:

1. Isolation of *M. tuberculosis* from any site.
2. Identification of *M. tuberculosis* by an approved molecular diagnostic technique from any site.
3. Histopathology diagnostic for tuberculosis disease (such as caseating granulomas).
4. Choroidal tubercle diagnosed by ophthalmologist.

TB Case Definition Endpoint #2

Any of the following numerical categories:

1. Isolation of *M. tuberculosis* from any site.
2. Identification of *M. tuberculosis* by an approved molecular diagnostic technique from any site.
3. Histopathology diagnostic for tuberculosis disease (such as caseating granulomas).
4. Choroidal tubercle diagnosed by ophthalmologist.
5. A single smear/histology specimen positive for acid-fast bacilli from a normally sterile body site.
6. Two acid-fast smears positive each from a separate collection morphologically consistent with mycobacteria from either pulmonary or gastric sampling that are not found to be non-tuberculous mycobacteria bacteria on culture, and at least one of the following:
   a) a compatible radiographic feature: airspace opacification, cavity, hilar or mediastinal adenopathy, or pleural effusion
   b) a compatible clinical feature, i.e., more than 2 weeks of fever, night sweats, anorexia, cough, or weight loss (at least 5 kg by history or noticeable change in clothing fit); or one or more episodes of hemoptysis.

TB Case Definition Endpoint #3

All individuals who are placed on anti-tuberculosis therapy with the intent of treating tuberculosis, regardless of whether or not they have met the above efficacy endpoint #1 or #2, are considered to have met this exploratory tuberculosis endpoint #3.

The TB endpoint definitions will be applied both to those subjects investigated for TB by the clinical site, and to those subjects who receive a clinical diagnosis of TB disease as part of routine care in other health facilities.

7.3.2 Efficacy of MVA85A/AERAS-485

For the protocol-specified secondary objective of TB disease, **s**ubjects will be evaluated for the development of TB disease throughout the study utilizing TB Case Definition Endpoint #1 as the primary disease endpoint assessment. Less stringent TB case definitions, Endpoint #2 and Endpoint #3, will also be evaluated with no adjustment for multiplicity performed; it is acknowledged that the overall Type I error probability is not controlled in the evaluation of these endpoints.

The incidence of TB disease will be calculated as the number of new cases of TB disease in the PP population with a date of diagnosis from 28 days post-Study Day 0 vaccination through the end of study follow-up (as defined for each subject), divided by the total person-time of observation. Person-time observation will be calculated based on subject date of last contact with the study minus the date of Study Day 0 vaccination; sensitivity analyses will be performed to examine time at risk of TB based on INH treatment.

The primary statistical method to be used in the analysis of TB Case Definition Endpoints #1-3 will be vaccine efficacy (VE), estimated as 1-estimated hazard ratio, based on a Cox regression analysis of time to event for each Endpoint utilizing a model containing treatment group, ART status, and study site, performed on the PP population.

Time (months) to initial TB diagnosis for each Endpoint #1, #2 and #3 from Study Day 0 vaccination in each treatment group will be examined using the Kaplan-Meier estimate of the survival function, based on the PP population. Kaplan-Meier curves will be displayed by ART status and treatment group, over time (months) in study (time from Study Day 0 vaccination) to event. Summaries of median time (in months) to initial TB diagnosis and associated two-sided 95% confidence intervals will also be presented for each case definition endpoint. Time to initial diagnosis for each case definition endpoint will be compared using a two-sided log-rank test, stratified by study site and ART status at baseline. As a supportive analysis, the exact binomial (Clopper-Pearson) method will also be used to estimate VE and its corresponding two-sided 95% confidence interval conditional on the total number of events, and corresponding p-value, for each case definition Endpoint. All summaries will be presented by ART status and treatment group for subjects in the PP population.

Efficacy of MVA85A/AERAS-485 in the prevention of TB disease will be examined for all subjects in the PP population receiving ART at baseline compared to subjects receiving ART at baseline but who receive placebo (secondary objective #3), as described above for secondary objective #1.

In this proof-of-concept study, variables related to HIV status and infection status such as baseline QFN status which may impact development of TB disease may also be included in further stratified Cox proportional hazards regression models, to explore differences between treatment groups in the incidence of TB disease, given proportional hazards assumptions. In addition, the efficacy of MVA85A/AERAS-485 in the prevention of TB disease will be examined for all subjects in the PP population receiving MVA85A/AERAS-485 and isoniazid

preventative therapy at any time during the study following Study Day 0 vaccination compared to subjects receive placebo (secondary objective #4).

Adjustment to time at risk based on start of isoniazid (INH) therapy and examination of cases which occur within 24 months following Study Day 28 (28 days post-Study Day 0 vaccination) will be examined as supportive analyses. Results will be summarized by ART status at baseline, treatment group and study site, as appropriate. Supportive analyses will also be performed based on the ITT population and all randomized subjects' populations. Sensitivity analyses including examination of subjects put on ART during the study and missing data imputations will be explored.

7.3.3 QuantiFERON Conversion Rate

QuantiFERON®-TB Gold In-Tube test results through the end of the study will be summarized by number (percentage) of positive responses by ART status at baseline and treatment group. Descriptive statistics will also be used to summarize quantitative QuantiFERON data (IU/mL interferon results) by ART status at baseline, treatment group, and study visit. Cross-tabulation of the number of subjects with a negative baseline QuantiFERON with subsequent conversion to a positive result post-vaccination through the end of study will be summarized by ART status and treatment group, based on the PP population. The exact binomial (Clopper-Pearson) method will be used to estimate VE and its corresponding two-sided 95% confidence interval conditional on the total number of events, and corresponding p-value.

7.4 Safety Analyses

Safety analyses will be performed using the safety population as defined in Section 7.1. Count (percentage) summaries will be presented by treatment group for all subjects in the safety population.

7.4.1 Adverse Events

The safety profile of MVA85A/AERAS-485 will be evaluated based on the number and percentage of solicited and unsolicited adverse events (including serious adverse events [SAEs]) recorded over the post-vaccination follow-up period for all subjects in the safety population. The number (percentage) of subjects with adverse events will be summarized by MedDRA system organ class (SOC) and preferred term (PT). The proportion of subjects in the control and MVA85A/AERAS-485 treatment groups with at least one adverse event will be compared using Fisher's Exact test; two-sided exact 95% confidence intervals will also be presented for proportions of individual adverse event preferred terms, within treatment group. Supportive summaries by ART status will also be presented. Additional summaries will present the number (percentage) of subjects with adverse events by severity and by relationship to study vaccine; each subject will be counted once per preferred term at the greatest severity or most related state recorded for that term. Separate summaries of the number (percentage) of subjects with solicited adverse events will also be presented. Solicited adverse events will also be summarized by severity and relationship to study vaccine; each subject will be counted once per preferred term at the greatest severity or most related state recorded for that term.

Separate summaries for the number (percentage) of deaths, subjects with serious adverse events, and subjects with HIV-related opportunistic infections or other adverse events will also be presented by ART status at baseline, study site, and treatment group. Listings of subjects who discontinued prematurely due to an adverse event will also be provided.

The number (percentage) of subjects with post-vaccination clinical laboratory values or vital sign values recorded as newly abnormal following study vaccination and meeting toxicity mild criteria (Grade 1) or above as specified in the Toxicity Table (Appendix C) will be tabulated at each post-vaccination time point and overall. Clinical laboratory and vital sign abnormalities will also be reported as adverse events and will be included in the summary of adverse events.

7.4.2 Clinical Laboratory and Vital Sign Parameters

For each clinical laboratory parameter and vital sign parameter pre-specified in the protocol, summary statistics for continuous parameters will be presented by treatment for all pre- and post-vaccination assessments and for change from pre-vaccination to post-vaccination assessments.

7.4.3 CD4+ Lymphocyte Count

Median or geometric mean CD4+ lymphocyte counts and associated two-sided 95% confidence interval will be summarized by ART status at baseline, study site, treatment group, and time point, for subjects in the safety population. Summaries will include change in CD4+ lymphocyte count from pre-vaccination to each post-vaccination time point. Area under the curve will also be used to summarize CD4+ lymphocyte count by treatment group across pre- and post-vaccination time points based on the trapezoidal rule, within strata. Any impact of MVA85A/AERAS-485 vaccination on CD4+ lymphocyte count compared with placebo will be explored using the AUC in a mixed effects model including treatment group, Study Day 0 CD4+ count, age, baseline ART status, and study site. Data for CD4+ lymphocyte count will be log-transformed prior to analysis as appropriate.

7.4.4 HIV-1 Viral Load

HIV-1 viral load (copies/mL) will be summarized using the median and associated 95% confidence interval by ART status at baseline, study site, and treatment group, at each available time point, for subjects in the safety population. Summaries will include change from pre-vaccination to post-vaccination time points. Additional summaries and comparisons of the mean AUC of MVA85A/AERAS-485 and placebo, as described for CD4+ lymphocytes, will also be performed.

7.5 Immunogenicity and Correlates of Protection

7.5.1 Immune Response Determined by ELISPOT Assay

An *ex vivo* IFN-γ ELISPOT assay will be used to assess immune response to vaccine. Descriptive statistics will be used to summarize T cell responses by treatment group and

stimulation antigen, for a subset of subjects (as in Section 3.6.4) in the PP population. Summaries will include the assessment of response at all pre- and post-vaccination immunology time points, as well as change from pre-vaccination to each post-vaccination time point.

7.5.2 Immune Response Determined by Intracellular Cytokine Staining Assay

Immune response to vaccine will also be assessed by determining the percentage of CD4 and CD8 T cells that produce any of 3 cytokines (IFN-γ, TNF-α, and/or IL-2) or a combination of the 3 cytokines simultaneously following stimulation with peptide pools derived from and representing the entire amino acid sequence of the mycobacterial antigen Ag85A. Response will be measured by flow cytometry in the intracellular cytokine staining (ICS) assay.

Median DMSO-subtracted cytokine responses and associated 95% confidence intervals (CI) or other descriptive statistics as appropriate will be used to summarize percentage T cell responses for a subset of subjects (as in Section 3.6.4) in the PP population. Summaries of T cell response will be presented by T cell type (CD4 and CD8) and by stimulation antigen (Ag85A). Summaries will include immune response at all available pre- and post-vaccination immunology time points, and change from pre-vaccination to post-vaccination time points.

7.5.3 Correlates of Protection

Investigations for determining correlates of immune protection to tuberculosis are ongoing and the technical specifications for the assays are unknown at this time. Decisions on the technical aspects for completing the analysis of correlates of immune protection for this study will be made before the study is unblinded. Further information on the planned analysis will be provided in a separate analytic plan, when available.

7.5.4 Other Immunology Analyses

Immunogenicity will be summarized by ART status at baseline, study site, and by treatment group at all time points as collected and as available, based on the PP population. Supportive analyses will also be performed based on the ITT and all randomized subjects populations, as appropriate. No imputation for missing data will be performed. Data will be transformed as appropriate prior to analysis.

7.5.5 Exploratory Immunology Analyses

Analyses of other immunological assessments may be performed to further characterize the immune response to study vaccine. If warranted, results will be summarized by ART status at baseline, study site, and by treatment group at each available time point as described below for each parameter, otherwise results will be provided in subject data listings only.

7.5.6 Tuberculin PPD Skin Test

Results at each available time point will be summarized using count (percentage) summaries and descriptive statistics for the tuberculin PPD skin test (measured in millimeters at the transverse induration), for all subjects in the PP population.

7.6 Sample Size Considerations

The sample size for this study was selected as adequate for a review of the safety profile. Given 325 subjects receiving MVA85A/AERAS-485, the study will have a 90% probability of detecting at least one adverse event which occurs at a rate of 0.71%.

Due to the expected impact of ART on TB disease, an estimated TB disease incidence of between incidence of between 1.5% and 2.0% per year in the control group, based on the TB case definition #1, will be utilized in supportive sample size estimates for efficacy. When the true efficacy is approximately 70%, 325 subjects per treatment group (650 subjects total) will provide 81% power to demonstrate positive efficacy given a rate of 2.0% in the control group per year, or 71% power given a rate of 1.5% in the control group per year. When the true efficacy is approximately 60%, 325 subjects per treatment group will provide 67% power to demonstrate positive efficacy given a rate of 2.0% per year, or 57% power given a rate of 1.5% per year. Calculations are based on a one-sided log-rank test at a significance level of 0.10 and assume completion of enrollment in 21 months, approximately 15-month follow-up period for the last subject enrolled, and a maximum of 36 months for the first subject enrolled. A nominal estimate of approximately 2% mortality and loss to follow-up is assumed per year across ART strata.

7.7 Interim Immunogenicity Review

The sponsor may conduct a review of immune response data prior to database lock after immunogenicity results are available for the first 70 subjects. Aggregate data will be presented at selected time points and will be presented identified only by a blinded label (e.g., ‘Group A’ and ‘Group B’). Review of these data will allow the sponsor to determine if there is evidence of any immune response prior to completion of the trial. No decision cut points or stopping rules will be stipulated, no changes to monitoring practices or study procedures will be implemented following the reviews, and the study will continue following the reviews. No hypothesis testing will be performed.

Data summaries for preliminary immunogenicity review using blinded labels will be prepared by a statistician who is not the study statistician and who is not involved in study design, conduct, or analyses. Immunology technicians will perform all assays in a blinded manner. Data identified for inclusion in the immunogenicity summaries will be cleaned prior to analysis.

7.8 Safety Reviews

The DMC will convene to review safety data (including CD4 counts) as described in Section 5. The DMC will conduct a second unblinded-by-group safety and risk review after the 600th subject enrolled completes one month of follow-up post-vaccination. The DMC will evaluate blinded and unblinded-by-group safety data to determine if a pattern of adverse events related to MVA85A/AERAS-485 or other safety or risk concerns exist. Both blinded and unblinded analyses will be described in a statistical analysis plan, and unblinded reviews will be prepared for the DMC by an unblinded statistician not associated with the study conduct or primary

analyses. All procedures associated with this review, including objectives, data handling, and elements to be included for review will be documented in the DMC minutes.

7.9 Final Study Report

A final study report will be prepared after database lock is completed for all data collected through the final assessment of the study.

Modifications or additions to the analyses described above will be included in the relevant statistical analysis plan(s). Any decisions to deviate from the planned analyses described in the protocol and in the statistical analysis plan will be described in detail in the final study report.

7.10 Computer Methods

Statistical analyses will be performed using SAS® version 9.1 or later under a Windows operating system.

8 DATA COLLECTION, MONITORING, AND RECORD RETENTION

For the purpose of monitoring and auditing the study, source documentation will consist of existing medical records and/or study records developed and maintained by the investigator. Any source document templates provided by Aeras or its designee will serve as supplements to the subject's study record.

Data recorded on source documents will be entered using electronic case report forms (eCRFs) using an Electronic Data Capture (EDC) system provided and approved by Aeras.

The study will be monitored regularly by Aeras or its designee throughout the study period. For studies of unapproved investigational products, all study records (source documents, signed informed consent forms, IRB/IEC correspondence and approval letters, study vaccine management records) will be kept secured for a minimum of two years following the marketing of the investigational product or for 2 years after the discontinuation of the IND (or CTA, etc.). The investigator will ensure that study records are not disposed of or removed from the clinical site without prior notification and approval from Aeras or its designee.

9 HUMAN SUBJECTS

9.1 Ethics and Regulatory Considerations

The study will be conducted in compliance with country specific laws and regulatory requirements. The conduct of the study will also adhere to the GCP principles laid out by the International Conference on Harmonization and the Declaration of Helsinki.

The protocol and informed consent form will be reviewed and approved by the IRB or IEC of each participating clinical site prior to any protocol-specified procedures being conducted. The

investigator will inform the IRB/IEC as to the progress of the study on a regular basis, or at minimum, once a year. Aeras will also have an independent IRB review and approval of the protocol and informed consent form and will keep the IRB informed of the progress of the study.

Written informed consent will be obtained from each subject prior to any protocol-specified procedures being conducted.

To maintain confidentiality, subject identification numbers will be used to identify the subject's laboratory specimens, source documents, CRF, study reports, etc. All study records will be maintained in a secured location. Clinical information will not be released without written permission from the subject except as necessary for monitoring or auditing of the study by Aeras or its designee or applicable regulatory authorities.

9.2 Institutional Review Board or Independent Ethics Committee

All the documents the IRB/IEC may need to fulfill its responsibilities, such as the protocol, protocol amendments, information concerning subject recruitment, payment or compensation procedures, etc., will be submitted to the IRB/IEC by the investigator. The IRB's/IEC's written, unconditional approval of the study protocol and the informed consent form will be in the possession of the investigator/clinical site staff prior to the conduct of any protocol-specified procedures.

Modifications to the protocol may not be implemented without prior written IRB/IEC approval except when necessary to eliminate immediate hazards to the subjects or when the modification involves only logistical or administrative aspects of the study. Such logistical or administrative modifications will be submitted to the IRB/IEC in writing by the investigator, and a copy of the correspondence to verify the submission will be maintained.

The investigator must inform the IRB/IEC of modifications to the informed consent form or any other documents previously submitted for review/approval, of any new information that may adversely affect the safety of the subjects or the conduct of the study, provide an annual update and/or request for re-approval, and advise the IRB/IEC when the study has been completed.

Any documents or forms to be provided to the subject (e.g., information cards, form letters from the investigator), and all forms of study advertising (flyers, brochures, print advertisements, radio or television scripts, etc.) must be approved by Aeras or its designee prior to the clinical site submitting them to the IRB/IEC. Approval from the IRB/IEC must be obtained prior to providing the documents or forms to the subject.

9.3 Informed Consent

The principles of informed consent in the current edition of the Declaration of Helsinki and ICH-GCP should be implemented prior to any protocol-specified procedures being conducted. Informed consent will be documented in writing on a consent form approved by the IRB/IEC.

All relevant information should be provided in both oral and written form in a way that is understandable to the subject. Ample time and opportunity must be given for the subject to inquire about details of the study. The written consent document will embody the elements of informed consent as described in the Declaration of Helsinki and will also comply with local regulations.

The investigator or the investigator's qualified designee will explain the nature of the study and inform the subject that participation is voluntary and that the subject can withdraw at any time. The subject must be informed about the study's purpose including why the subject was selected to participate, study goals, expected benefits and risks, potential risks, and that some potential risks are unforeseeable. The individual must be provided with a description of the procedures and the estimated duration of time required for participation in the study, as well as alternative interventions or courses of treatment, if applicable.

The subject must receive an explanation as to whether any compensation and any medical treatments are available if injury occurs and, if so, what they are, where further information may be obtained, and who to contact in the event of a study-related injury. Individuals must be told who to contact for answers to any questions related to the study.

The subject must be informed that their participation is voluntary and that they are free to withdraw from the study for any reason at any time without penalty or loss of benefits to which they are otherwise entitled. The extent of the confidentiality of subject records must be defined and the subject must be informed that applicable data protection legislation applies.

The subject must be informed that the monitor(s), auditor(s), IRB/IEC members, and the applicable regulatory authorities will be granted direct access to the subject's original study medical records for verification of protocol-specified procedures and/or data, without violating the confidentiality of the subject to the extent permitted by the applicable laws and regulations. The subject must be informed that his/her signature on the informed consent form indicates that he/she has decided to participate in the study, having read and discussed the information presented.

Modifications made by the investigator to an informed consent form template provided to the investigator by Aeras or its designee will be reviewed and approved by Aeras or its designee prior to being submitted to the IRB/IEC.

The original, signed informed consent form for each subject will be maintained by the investigator as part of the subject's study records. A copy of the signed informed consent form will be provided to each subject.

10 STUDY COMPLETION

At the discretion of Aeras, all materials and supplies provided to the investigator will be returned or disposed of in compliance with local regulatory requirements upon authorization from Aeras,

upon study completion. The investigator or designated clinical site staff will notify the IRB/IEC when the study has been completed.

11 PUBLICATIONS

The final study report will be made available to the principal investigator for purposes of publications. The principal investigator and study staff must send all manuscripts, abstracts, and presentations using data from this study to Aeras for review prior to their submission. Aeras reserves the right to delete any part or parts of such materials deemed to be confidential or proprietary.

12 CHANGES IN THE PROTOCOL

The protocol may not be modified without written approval from Aeras. All changes to the protocol must be submitted to the IRB/IEC and must be approved by the IRB/IEC prior to their implementation.

12.1 Changes from Version 1.0 to Version 2.0

Section Number (Title)	Version 1.0	Version 2.0
Title Page; Study Abstract Title	**Change/rationale:** Request of the Medicines Control Council of South Africa to remove reference to countries in the protocol title	
	A Phase II, Proof of Concept, Randomized, Double-blind, Placebo-controlled Study to Evaluate the Protective Efficacy Against TB Disease, Safety, and Immunogenicity of MVA85A/AERAS-485 in Healthy, HIV-infected Adults in South Africa, Senegal and The Gambia	A Phase II, Proof of Concept, Randomized, Double-blind, Placebo-controlled Study to Evaluate the Protective Efficacy Against TB Disease, Safety, and Immunogenicity of MVA85A/AERAS-485 in Healthy, HIV-infected Adults
Study Abstract Design; Section 2.2 Design	**Change/rationale:** The study will be conducted in more than one country; however, The Gambia will no longer be a clinical site. All reference to The Gambia has been removed.	
	This Phase II trial will be conducted as a randomized, double-blind, placebo-controlled, proof-of-concept trial in 1400 HIV-positive adults with no evidence of active TB disease. The study is planned at 3 discrete sites in Africa, 1 in each of South Africa, Senegal, and The Gambia.	This Phase II multi-country trial will be conducted as a randomized, double-blind, placebo-controlled, proof-of-concept trial in 1400 HIV-positive adults with no evidence of active TB disease.
Section 3.2.2 Screening	**Change/rationale:** Screening logs will be maintained by the site to include subject identification, initials and date when the informed consent has been signed. Additional information for subject demographics will only be kept for randomized subjects.	
	Subjects who have signed a consent form will be screened to assess eligibility for the study. A screening log will be maintained by the site that records all subjects who signed an informed consent and entered the screening process. The screening log	Subjects who have signed a consent form will be screened to assess eligibility for the study. A screening log will be maintained by the site that records all subjects who signed an informed consent and entered the screening process.

Section Number (Title)	Version 1.0	Version 2.0
	will document the subject's date of consent, screening number, initials, date of birth, race/ethnicity, and gender. For screened individuals who are subsequently randomized in the study, the date of randomization and the assigned subject identification number will also be recorded.<br><br>For screened individuals who are not subsequently randomized in the study, the screening log will also provide details as to why an individual did not participate in the study. Abnormal results and findings that make the subject ineligible will be discussed with the subject and the subject will be referred for follow-up care with their healthcare provider if necessary.<br><br>Subjects who fail initial screening due to a temporary, reversible cause (e.g., febrile illness) may resume screening once the condition has been resolved.	For screened individuals who are ineligible for the clinical trial, any abnormal results and findings will be discussed with the subject with referral for follow-up care with their healthcare provider as necessary.<br><br>Subjects who fail initial screening due to a temporary, reversible cause (e.g., febrile illness) may resume screening once the condition has resolved.
Section 4.3 Receipt and Storage	**Change/rationale:** Regulatory guidelines for labeling investigational product for clinical trials.	
	"For Aeras C-030-485 Study Only".	"For Clinical Trial Use Only".
Section 5.7 Solicited Adverse Events and Injection Site Reactions	**Change/rationale:** Reference to infants and parent/guardian's were inadvertently left in the protocol; information on diary card and temperature monitoring moved to new Section 5.8.	
	**The reporting period during which *solicited* adverse events will be asked of the subject is the immediate 28 day period following vaccination.** The solicited adverse event reporting period begins with the day of vaccination. All infant's parents/guardians will be provided a diary card to record information regarding occurrences of these specific events. Solicited adverse events and temperature will be recorded by the infant's parents/guardians in a diary beginning on the day of vaccination through 7 days post-vaccination. The diary card, when completed, will serve as source documentation for reporting on the Adverse Event screen. For the remainder of the solicited adverse event collection period, clinic staff will query the parent/guardian about events based on the diary.	**The reporting period during which *solicited* adverse events will be asked of the subject is the immediate 28 day period following vaccination.** The solicited adverse event reporting period begins with the day of vaccination.

<table>
<tr><th>Section Number (Title)</th><th>Version 1.0</th><th>Version 2.0</th></tr>
<tr><td rowspan="2">Section 5.8 Subject Diary and Daily Temperature Monitoring</td><td colspan="2">Change/rationale: Description of diary card distribution, collection and assessment was inadvertently omitted from protocol FV1.0.</td></tr>
<tr><td>N/A</td><td>Subjects who receive study vaccine will be given, and be instructed in, the operation of a daily adverse event diary and a digital thermometer to be used beginning on the day of vaccination through 7 days post-vaccination. The daily adverse event diary is a tool to help aid the PI and/or designee to engage in a conversation with the subject about any AEs that may have occurred in-between visits. Entries from the diary card will not be directly recorded onto the case report forms. During scheduled post-vaccination visits, the daily diary will be collected and reviewed by the principal investigator and/or designee at which time any clinical details required for complete understanding of the information recorded will be obtained. Diaries not brought to the scheduled visit should be obtained before adverse event assessment can be performed and events discussed with the PI and/or designee. Lost diaries will be reconstructed as best as possible on a new diary card by the subject from memory on the closest clinic visit and labeled as a reconstructed diary.

Any entry recorded by the subject on the diary card that differs from the opinion of the investigator’s evaluation of the event (e.g., the severity level of an event is changed after interviewing the subject) must be explained by notation in the source documentation. Body temperatures below 38º C will not be considered fevers.</td></tr>
<tr><td rowspan="2">Appendix A Detailed Description of Study Visits</td><td colspan="2">Change/rationale: Clarification for blood collected for QuantiFERON®-TB Gold In-Tube must be collected prior to Tuberculin PPD skin test being administered.</td></tr>
<tr><td>N/A</td><td>QuantiFERON®-TB Gold In-Tube (NOTE: Blood sample must collected <u>prior</u> to Tuberculin PPD skin test)</td></tr>
<tr><td>Appendix C Toxicity Table</td><td colspan="2">Change/rationale: “Hemoglobin (Female) change from baseline” and “Hemoglobin (male) change from baseline” were inadvertently left in the protocol. The absolute values for hemoglobin will be used to determine if results meet toxicity grades.</td></tr>
<tr><td rowspan="2">Appendix D Phlebotomy Volumes</td><td colspan="2">Change/rationale: Clarification that blood collection volumes listed in the protocol are for the maximum amount collected during a clinic visit.</td></tr>
<tr><td>Estimated total blood collected per protocol</td><td>Estimated maximum total blood collected per protocol</td></tr>
<tr><td rowspan="2">Appendix D Phlebotomy Volumes</td><td colspan="2">Change/rationale: The overall volume for sample collected for immunology cells and plasma remains the same, however, a clarification for the type of tubes used was added.</td></tr>
<tr><td>Immunology Cells and Plasma</td><td>Immunology Cells and Plasma</td></tr>
</table>

Section Number (Title)	Version 1.0	Version 2.0
	8.0 ml BD CPT™ (sodium heparin) (5)	8.0 ml BD CPT™ (4) and 4.0 ml sodium heparin tube (1)
Throughout	Minor editorial changes were made for clarity and consistency.	

12.2 Changes from Version 2.0 to Version 3.0

Section Number (Title)	Version 2.0	Version 3.0
Study Abstract	Updated as appropriate to be consistent with protocol changes described below.	
Section 1.4 Clinical Experience with MVA85A/AERAS-485	Updated with more current clinical experience, in particular with recent immunologic response data in HIV-infected adults receiving a booster vaccination with MVA85A/AERAS-485.	
Section 2.1 Objectives	**Change/rationale:** Added another secondary objective to analyze vaccine efficacy in subjects receiving ART at study entry, a new study sub-population added in this protocol amendment	
	Not present	3. To evaluate the efficacy of MVA85A/AERAS 485 in the prevention of TB disease in subjects receiving ART at baseline compared to subjects receiving ART at baseline but who receive placebo.
Section 2.2 Design	**Change/rationale:** Updated to include stratification at randomization for the two study sub-populations (ART+ or ART- at randomization) and the addition of a booster vaccination	
	Subjects will be stratified by baseline QFN, PPD and prior TB treatment status and then randomized in a ratio of 1:1 to receive either MVA85A/AERAS-485 at 1 x 108pfu or placebo (Candin)…Subjects will receive a single dose of MVA85A/AERAS-485 or placebo on Study Day 0, administered by intradermal injection.	Subjects will be stratified at the time of randomization by whether or not they are currently receiving ART and then randomized in a ratio of 1:1 to receive either MVA85A/AERAS-485 at 1 x 108pfu or placebo (Candin),..Randomization of each group will be capped so that half the subjects randomized will be receiving ART at randomization (n=700) and half will not (n=700). Subjects will receive an intradermal injection of MVA85A/AERAS-485 or placebo on Study Day 0, followed 6-9 months later by a booster injection of MVA85A/AERAS-485 or placebo.
Section 3.1 Schedule of Subject Evaluations	The Schedule of Evaluations has been revised to 1) include a booster vaccination at 6-9 months after the initial vaccination, 2) allow a screening interval of 45 days prior to randomization, 3) a pregnancy test before the booster vaccination, 4) add safety evaluations before and after the booster vaccination, 5) remove the injection site photographs, and 6) HIV viral load determinations at SD84, SD336 and 504 removed; HIV viral load at 6 months post-boost added.	
Section 3.2.3 Inclusion Criteria	**Change/rationale:** Updated to 1) add eligibility criterion for subjects receiving ART at randomization and 2) extend screening period from 30 to 45 days	
	7. Has 2 CD4+ lymphocyte count test results >350 cells/mm^3, performed at least 4 weeks apart, one performed within 6	7. If not receiving ART at the time of randomization, must have 2 CD4+ lymphocyte count test results >350

Section Number (Title)	Version 2.0	Version 3.0
	months prior to randomization and one within 30 days prior to randomization<br>9. Has either<br>a. a negative QuantiFERON-TB Gold In-Tube test result and tuberculin PPD skin test ≤5 mm induration within 30 days prior to randomization or<br>b. a positive QuantiFERON-TB Gold In-Tube test result and/or tuberculin PPD skin test >5 mm and has completed at least 6 months of isoniazid preventive therapy prior to randomization…	cells/mm^3, performed at least 4 weeks apart, one performed within 6 months prior to randomization and one within 45 days prior to randomization<br>8. If receiving antiretroviral therapies (ART) at the time of randomization, must have 2 CD4+ lymphocyte count test results >300 cells/mm^3, performed at least 4 weeks apart, one performed within 6 months prior to randomization and one within 45 days prior to randomization<br>a. Subjects on ART must have been receiving ART for at least 6 months prior to randomization and must have an undetectable HIV viral load within 45 days prior to randomization<br>b. Women who received ART as part of the PMTCT program must have completed therapy at least 2 months prior to randomization<br>9. Has either<br>a. a negative QuantiFERON-TB Gold In-Tube test result and tuberculin PPD skin test ≤5 mm induration within 45 days prior to randomization or<br>b. a positive QuantiFERON-TB Gold In-Tube test result and/or tuberculin PPD skin test >5 mm and has completed at least 5 months of isoniazid preventive therapy within 3 years prior to randomization…
Section 3.2.4 Exclusion Criteria	**Change/rationale:** Updated to remove eligibility criterion that excluded subjects receiving ART	
	6. Has received antiretroviral therapy (ART) in the two months prior to study entry (women who have received ART as part of the PMTCT program and completed this more than 2 months prior to randomization ARE eligible)	Not present
Section 3.3 Study Randomization	**Change/rationale:** A stratification variable was modified for the enrollment of subjects receiving ART at randomization	
	Due to the association between TB infection, TB disease, and prior TB treatment, randomization will be stratified by QFN/PPD and prior TB treatment status. Eligible subjects will be assigned to one of two strata: (1) positive QFN and/or tuberculin PPD skin test >5 mm induration at screening and either INH	Subjects will be stratified based on whether or not they are receiving ART at the time of randomization. Randomization of each group will be capped so that half the subjects randomized will be receiving ART at randomization (n=700) and half will not (n=700).

Section Number (Title)	Version 2.0	Version 3.0
	prophylaxis or successful completion of treatment for active TB less than 3 years previously; or (2) negative QFN and tuberculin PPD skin test ≤5 mm induration at screening.	
Section 3.5 Study Vaccine Administration	**Change/rationale:** Added requirement for pregnancy screening and subject health screening prior to administration of booster vaccine.	
	Not present	Prior to booster vaccination, the clinical investigator will assess each subject's current health status and medical history to determine if there are any medical contraindications to receiving booster vaccinations. All females who are physically capable of pregnancy must have a negative urine βHCG within 2 days prior to the booster vaccination. Subjects will receive the same study vaccine/placebo for the booster vaccination as they received for their initial vaccination based on the study randomization schedule.
Section 3.6.2 Clinical Assessments and Laboratory Tests	**Change/rationale:** Description of the frequency of scheduled study visits has been updated	
	An examination of the injection site will be performed 60 minutes after study vaccine/placebo administration (including an injection site photograph immediately after injection on Study Day 0) and at Study Days 7 and 28. Subjects will have CD4+ lymphocyte counts done and vital signs recorded at Study Days 0, 7, 28, 84, 168, 252, 336 and at all subsequent study visits. Subjects will also have tests done for HIV-1 viral load results done prior to injection on Study Day 0 and then again on Study Day 7, 28, 84, 168, 336 and at Study Months 18 and 24, repeating every 6 months thereafter.<br><br>Blood for hematology and serum chemistry will be collected at screening and on Study Days 7 and 28.	An examination of the injection site will be performed 60 minutes after each study vaccine/placebo administration and at 7 and 28 days after each vaccination. Subjects will have CD4+ lymphocyte counts done and vital signs recorded prior to each vaccination, at 7, 28, 84 days after each vaccination, and at all subsequent study visits. Subjects will also have tests done for HIV-1 viral load results done prior to each vaccination, at 7 and 28 days after each vaccination, then at 6 months post-boost, and at the end of the study follow-up.<br><br>Blood for hematology and serum chemistry will be collected at screening and prior to booster vaccination and 7 and 28 days after each vaccination.
Section 3.6.4.1 Real Time Evaluation of Vaccine Immunogenicity	**Change/rationale:** The number of subjects who receive 'real-time' immunologic evaluations has been reduced. The description of the schedule for collecting these samples was also updated to reduce the number of collections and to account for the booster vaccination	
	There are three assays which will be used to determine vaccine immunogenicity on the first 10% of the subjects enrolled (a total of 140 subjects, across QFN/PPD strata as defined in Section 3.3, with a minimum of 30 subjects at each clinical site). Samples will be obtained from these	There are three assays which will be used to determine vaccine immunogenicity on the first 5% of the subjects enrolled (a total of 70 subjects, across ART+/ART- strata as defined in Section 3.3, with a minimum of 30 subjects at each clinical site). Samples will be obtained from these 70

Section Number (Title)	Version 2.0	Version 3.0
	140 subjects at the following five time points: 0 (baseline day of vaccination), 7 days, 28 days, 1 year and 2 years post vaccination.	subjects prior to each vaccination, and at 7 and 28 days after each vaccination.
Section 5.1.5 Data Monitoring Committee	**Change/rationale:** Update to DMC review schedule to clarify when planned reviews will occur.	
	After the 200th subject enrolled has completed one month of follow-up post-vaccination, a DMC will evaluate blinded and unblinded-by-group safety data …The DMC will conduct a second unblinded-by-group safety and risk review after the 600th subject enrolled has completed one month of follow-up post-vaccination.	After the 200th subject enrolled has completed one month of follow-up post-primary vaccination, a DMC will evaluate blinded and unblinded-by-group safety data …The DMC will conduct a second unblinded-by-group safety and risk review after the 600th subject enrolled has completed one month of follow-up post-primary vaccination.
Section 7. Statistical Considerations	This section was updated because of the addition of subjects receiving ART by 1) using subjects' ART status at randomization to summarize and analyze the data and 2) describing the effect on the sample size of adding subjects who are receiving ART. Also, the number of TB events specified in the event-driven design section of the sample size considerations has been updated to reflect the event fractions for a VE of 60%. The number of TB events previously specified was based on an equal proportion of events in the two treatment groups.	
Section 9. Human Subjects	**Change/rationale:** References to the US Code of Federal Regulations were removed	
	The study will be conducted according to the Declaration of Helsinki, ICH-GCP, Protection of Human Subjects (21 CFR 50), Institutional Review Boards (21 CFR 56), Obligations of Clinical Investigators (21 CFR 312), and local regulatory requirements…<br>The principles of informed consent in the current edition of the Declaration of Helsinki and 21 CFR 50.25 should be implemented prior to any protocol-specified procedures being conducted.	The study will be conducted in compliance with country specific laws and regulatory requirements. The conduct of the study will also adhere to the GCP principles laid out by the International Conference on Harmonization and the Declaration of Helsinki…<br>The study will be conducted in compliance with country specific laws and regulatory requirements. The conduct of the study will also adhere to the GCP principles laid out by the International Conference on Harmonization and the Declaration of Helsinki.
Appendix A Detailed Description of Study Visits	The visit schedule and the description of the procedures at each visit were updated to be consistent with the protocol amendments described above.	
Throughout	Minor editorial changes were made for clarity and consistency.	

12.3 Changes from Version 3.0 to Version 4.0

Section Number (Title)	Version 3.0	Version 4.0
Section 3.6.4.1 Real Time Evaluation of Vaccine Immunogenicity	**Change/rationale:** The number of exploratory assays planned for the first 70 subjects randomized has been reduced; WBA-ICS assays are no longer planned at 28 days after each vaccination.	
	2. Whole blood from all 70 subjects will be frozen for use in the WB ICS assay,	2. Whole blood from all 70 subjects collected only prior to each vaccination

Section Number (Title)	Version 3.0	Version 4.0
	which will be performed centrally.	and 7 days after each vaccination will be frozen for use in the WB ICS assay, which will be performed centrally.
Table 3 Summary of Real Time Immunology Evaluations	**Change/rationale:** Table has been updated to reflect the change described above.	
Section 3.6.4.2 Immune Correlate Samples	**Change/rationale:** The number of exploratory assays planned has been reduced. Except for the first 70 subjects randomized, no further blood will be collected for WBA-ICS assays.	
	Three different samples will be collected on the specified study days (Table 3-1) and stored for all subjects randomized:<br><br>1. PBMC sample<br>2. Serum/plasma sample<br>3. WB ICS sample<br><br>When the trial is completed and unblinded, TB cases and matched controls will be identified and selected immunological analyses will be conducted. Such analyses, to be conducted at Oxford University, Aeras, and the clinical trial sites, may include polychromatic flow cytometry to determine the functionality and phenotype of the antigen specific T cells, CFSE proliferative assays, microarray and RT-PCR analysis, and whole blood intracellular cytokine staining to detect CD8+ T cells.	Two different samples will be collected on the specified study days (Tables 3-1 and 3-2) and stored for all subjects randomized (except for the 70 participants mentioned above):<br><br>1. PBMC sample<br>2. Serum/plasma sample<br><br>When the trial is completed and unblinded, TB cases and matched controls will be identified and selected immunological analyses will be conducted. Such analyses, to be conducted at Oxford University, Aeras, and the clinical trial sites, may include polychromatic flow cytometry to determine the functionality and phenotype of the antigen specific T cells, CFSE proliferative assays, microarray and RT-PCR analysis.
Table 3-1, Summary Schedule of Subject Evaluations: Randomization/initial vaccination (SD0) through Booster Vaccination (V4)	**Change/rationale:** Cumulative and per visit volumes of blood collected have been re-calculated to reflect the reduction in exploratory immunologic assays and blood collection for HLA testing on Study Day 0	
Section 4.1 Supplies	**Change/rationale:** The description of the study vaccine has been updated with the final target concentration of MVA85A.	
	MVA85A/AERAS-485: formulated in Tris buffer (10 mM Tris, 140 mM NaCl, pH 7.7) at a target concentration of between 8 x 10^8 and 2 x 10^9 plaque-forming units (pfu)/mL. The dose of study vaccine to be administered will be 1 x10^8 pfu.	MVA85A/AERAS-485: formulated in Tris buffer (10 mM Tris, 140 mM NaCl, pH 7.7) at a target concentration of between 4 x 10^8 and 1 x 10^9 plaque-forming units (pfu)/mL. The dose of study vaccine to be administered will be 1 x10^8 pfu.
Section 5.8 Subject Diary and Daily Temperature Monitoring	**Change/rationale:** Lost participant diaries will no longer be reconstructed by the participants. Instead, clinic staff will interview the participants about any adverse events as they do at later follow-up visits	
	Diaries not brought to the scheduled visit should be obtained before adverse event assessment can be performed and events	Lost diaries or diaries not brought to the scheduled visit will be documented, and the subjects will be queried about the

Section Number (Title)	Version 3.0	Version 4.0
	discussed with the PI and/or designee. Lost diaries will be reconstructed as best as possible on a new diary card by the subject from memory on the closest clinic visit and labeled as a reconstructed diary.	occurrence of clinical events during the 7 days post-vaccination.
Section 5.12 Reporting of Serious Adverse Events	**Change/rationale:** Only suspected unexpected serious adverse reactions will be immediately reported to the data monitoring committee; other SAEs will be summarized and reviewed by the data monitoring committee at scheduled meetings.	
	Aeras will notify the Data Monitoring Committee of all SAEs within three working days of becoming aware of an event and will provide all follow-up information in a timely manner.	Aeras will notify the Data Monitoring Committee of all SUSARs within three working days of becoming aware of an event and will provide all follow-up information in a timely manner.
Section 7.5.3 HLA Alleles	**Change/rationale:** Exploratory analyses based on HLA typing have been removed.	
	HLA alleles will be determined for possible retrospective analysis of differences in immune response. Summaries of alleles present will be presented by locus and allele and will be summarized in subject data listings only.	Removed
APPENDIX A Detailed Description of Study Visits	**Change/rationale:** Exploratory analyses based on HLA typing have been removed.	
	Blood collection for HLA testing on Study Day 0 was removed	Removed
APPENDIX D Phlebotomy Volumes	**Change/rationale:** Cumulative and per visit volumes of blood collected have been re-calculated to reflect the changes described above and make some corrections from previous protocol versions.	
APPENDIX E Subject Diary (template	**Change/rationale:** The diary has been revised to make it easier for participants to complete.	
Throughout	The name of the sponsor was changed from "Aeras Global TB Vaccine Foundation" to "Aeras." Minor editorial changes were made for clarity and consistency.	

12.4 Changes from Version 4.0 to Version 5.0

Section Number (Title)	Version 4.0	Version 5.0
Section 3.2.3, “Inclusion Criteria”, criterion #10	**Change/rationale:** The list of acceptable methods of birth control has been revised because it included methods no longer recommended for women receiving anti-retroviral medications and to allow clinicians flexibility in choosing contraceptives based on current best practices and recommendations. Also, the length of time women are required to avoid pregnancy has been reduced to be consistent with previous studies of MVA85A/AERAS-485 in HIV-infected participants.	
	Women physically capable of pregnancy (not sterilized and still menstruating or within 1 year of the last menses if menopausal) in sexual relationships with men must avoid pregnancy by using an acceptable method of avoiding pregnancy from 28 days prior to administration of the study vaccine through the end of the study. Acceptable methods of avoiding	Women physically capable of pregnancy (not sterilized and still menstruating or within 1 year of the last menses if menopausal) in sexual relationships with men must avoid pregnancy by using an acceptable method of avoiding pregnancy from 28 days prior to administration of the study vaccine through 6 months after the last study vaccination. Acceptable

Section Number (Title)	Version 4.0	Version 5.0
	pregnancy include a sterile sexual partner, sexual abstinence (not engaging in sexual intercourse), hormonal contraceptives (oral, injection, transdermal patch, or implant), vaginal ring, intrauterine device (IUD), or the use of a condom or a diaphragm combined with spermicide.	methods of avoiding pregnancy include a sterile sexual partner, sexual abstinence (not engaging in sexual intercourse), and any contraceptive method deemed clinically suitable by the trial clinician taking into account ART status.
Appendix E, "Subject Diary (Template)"	**Change/rationale:** The diary card template has been modified to separate the original category of "Nausea or Vomiting" into separate two categories ("Nausea" and "Vomiting) to facilitate the coding and reporting of the events.	

12.5 Changes from Version 5.0 to Version 6.0

Section Number (Title)	Version 5.0	Version 6.0
Study Abstract	Updated as appropriate to be consistent with protocol changes described below.	
Section 1.4, "Clinical Experience with MVA85A/AERAS-485"	**Change/rationale:** Section updated with the results of a recently completed Phase 2B study infants, and for consistency with safety data presented in the most recent version of the MVA85A/AERAS-485 Investigator's Brochure.	
Section 2.1, "Objectives"	**Change/rationale:** Because of the results of the Phase 2B infant study, in which additional protection against TB disease or *M. tuberculosis* infection in MVA85A/AERAS-485-vaccinated infants compared to infants who received BCG alone was not demonstrated, the study has been modified to be a proof-of-concept study with a smaller sample size and shorter follow-up. The objectives have, therefore, been modified so that vaccine efficacy is now a secondary objective and the primary objective is to evaluate vaccine safety.	
	**Primary Objective**<br>The primary objective of this study is to evaluate the efficacy of MVA85A/AERAS 485 in the prevention of TB disease compared to control subjects who receive placebo in HIV infected, African adult subjects without active TB disease.<br>**Secondary Objectives**<br>The secondary objectives of this study are:<br>1. To evaluate the safety of MVA85A/AERAS-485 compared to placebo…	**Primary Objective**<br>The primary objective of this study is to evaluate the safety of MVA85A/AERAS-485 compared to placebo.<br>**Secondary Objectives**<br>The secondary objectives of this study are:<br>1. To evaluate the efficacy of MVA85A/AERAS 485 in the prevention of TB disease compared to control subjects who receive placebo in HIV infected, African adult subjects without active TB disease…
Section 2.2, "Design"	**Change/rationale:** The study has been redesigned as a safety study with a proof-of-concept secondary objective and the sample size and length of follow-up have been reduced. Also, distribution of ART+/ART- subjects at enrollment has been modified to reflect the increased use of ART in the management of HIV+ persons.	
	This Phase II multi-country trial will be conducted as a randomized, double-blind, placebo-controlled proof-of-concept trial in 1400 HIV-positive adults with no evidence of active TB disease…<br>Randomization of each group will be capped so that half the subjects	This Phase II multi-country trial will be conducted as a randomized, double-blind, placebo-controlled proof-of-concept trial in 650 HIV-positive adults with no evidence of active TB disease…<br>Randomization of each group will be capped so that at least 50% of the subjects

Section Number (Title)	Version 5.0	Version 6.0
	randomized will be receiving ART at randomization (n=700) and half will not (n=700)…The initial follow-up period for each subject will be 24 months, during which subjects will be followed for safety, clinical signs and symptoms of TB, and immunogenicity. Subjects will continue to be followed every 3 months after Study Month 24 for a minimum of 2 years after the last subject is enrolled. Duration of any further follow-up is contingent upon accrual of TB cases. The total enrollment period for this study is expected to be between 20 and 24 months.	randomized will be receiving ART at randomization…The minimum follow-up period for each subject will be 6 months after their last vaccination, during which subjects will be followed for safety, clinical signs and symptoms of TB, and immunogenicity. All subjects will continue to be followed every 3 months until the last subject enrolled has been followed for 6 months after their last vaccination.
Table 3.2, "Summary Schedule of Subject Evaluations: Post Booster Vaccination through the End of Study Follow-up"	**Change/rationale:** CD4 counts will be collected at 6 months after the final booster vaccination rather than every 3 months, HIV viral load will only be collected at 6 months after the final booster, and blood draw volumes have been reduced accordingly.	
Section 3.5, "Study Vaccine Administration"	**Change/rationale:** New instructions added	
	Not present	*The Sponsor should be contacted whenever the investigator believes that the vaccinations for a subject will not be given in the protocol-specified timeframes.*
Section 3.6.2, "Clinical Assessments and Laboratory Tests"	**Change/rationale:** CD4 counts will be collected at 6 months after the final booster vaccination rather than every 3 months and HIV viral load will only be collected at 6 months after the final booster	
	…Subjects will have CD4+ lymphocyte counts done and vital signs recorded prior to each vaccination, at 7, 28, 84 days after each vaccination, and at all subsequent study visits. Subjects will also have tests done for HIV-1 viral load results done prior to each vaccination, at 7 and 28 days after each vaccination, then at 6 months post-boost, and at the end of the study follow-up.	…Subjects will have CD4+ lymphocyte counts done prior to each vaccination, at 7, 28, and 84 days after each vaccination and then at 6 months post-boost. Vital signs will be recorded prior to each vaccination, at 7, 28, 84 days after each vaccination, and at all subsequent study visits. Subjects will also have tests done for HIV-1 viral load results done prior to each vaccination, at 7 and 28 days after each vaccination, and then at 6 months post-boost.
Section 3.6.3, "Evaluation of Tuberculosis/Mortality"	**Change/rationale:** The criteria for initiating a TB work-up have been modified. The evaluations for a TB work-up have been modified to allow the work-up to be completed in one day under certain circumstances and both PA and lateral x-rays are required.	
	**Criteria for Pulmonary TB workup**<br>• Cough > 1 week<br>• Fever > 1 week<br>• Night sweats<br>• Weight loss of > 1.5 kg in the past 4 weeks<br>• Pleuritic chest pain<br>• Hemoptysis<br>• Shortness of breath on exertion	**Criteria for Pulmonary TB workup**<br>• Cough > 1 week<br>• Fever > 1 week<br>• Drenching night sweats<br>• Unintentional weight loss of > 3.0 kg in the past 4 weeks or since last visit<br>• Pleuritic chest pain<br>• Hemoptysis<br>• Shortness of breath

Section Number (Title)	Version 5.0	Version 6.0
	**Pulmonary TB Workup**<br>1st visit for any of above symptoms:<br>•PA Chest X-Ray (or schedule for next day) (lateral CXR upon clinical discretion)<br>•Instructions on deep coughing<br>•1st specimen collected (spot)<br>oSputum induction (if cough is inadequate)<br>•Provide specimen cup for morning sputum specimen<br><br>2nd visit (following day)<br>•Retrieve morning sputum specimen (2nd specimen)<br>•3rd specimen collected (spot)<br>oSputum induction (if cough is inadequate)	**Pulmonary TB Workup**<br>1st visit for any of above symptoms:<br>• PA and lateral Chest X-Ray (or schedule for 2nd visit)<br>• Instructions on deep coughing<br>• 1st sputum specimen collected (spot)<br>○ 2nd sputum collected by cough induction<br>• Provide specimen cup for morning sputum specimen (if sputum induction is unsuccessful or not done, and subject scheduled for a 2nd visit)<br><br>2nd visit (within 5 days of 1st visit; to be completed only if sputum induction at 1st visit is unsuccessful or not done, or for chest x-rays)<br>• Retrieve morning sputum specimen (2nd specimen)<br>• 3rd specimen collected (spot)<br>Sputum induction (if cough is inadequate)
Section 3.6.5, “Concomitant Medications”	**Change/rationale:** New instructions added	
	The collection period for concomitant medications associated with the treatment of serious adverse events (SAE) will be Study Day 0 through the end of the study.	The collection period for concomitant medications associated with the treatment of serious adverse events (SAE) and anti-retrovirals for the treatment of HIV will be Study Day 0 through the end of the study.
Section 4.3, “Receipt and Storage”	**Change/rationale:** Instructions for storing MVA85A/AERAS-485 have been modified based on current stability data.	
	MVA85A/AERAS-485 will be stored frozen at -20° C or colder in a frost-free or frostless freezer.	**MVA85A/AERAS-485** will be stored frozen at -40° C or colder in a frost-free or frostless freezer. Vaccine potency is not affected by long-term storage at temperatures up to -20°C, but viral titers may be affected and, therefore, the vaccine should not be kept at -20°C for more than 72 hours.
Section 5.1.3, “Local Medical Monitor,” and Section 5.1.4, “Global Medical Monitor”	**Change/rationale:** Descriptions of both medical monitors have been updated and the responsibilities of the global medical monitor have been modified.	
Section 5.3, “Definition of Adverse Event”	**Change/rationale:** The baseline for judging AEs was modified to account for the long interval between vaccinations.	
	Any adverse change from the subject’s baseline condition (determined from screening evaluations conducted to confirm study eligibility) that occurs following the administration of the study vaccine will be considered an adverse event.	Any adverse change from the subject’s baseline condition (determined from evaluations conducted at screening/SD0 prior to initial vaccination and at SV4 prior to booster vaccination) that occurs following the administration of the study vaccine will be considered an adverse event.

<table>
<tr><th>Section Number (Title)</th><th>Version 5.0</th><th>Version 6.0</th></tr>
<tr><td>Section 5.16, “Long-term Safety Surveillance”</td><td colspan="2">Change/rationale: This section of the protocol was removed, as subjects will no longer be required to have extended follow-up on the Registry study. The Sponsor may implement the Registry study again for late-stage protocols for which the protocol goal is vaccine licensure.</td></tr>
<tr><td>Section 7, “Statistical Considerations” and associated subsections</td><td colspan="2">Change/rationale: In addition to the changes described below specifically for Sections 7.1 and 7.3.1, Section 7 was modified throughout to reflect the changes described above, including 1) designating vaccine efficacy as a secondary objective and safety as a primary objective, 2) discussion of the safety and vaccine efficacy findings attainable with the reduced study size, and 3) modified data analyses based on the Sponsor’s current practices for changes in CD4 counts, HIV viral load and immune responses.</td></tr>
<tr><td rowspan="2">Section 7.1, “Subject Populations”</td><td colspan="2">Change/rationale: The PP population is no longer defined as having received both vaccinations.</td></tr>
<tr><td>The per-protocol (PP) population for efficacy analyses will consist of all randomized subjects that received both doses of study vaccine and had no major protocol deviations.</td><td>The per-protocol (PP) population for efficacy analyses will consist of all randomized subjects that received study vaccine and had no major protocol deviations, to be defined in the statistical analysis plan prior to database lock and study unblinding.</td></tr>
<tr><td rowspan="2">Section 7.3.1, “Case Definition of Tuberculosis”</td><td colspan="2">Change/rationale: TB Case Definition #1 has been modified to be more stringent</td></tr>
<tr><td>5. A single smear/histology specimen positive for acid-fast bacilli from a normally sterile body site.
6. Two acid-fast smears positive each from a separate collection morphologically consistent with mycobacteria from either pulmonary or gastric sampling that are not found to be non-tuberculous mycobacteria bacteria on culture,
and
a compatible radiographic feature: upper lobe airspace opacification or cavity; middle or lower lobe airspace opacification with ipsilateral hilar adenopathy; miliary pattern; bilateral hilar or mediastinal adenopathy; or pleural effusion,
and
a compatible clinical feature, i.e., more than 2 weeks of fever, night sweats, anorexia, cough, or weight loss (at least 5 kg by history or noticeable change in clothing fit); or one or more episodes of hemoptysis.</td><td>Criteria removed</td></tr>
<tr><td>Appendix A, “Detailed Description of Study Visits”</td><td colspan="2">Change/rationale: The appendix was updated for the change in CD4 count frequency described above and to modify the window within which certain protocol visits could occur.</td></tr>
<tr><td>Throughout</td><td colspan="2">Minor editorial changes were made for clarity and consistency.</td></tr>
</table>

13 REFERENCES

1. Colditz G, Berkey C, Mosteller F, Brewer T, Wilson M, et al. The efficacy of Bacillus Calmette-Guerin vaccination in Newborns and Infants I the prevention of tuberculosis: meta-analysis of the published literature. Pediatrics 1995;96 (1): 29-35.

2. McShane H. Prime-boost immunization strategies for infectious diseases. Curr Opin Mol Ther. 2002a;4(1):23-7.

3. Skeiky YA, Sadoff JC. Advances in tuberculosis vaccine strategies. Nat Rev Microbiol. 2006;4(6):469-76.

4. Wiker HG, Nagai S, et al. A family of cross-reacting proteins secreted by Mycobacterium tuberculosis. Scand J Immunol. 1992;36(2):307-19.

5. Brooks JV, Frank AA, et al. Boosting vaccine for tuberculosis. Infect Immun. 2001;69(4):2714-7.

6. McShane H, Behboudi S, et al. Protective immunity against Mycobacterium tuberculosis induced by dendritic cells pulsed with both CD8(+)- and CD4(+)-T cell epitopes from antigen 85A. Infect Immun. 2002;70(3): 1623-6.

7. Huygen K, Content J, et al. Immunogenicity and protective efficacy of a tuberculosis DNA vaccine. Nat Med. 1996;2(8):893-8.

8. Baldwin SL, D'Souza C, et al. Evaluation of new vaccines in the mouse and guinea pig model of tuberculosis. Infect Immun. 1998;66(6):2951-9.

9. McShane H. Developing an improved vaccine against tuberculosis. Expert Rev Vaccines. 2004;3(3):299-306.

10. Tameris MD, Hatherill M, et al. Safety and efficacy of MVA85A, a new tuberculosis vaccine, in infants previously vaccinated with BCG: a randomised, placebo-controlled phase 2b trial. www.thelancet.com Published online February 4, 2013 http://dx.doi.org/10.1016/S0140-6736(13)60177-4.

11. Lachin JM: Maximum information designs. Clinical Trials. 2005; (2):453-464.

APPENDIX A Detailed Description of Study Visits

Screening Visit *Evaluations to be conducted within 45 days prior to randomization*

- Written informed consent process
- Assignment of screening number
- Medical history
- Physical examination
- Vital signs
- Chest X-ray
- Urine collection for urine dipstick
- Urine for βHCG (*all females physically capable of pregnancy*)
- Blood collection for
    - Serum chemistry (GGT, ALT, AST, total bilirubin, ALP, creatinine)
    - Hematology (CBC, differential, platelets)
    - QuantiFERON®-TB Gold In-Tube (***NOTE: Blood sample must collected prior to Tuberculin PPD skin test)***
    - CD4+ lymphocyte count: *Subjects on ART must have 2 CD4+ lymphocyte count test results >300 cells/mm^3, performed at least 4 weeks apart, one performed within 6 months prior to randomization and one within 45 days prior to randomization. Subjects NOT on ART must have 2 CD4+ lymphocyte count test results >350 cells/mm^3, performed at least 4 weeks apart, one performed within 6 months prior to randomization and one within 45 days prior to randomization*
    - Hepatitis B; HIV-1 viral load and HIV-1 Ab
- Tuberculin PPD skin test

Study Day 0 – Randomization/initial vaccination

Pre- vaccination:

- Confirmation that study entry eligibility criteria are met
    - Obtain pre-immunization vital signs
    - Urine βHCG (*all females physically capable of pregnancy*) – must be negative and must be from urine obtained within at least 2 days prior to visit
- Assign subject ID number (randomize)
- Blood collection for
    - CD4+ lymphocyte count *(not needed if already collected within 14 days of Study Day 0)*
    - HIV-1 viral load *(not needed if already collected within 14 days of Study Day 0)*
    - Immunology samples

Vaccination:

- Administer study vaccine by intradermal injection. Record date and time and location of vaccination.

Post-vaccination:

- Monitor subject for solicited, unsolicited, and serious adverse events for at least 60 minutes after vaccination (including concomitant medications)
- Obtain 60-minute post-immunization vital signs
- Complete site of injection examination
- Distribute diary cards and review diary card instructions

Study Visit 1 - *Allowable window for clinic visit is 7 ±2 days from Study Day 0 (Randomization/initial vaccination).*

- Obtain vital signs
- Review and record any solicited, unsolicited, and serious adverse events (including concomitant medications)
- Monitor subject for clinical observations for signs and symptoms of TB
- Review and record diary card entries for completeness and accuracy
- Complete site of injection examination
- Blood collection for
  - Serum chemistry (GGT, ALT, AST, total bilirubin, ALP, creatinine)
  - Hematology (CBC, differential, platelets)
  - CD4+ lymphocyte count
  - HIV-1 viral load
  - Immunology samples

Study Visit 2 - *Allowable window for clinic visit is 28 ±3 days from Study Day 0 (Randomization/initial vaccination).*

- Obtain vital signs
- Review and record any solicited, unsolicited, and serious adverse events (including concomitant medications)
- Monitor subject for clinical observations for signs and symptoms of TB
- Complete site of injection examination
- Blood collection for
  - Serum chemistry (GGT, ALT, AST, total bilirubin, ALP, creatinine)
  - Hematology (CBC, differential, platelets)
  - CD4+ lymphocyte count
  - HIV-1 viral load
  - Immunology samples

Study Visit 3 - *Allowable window for clinic visit is 84±7 days from Study Day 0 (Randomization/initial vaccination).*

- Obtain vital signs
- Monitor subject for clinical observations for signs and symptoms of TB
- Review and record any serious adverse events (including concomitant medications)
- Blood collection for
  - CD4+ lymphocyte count

Study Visit 4 – Booster Vaccination- *Allowable window for visit is 168 to 252 days from Study Day 0 (Randomization/initial vaccination).*

Pre- vaccination:

- Assessment of the subject's current health status and medical history to determine if there are any medical contraindications to receiving a booster vaccination
  - Urine βHCG (*all females physically capable of pregnancy*) – must be negative and must be from urine obtained within at least 2 days prior to visit
- Monitor subject for clinical observations for signs and symptoms of TB
- Obtain pre-immunization vital signs
- Blood collection for
  - Serum chemistry (GGT, ALT, AST, total bilirubin, ALP, creatinine)
  - Hematology (CBC, differential, platelets)
  - CD4+ lymphocyte count *(not needed if already collected within 14 days of visit)*
  - HIV-1 viral load *(not needed if already collected within 14 days of visit)*
  - Immunology samples

Vaccination:

- Administer study vaccine by intradermal injection. Record date and time and location of vaccination.

Post-vaccination:

- Monitor subject for solicited, unsolicited, and serious adverse events for at least 60 minutes after vaccination (including concomitant medications)
- Obtain 60-minute post-immunization vital signs
- Complete site of injection examination
- Distribute diary cards and review diary card instructions

Study Visit 5 - *Allowable window for clinic visit is 7 ±2 days from SV4 (booster vaccination visit).*

- Obtain vital signs
- Review and record any solicited, unsolicited, and serious adverse events (including concomitant medications)
- Monitor subject for clinical observations for signs and symptoms of TB
- Review and record diary card entries for completeness and accuracy
- Complete site of injection examination
- Blood collection for
  - Serum chemistry (GGT, ALT, AST, total bilirubin, ALP, creatinine)
  - Hematology (CBC, differential, platelets)
  - CD4+ lymphocyte count
  - HIV-1 viral load
  - Immunology samples

Study Visit 6 - *Allowable window for clinic visit is 28 ±3days from SV4 (booster vaccination visit).*

- Obtain vital signs
- Review and record any solicited, unsolicited, and serious adverse events (including concomitant medications)
- Monitor subject for clinical observations for signs and symptoms of TB
- Complete site of injection examination
- Blood collection for
  - Serum chemistry (GGT, ALT, AST, total bilirubin, ALP, creatinine)
  - Hematology (CBC, differential, platelets)
  - CD4+ lymphocyte count
  - HIV-1 viral load
  - Immunology samples

Study Visit 7 - *Allowable window for clinic visit is 84±7 days from SV4 (booster vaccination visit).*

- Obtain vital signs
- Monitor subject for clinical observations for signs and symptoms of TB
- Review and record any serious adverse events (including concomitant medications)
- Blood collection for
  - CD4+ lymphocyte count

Study Visit 8 until End of Study Visit – *Allowable window for clinic visit is every 3 months ±7 days from SV4 (booster vaccination visit)*

- Obtain vital signs
- Monitor subject for clinical observations for signs and symptoms of TB
- Review and record any serious adverse events (including concomitant medications)
- Blood collection for
    - CD4+ lymphocyte count (at 6 months post-boost)
    - HIV-1 viral load (at 6 months post-boost)

End-of-Study Visit

- Obtain vital signs
- Medical history
- Physical examination
- Monitor subject for clinical observations for signs and symptoms of TB
- Review and record any serious adverse events (including concomitant medications)
- Blood collection for
    - QuantiFERON®-TB Gold In-Tube (***NOTE: Blood sample must collected prior to Tuberculin PPD skin test)***
- Tuberculin PPD skin test

APPENDIX B SAE Reporting Scheme

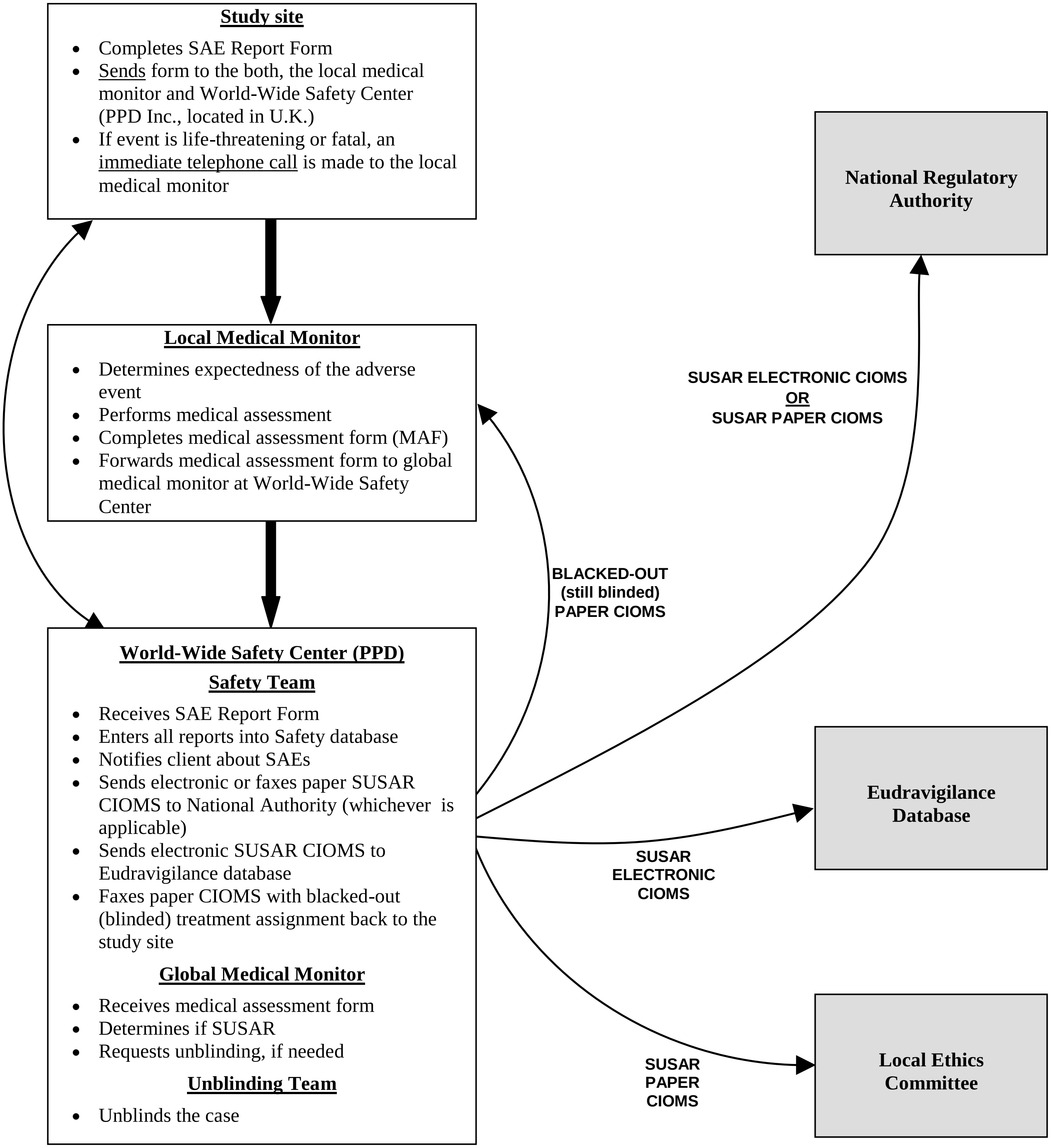

APPENDIX C Toxicity Table

From draft FDA guidance Toxicity Grading Scale for Healthy Adult and Adolescent Subjects Enrolled in Preventive Vaccine Clinical Trials (April 2005)

Local Site of Injection Symptoms	Mild (Grade 1)	Moderate (Grade 2)	Severe (Grade 3)	Potentially Life Threatening (Grade 4)
Pain	Does not interfere with activity	Interferes with activity or repeated use of non-narcotic pain reliever	Prevents daily activity or repeated use of narcotic pain reliever	ER visit or hospitalization
Tenderness	Mild pain to touch	Pain with movement	Significant pain at rest	ER visit or hospitalization
Redness *	2.5 - 5 cm	5.1 - 10 cm	>10 cm	Necrosis or exfoliative dermatitis
Swelling **	2.5 - 5 cm and does not interfere with activity	5.1 - 10 cm or interferes with activity	>10 cm or prevents daily activity	Necrosis

* Redness should be graded by measuring the greatest single diameter; measurement should be recorded as a continuous variable.
** Swelling should be evaluated and graded using the functional scale as well as the actual measurement.

Vital Signs*	Mild (Grade 1)	Moderate (Grade 2)	Severe (Grade 3)	Potentially Life Threatening (Grade 4)
Fever**	38.0 – 38.4°C<br>100.4 – 101.1°F	38.5 - 38.9°C<br>101.2 - 102.0°F	39.0 - 40°C<br>102.1 - 104°F	>40°C<br>>104°F
Tachycardia – beats per minute	101 – 115	116 – 130	>130	ER visit or hospitalization for arrhythmia
Bradycardia – beats per minute	50 – 54	45 – 49	<45	ER visit or hospitalization for arrhythmia
Hypertension (systolic) – mm Hg	141 – 150	151 – 155	>155	ER visit or hospitalization for malignant hypertension
Hypertension (diastolic) – mm Hg	91 – 95	96 – 100	>100	ER visit or hospitalization for malignant hypertension
Hypotension (systolic) – mm Hg	85 - 89	80 – 84	<80	ER visit or hospitalization for hypotensive shock
Respiratory rate – breaths per minute	17 – 20	21 – 25	>25	Intubation

* Subject should be at rest for all vital sign measurements.
** Oral temperature; no recent hot or cold beverages or smoking.

Systemic (General)	Mild (Grade 1)	Moderate (Grade 2)	Severe (Grade 3)	Potentially Life Threatening (Grade 4)
Nausea/vomiting	No interference with activity or 1 - 2 episodes/24 hours	Some interference with activity or >2 episodes/24 hours	Prevents daily activity, requires outpatient IV hydration	ER visit or hospitalization for hypotensive shock
Diarrhea	2 - 3 loose stools or < 400 grams/ 24 hours	4 - 5 stools or 400 - 800 grams/24 hours	6 or more watery stools or > 800 grams/24 hours or requires outpatient IV hydration	ER visit or hospitalization for hypotensive shock
Headache	No interference with activity	Some interference with activity or repeated use of non-narcotic pain reliever	Significant, prevents daily activity or repeated use of narcotic pain reliever	ER visit or hospitalization
Fatigue	No interference with activity	Some interference with activity	Significant, prevents daily activity	ER visit or hospitalization
Myalgia	No interference with activity	Some interference with activity	Significant, prevents daily activity	ER visit or hospitalization

Systemic Illness	Mild (Grade 1)	Moderate (Grade 2)	Severe (Grade 3)	Potentially Life Threatening (Grade 4)
Illness or clinical adverse event (as defined in protocol)	No interference with activity	Some interference with activity not requiring medical intervention	Prevents daily activity and requires medical intervention	ER visit or hospitalization

Serum*	Mild (Grade 1)	Moderate (Grade 2)	Severe (Grade 3)	Potentially Life Threatening (Grade 4)
Sodium – hyponatremia mEq/L	132 – 134	130 – 131	125 – 129	<125 or abnormal sodium with clinical signs
Sodium – hypernatremia mEq/L	144 – 145	146 – 147	148 - 150	>150
Potassium – hyperkalemia mEq/L	5.1 – 5.2	5.3 – 5.4	5.5 – 5.6	>5.6
Potassium – hypokalemia mEq/L	3.5 – 3.6	3.3 – 3.4	3.1 – 3.2	<3.1
Glucose – hypoglycemia mg/dL	65 – 69	55 – 64	45 – 54	<45
Glucose – hyperglycemia<br>Fasting - mg/dL<br>Random – mg/dL	100 – 110<br>110 - 125	111 – 125<br>126 – 200	>125<br>>200	Insulin requirements or hyperosmolar coma
Blood urea nitrogen BUN mg/dL	23 – 26	27 – 31	>31	Requires dialysis
Creatinine – mg/dL	1.1 – 1.5	1.6 – 2.0	2.1 – 2.5	>2.5 or requires dialysis
Calcium – hypocalcemia mg/dL	8.0 – 8.4	7.5 – 7.9	7.0 – 7.4	<7.0
Calcium – hypercalcemia mg/dL	10.5 – 11.0	11.1 – 11.5	11.6 – 12.0	>12.0
Magnesium – hypomagnesemia mg/dL	1.3 – 1.5	1.1 – 1.2	0.9 – 1.0	<0.9
Phosphorous – hypophosphatemia mg/dL	2.3 – 2.5	2.0 – 2.2	1.6 – 1.9	<1.6
Albumin – hypoalbuminemia g/dL	2.8 – 3.1	2.5 – 2.7	<2.5	----
Total protein – hypoproteinemia g/dL	5.5 – 6.0	5.0 – 5.4	<5.0	----
Alkaline phosphate – increase by factor	1.1 – 2.0 x ULN	2.1 – 3.0 x ULN	3.1 – 10 x ULN	>10 x ULN
Liver Function Tests (LFT) – ALT, AST increase by factor	1.1 – 2.5 x ULN	2.6 – 5.0 x ULN	5.1 – 10 x ULN	>10 x ULN
Bilirubin – when accompanied by any increase in LFT increase by factor	1.1 – 1.25 x ULN	1.26 – 1.5 x ULN	1.51 – 1.75 x ULN	>1.75 x ULN
Bilirubin – when LFT is normal increase by factor	1.1 – 1.5 x ULN	1.6 – 2.0 x ULN	2.1 – 3.0 – x ULN	>3.0 x ULN
Cholesterol	201 – 210	211 – 225	>226	----
Pancreatic enzymes – amylase, lipase	1.1 – 1.5 x ULN	1.6 – 2.0 x ULN	2.1 – 5.0 x ULN	>5.0 x ULN

*ULN (upper limit of normal) dependent on normal reference ranges per institutional parameters.

Hematology*	Mild (Grade 1)	Moderate (Grade 2)	Severe (Grade 3)	Potentially Life Threatening (Grade 4)
Hemoglobin** (Female) – gm/dL	12.0 – 13.0	10.0 – 11.9	8.0 – 9.9	<8.0
Hemoglobin** (Male) – gm/dL	12.5 – 14.5	10.5 – 12.4	8.5 – 10.4	<8.5
WBC Increase – cell/mm^3	10800 – 15000	15001 – 20000	20001 – 25000	>25000
WBC Decrease – cell/mm^3	2500 – 3500	1500 – 2499	1000 – 1499	<1000
Lymphocytes Decrease – cell/mm^3	750 - 1000	500 – 749	250 – 499	<250
Neutrophils Decrease – cell/mm^3	1500 – 2000	1000 – 1499	500 – 999	<500
Eosinophils – cell/mm^3	650 – 1500	1501 – 5000	>5000	Hypereosinophilic syndrome
Platelets Decrease – cell/mm^3	125000 – 140000	100000 – 124000	25000 – 99000	<25000
PT – increase by factor (prothrombin time)	1.0 – 1.10 x ULN	1.11 – 1.20 x ULN	1.21 – 1.25 x ULN	>1.25 x ULN
PTT – increase by factor (partial thromboplastin time)	1.0 – 1.2 x ULN	1.21 – 1.4 x ULN	1.41 – 1.5 x ULN	>1.5 x ULN
Fibrinogen increase – mg/dL	400 – 500	501 - 600	>600	----
Fibrinogen decrease – mg/dL	150 – 200	125 – 149	100 - 124	<100 or associated with gross bleeding or disseminated intravascular coagulation (DIC)

* ULN (upper limit of normal) dependent on normal reference ranges per institutional parameters.
**For this study, abnormal hemoglobin results will be graded using the absolute value ranges in the table not change from baseline.

Urine	Mild (Grade 1)	Moderate (Grade 2)	Severe (Grade 3)	Potentially Life Threatening (Grade 4)
Protein	Trace	1+	2+	>2+
Glucose	Trace	1+	2+	>2+
Blood (microscopic) – red blood cells per high power field (rbc/hpf)	1 – 10	11 – 50	>50 and/or gross blood	Hospitalization or packed red blood cells (PRBC) transfusion

APPENDIX D Phlebotomy Volumes

Specimen	Tube	Volume per collection (mL)	Number of Collections Thru EoS	Maximum Total collected (mL)
QuantiFERON®-TB Gold In Tube	QFT-TB Gold IT collection tubes (3)	3	2	6
Hep B Ab	7.0 ml BD Gold or Red/black Vacutainer®	7	1	7
HIV-1 ELISA and confirmatory PCR	7.0 ml BD Gold or Red/black Vacutainer®	5	1	5
HIV-1 viral load	5.0 ml BD Lavender Vacutainer® (or Gold or Red Vacutainer®)	5	8	40
CD4+ lymphocyte count	5.0 ml BD Lavender Vacutainer® (or Gold or Red Vacutainer®)	5	10	50
Serum Chemistry	7.0 ml BD Red or Green Vacutainer®	7	6	42
CBC, differential, platelets	5.0 ml BD Lavender Vacutainer®	5	6	30
Immunology Cells and Plasma	8.0 ml BD CPT™ (4)	36	6	216
WB-ICS ("real-time" immunology cohort only)	5.0 ml sodium heparin tube (1)	4	4	16
**Estimated maximum total blood collected per protocol**				**396-412**

Refer to Schedule of Evaluations (Table 3-1 and 3-2) for estimated blood volumes collected per study visit.

APPENDIX E Subject Diary (Template)

Subject Number

Please fill in every box and do not leave any blank

			Have these happened since the last time you recorded your observations?				Measurement of largest (mm)	
Day after Vaccine	Date	Temperature in armpit (°C)	Injection site pain (circle maximum score)	Warmth	Itching	Scaling	Redness	Swelling
0	DD/MM/YYYY		0 1 2 3	Yes / No	Yes / No	Yes / No		
1	DD/MM/YYYY		0 1 2 3	Yes / No	Yes / No	Yes / No		
2	DD/MM/YYYY		0 1 2 3	Yes / No	Yes / No	Yes / No		
3	DD/MM/YYYY		0 1 2 3	Yes / No	Yes / No	Yes / No		
4	DD/MM/YYYY		0 1 2 3	Yes / No	Yes / No	Yes / No		
5	DD/MM/YYYY		0 1 2 3	Yes / No	Yes / No	Yes / No		
6	DD/MM/YYYY		0 1 2 3	Yes / No	Yes / No	Yes / No		
7	DD/MM/YYYY		0 1 2 3	Yes / No	Yes / No	Yes / No		

Injection Site Pain Score
0 = No pain
1 = Sensitive to touch
2 = Partial restriction of activity
3 = Pain restricts activity

Measure the size of redness and swelling by putting the ruler through both vaccine sites as shown (photo right). Record only the largest measurements at either vaccine site.

Record all signs you have experienced since the last time you recorded them, up to and including the time you take the measurements. Try to record everything at approximately the same time each day.

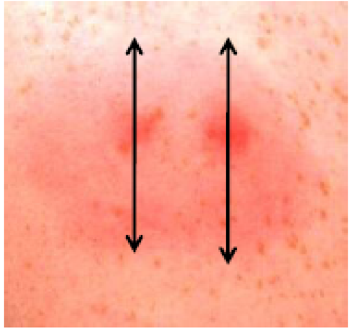

Subject Number

Please indicate if you have experienced any of these symptoms since you last recorded your symptoms.

Circle 'yes' or 'no' and, if yes, please also write the number of times

		If these are only present at the injection site then please record as pain on page 2						
Day after Vaccine	Date	General muscle ache	General join pain	Unwell	More tired than usual	Headache	Nausea	Vomiting
0	DD/MM/YYYY	Yes / No	Yes / No	Yes / No	Yes / No	Yes / No	Yes / No	Yes / No
1	DD/MM/YYYY	Yes / No	Yes / No	Yes / No	Yes / No	Yes / No	Yes / No	Yes / No
2	DD/MM/YYYY	Yes / No	Yes / No	Yes / No	Yes / No	Yes / No	Yes / No	Yes / No
3	DD/MM/YYYY	Yes / No	Yes / No	Yes / No	Yes / No	Yes / No	Yes / No	Yes / No
4	DD/MM/YYYY	Yes / No	Yes / No	Yes / No	Yes / No	Yes / No	Yes / No	Yes / No
5	DD/MM/YYYY	Yes / No	Yes / No	Yes / No	Yes / No	Yes / No	Yes / No	Yes / No
6	DD/MM/YYYY	Yes / No	Yes / No	Yes / No	Yes / No	Yes / No	Yes / No	Yes / No
7	DD/MM/YYYY	Yes / No	Yes / No	Yes / No	Yes / No	Yes / No	Yes / No	Yes / No

Subject Number

Please list any medicines you take during these seven days – or bring them with you to your appointment and tell the nurse

Symptoms	Start Date	End Date	Medicine taken (include amount and how often you took them)
	DD/MM/YYYY	DD/MM/YYYY	
	DD/MM/YYYY	DD/MM/YYYY	
	DD/MM/YYYY	DD/MM/YYYY	
	DD/MM/YYYY	DD/MM/YYYY	
	DD/MM/YYYY	DD/MM/YYYY	
	DD/MM/YYYY	DD/MM/YYYY	
	DD/MM/YYYY	DD/MM/YYYY	
	DD/MM/YYYY	DD/MM/YYYY	

Please use this table to describe any other symptoms you have experienced during these seven days, even if you do not think they are anything to do with the vaccine, and record any medicines you have taken (remedies, herbal preparations, creams, ointments etc)

C-030-485/ TB021 – Diary Card – Version 2.0 – 9 November 2011
